# The Influence of Alkyl Spacers and Molecular Weight on the Charge Transport and Storage Properties of Oxy‐Bithiophene‐Based Conjugated Polymers

**DOI:** 10.1002/anie.202417897

**Published:** 2024-12-23

**Authors:** Hang Yu, Adam Marks, Sachetan M. Tuladhar, Nicholas Siemons, Iona Anderson, Sophia Bidinger, Scott T. Keene, Tyler J. Quill, Ruiheng Wu, Olivia Gough, Guanchen Wu, Flurin Eisner, Alberto Salleo, Jonathan Rivnay, George G. Malliaras, Piers R. F. Barnes, Iain McCulloch, Jenny Nelson

**Affiliations:** ^1^ Department of Physics and Centre for Processable Electronics Imperial College London SW7 2AZ London United Kingdom; ^2^ Department of Chemistry University of Oxford OX1 2JD Oxford United Kingdom; ^3^ Department of Materials Science and Engineering Stanford University 94305 Stanford CA United States; ^4^ Department of Engineering University of Cambridge CB3 0FA Cambridge United Kingdom; ^5^ Department of Biomedical Engineering Northwestern University 60208 Evanston IL United States; ^6^ Andlinger Center for Energy and the Environment Department of Electrical and Computer Engineering Princeton University 08544 Princeton NJ United States

**Keywords:** Conformation analysis, Conjugated polymers, Electrochemistry, Molecular dynamics, Polymer-solvent interactions

## Abstract

Conjugated polymers (CPs) with polar side chains can conduct electronic and ionic charges simultaneously, making them promising for bioelectronics, electrocatalysis and energy storage. Recent work showed that adding alkyl spacers between CP backbones and polar side chains improved electronic charge carrier mobility, reduced swelling and enhanced stability, without compromising ion transport. However, how alkyl spacers impact polymer backbone conformation and, subsequently, electronic properties remain unclear. In this work, we design two oxy‐bithiophene‐based CP series, each featuring progressively extended alkyl spacer lengths and two distinct molecular weight (MW) distributions. Using *operando* characterisations, we evaluate the (spectro)electrochemical and swelling properties of the polymer thin films, and their performance in organic field‐effect transistors and organic electrochemical transistors. Surprisingly, alkyl spacers negatively impact the hole mobility of our polymers, with higher MW amplifying this effect. Using molecular dynamics simulations, we show that it is thermodynamically favourable for adjacent non‐polar alkyl spacers to aggregate in polar electrolytes, leading to backbone twisting. Further spectroscopic measurements corroborate this prediction. Our findings demonstrate the active interactions between side chain structure, MW and electrolyte/solvent polarity in influencing polymer performance, underscoring the importance of considering solvation environment effects on polymer conformation when designing new mixed conducting CPs for electrochemical applications.

## Graphical abstract

A mismatch in polarity between polymer side chains and their solvation environment can lead to segmental aggregation, causing the flexible backbone to twist and, subsequently, deteriorate the polymer‘s electronic properties.

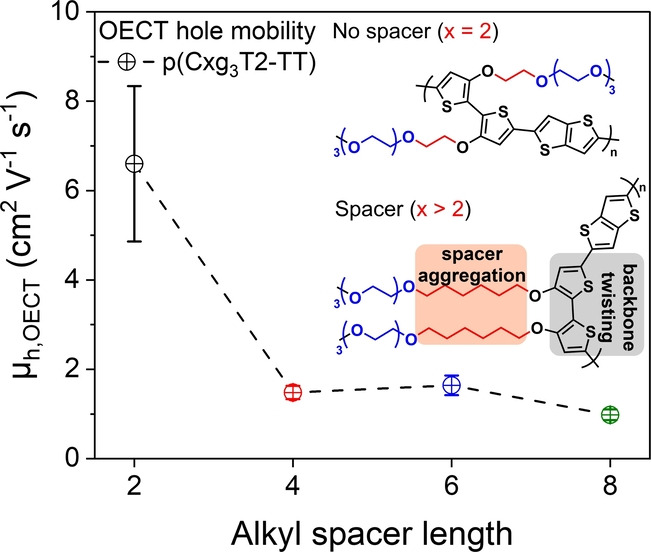

## Introduction

Conjugated polymers (CPs) with polar side chains are a type of organic mixed ionic‐electronic conductors (OMIECs) which is solution‐processable, redox‐active and capable of conducting ionic and electronic charges simultaneously.^[1]^ The introduction of polar side chains, typically oligoethylene glycol, facilitates fast ion transport within the CPs and efficient ion exchange with aqueous electrolytes during charging (doping)/discharging (dedpoing). Consequently, these materials find their applications in scenarios requiring mixed conduction in aqueous environments, e.g. bioelectronics, electrochromics, electrocatalysis and electrochemical energy storage.[^2^, ^3^, ^4^, ^5^, ^6^] However, CPs with a high density of polar side chains often exhibit suboptimal device performance, such as low electronic charge mobility and low transconductance in organic electrochemical transistors (OECTs), as well as poor mechanical and electrochemical stability that may result in delamination or irreversible electrochemical processes.[^7^, ^8^, ^9^] These challenges may be a consequence of their excessive electrolyte uptake during the electrochemical charging (doping) processes. One strategy to address this is to modify the side chains by adjusting their length, density and polarity to control the swelling and improve the electrochemical properties of mixed conducting CPs.^[10]^

Current side chain engineering strategies to mitigate the above challenges include partially substituting polar glycol side chains with non‐polar alkyl side chains, adjusting glycol side chain lengths, introducing alkyl spacers to separate glycol blocks from the conjugated backbone as well as replacing the ethylene glycol units with less polar propylene and butylene glycol.[^7^, ^8^, ^11^, ^12^, ^13^] Among these strategies, the introduction of alkyl spacers was reported to effectively improve hole (electron) mobility in p‐type (n‐type) mixed conducting polymers in OECTs and to reduce their swelling under electrochemical bias. For example, Maria *et al*. reported a nine‐fold improvement in electron mobility in OECTs by incorporating six methylene groups (alkyl spacer length = 6) on the naphthalenediimide (NDI) units of a benchmark fully glycolated polymer, p(gNDI‐gT2).^[14]^ This modification resulted in the maximum swelling of the modified polymer decreasing by 50 % compared with p(gNDI‐gT2). In the case of p‐type polymers, Schmode *et al*. modified the archetypal thiophene‐based P3MEET polymer by inserting a methyl spacer (P3MEEMT) or an ethyl spacer (P3MEEET) into the glycol side chains, resulting in an increase in hole mobility by two orders of magnitude in OECTs.^[15]^

**Figure 1:**
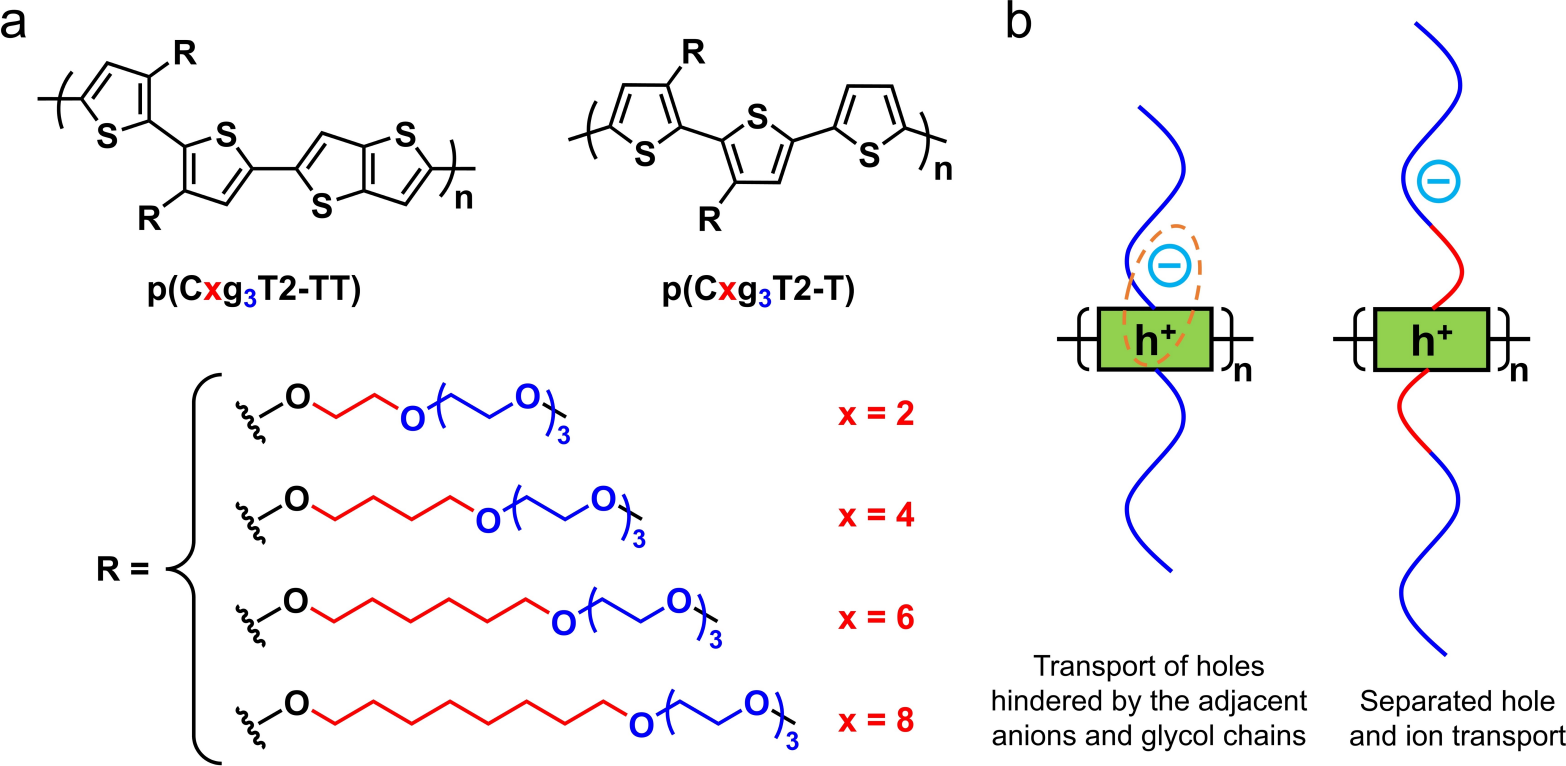
(a) Chemical structure of the p(Cxg_3_T2‐TT) and p(Cxg_3_T2‐T) polymers featuring alkyl spacers of progressively varied lengths (Cx, x = 2, 4, 6, 8) positioned between the backbone and the methyl end‐capped triethylene glycol block (g_3_). (b) Schematic representation of the design strategy.

The effect of alkyl spacers in improving electronic charge carrier mobility has been attributed to the increased separation between electronic charges on backbones and the solvated ions near the glycol blocks of the side chains, which weakens their electrostatic interaction, as illustrated in Figure 1b.^[16]^ However, the alkyl spacers must also influence the microstructure of the polymers, yet how and to what extent is not well understood. For example, little is known about how the alkyl spacers impact the backbone conformation of CPs, yet conformation directly affects the electronic properties of the conjugated π system. Ohayon *et al*. found that in p(gNDI‐gT2), the OECT performance was enhanced when progressively elongated alkyl spacers were synthesised on the gNDI units, but was lowered when the same alkyl spacers were introduced to the gT2 units.^[12]^ They attributed the improved electron mobility to a higher degree of lamellar ordering, as supported by grazing‐incidence wide‐angle X‐ray scattering (GIWAXS) experiments. However, GIWAXS, whether *ex situ* or *operando*, monitors structural changes in the crystalline phase, and cannot provide information on structural changes in the amorphous phase of the polymer which is more likely to be penetrated by ions.[^17^, ^18^, ^19^] To understand the structure–property relationship at the single‐chain or amorphous film level, molecular dynamics (MD) simulations provide an accessible and flexible tool.[^20^, ^21^, ^22^, ^23^]

Apart from chemical structure, the polymer molecular weight (MW) can also strongly influence the properties and performance of CPs. It is known that a higher MW can enhance the charge transport properties in solid‐state organic semiconductor devices due to long polymer chains bridging the adjacent crystalline phase.[^24^, ^25^, ^26^] However, less is known about the impact of MW on the performance of mixed conducting polymers, which undergo dynamic bulk electrolyte uptake/expulsion during operation. Recently, a few studies were reported to address the influence of MW on the performance of OECTs. One study using BBL, an archetypal n‐type CP, as the OECT channel material showed that the electron mobility was improved by one order of magnitude as the number‐average MW of BBL increased from 4.9 to 51 kDa.^[27]^ Another study on a model p‐type polymer, P3MEEET, showed that increasing MW could generally improve the OECT performance, with the extent of improvement being electrolyte‐dependent.^[19]^ In addition, a study exploring the influence of residual palladium and MW on the OECT performance suggested that a moderately high MW (50 kDa for p(g_4_T2‐TT) and 100 kDa for the n‐type p(C_6_g_3_NDI‐T)) offered the highest hole/electron mobility, whilst excessively high MW adversely affected the electronic charge transport.^[28]^ These results suggest a generally positive impact of high MW on OECT performance, with an optimum MW for some polymers. Beyond these, the relationship between MW, microstructure and electronic properties has not been studied in depth.

In this work, we investigate the impact of both side chain structure, including alkyl spacers, and polymer MW on the charge transport and accumulation properties of oxy‐bithiophene‐based CPs. Surprisingly, increasing alkyl spacer length results in an improvement in the OFET hole mobility but a decline in OECT hole mobility. It also results in a decrease in specific capacitance. Notably, high MW amplifies these negative effects. These observations, where alkyl spacer and high MW deteriorate OECT performance, contradict previous reports. Using MD simulations and UV‐Vis spectroscopy we rationalise the behaviour in terms of the relative strength of interactions between polar and non‐polar side chain units and the surrounding solvent, which can lead to backbone twisting for polymers with alkyl spacers in polar environments. The results show that the polymer's torsional flexibility and solvent environment are important factors in predicting the impact of amphiphilic side chains.

## Results and Discussion

To evaluate the influence of alkyl spacers on the charge transport and storage properties of oxy‐bithiophene‐based CPs, we designed and synthesised a series of four p‐type CPs based on a 3,3’‐dialkoxybithiophene‐thienothiophene backbone. The side chains feature progressively extended alkyl spacers (Cx, number of methylene groups) positioned between the backbone and the methyl end‐capped triethylene glycol block (g_3_). The polymer series is denoted as p(Cxg_3_T2‐TT) (x = 2, 4, 6, 8; Figure 1a). It should be noted that the benchmark polymer, p(C2g_3_T2‐TT), is attached with methyl end‐capped tetraethylene glycol side chains that do not incorporate an alkyl spacer. The aim of this design strategy is illustrated in Figure 1b and discussed in the Introduction.

Gel permeation chromatography (GPC) measurements show that the number average molecular weights (*M*_n_) of the p(Cxg_3_T2‐TT) series are 83.4, 130.5, 114.3 and 110.6 kDa for p(Cxg_3_T2‐TT) (x = 2, 4, 6, 8), respectively. Although the influence of aggregation on the measured *M*_n_ could not be ruled out, these high *M*_n_ values indicate that all four polymers have an average chain length exceeding 100 repeating units, given their monomer MWs of 714.9, 771.0, 827.2, and 883.3 Da, respectively. Additionally, the polymers in the p(Cxg_3_T2‐TT) series show similar ionisation potentials (IPs) of 4.3 – 4.4 eV (Table 1), as measured by photoelectron spectroscopy in air (PESA). The comparable IPs and *M*_n_ across the four polymers ensure that the differences in their charge transport and electrochemical properties can be assigned to the difference in the alkyl spacer length rather than to MW or backbone differences.

**Table 1:** Summary of the polymers’ properties.

Polymer	*M*_n_^[a]^ (kDa) [*Đ*]	IP^[b]^ (eV)	*E*_ox,onset_^[c]^ (V)	*λ*_onset_^[d]^ (nm)
p(**C2**g_3_T2‐TT)	83.4 [1.36]	4.33	−0.06 (−0.06)	671
p(**C4**g_3_T2‐TT)	130.5 [2.06]	4.37	−0.03 (−0.03)	661
p(**C6**g_3_T2‐TT)	114.3 [2.07]	4.38	0.02 (0.02)	661
p(**C8**g_3_T2‐TT)	110.6 [2.55]	4.41	0.05 (0.06)	664
p(**C2**g_3_T2‐TT) L‐MW	7.1 [1.91]	4.52	−0.06 (−0.06)	662
p(**C4**g_3_T2‐TT) L‐MW	10.0 [2.14]	4.48	−0.07 (−0.06)	669
p(**C6**g_3_T2‐TT) L‐MW	11.6 [2.59]	4.43	−0.04 (−0.03)	671
p(**C8**g_3_T2‐TT) L‐MW	5.9 [1.92]	4.58	0.00 (−0.01)	667

[a] GPC measurements were carried out using low‐D (<1.10) polystyrene standards and DMF as the eluent at 40 °C. [b] Ionisation potential (IP) measured by photoelectron spectroscopy in air (PESA). [c] Oxidation onset potential of the polymer thin films vs Ag/AgCl in their first (and second in parentheses) CV scans (10 mV/s) in Ar‐saturated 0.1 M NaCl (aq) solutions. [d] Absorption onset of 0.1 mg/mL polymer solutions in chloroform.

To illustrate the impact of MW, we intentionally synthesised a control group of p(Cxg_3_T2‐TT) with significantly lower MW, denoted as p(Cxg_3_T2‐TT) L‐MW (x = 2, 4, 6, 8). In this control group, the *M*_n_ of the four polymers were measured as 7.1, 10.0, 11.6 and 5.9 kDa for x = 2, 4, 6 and 8, respectively (Table 1). These values are approximately one order of magnitude lower than their p(Cxg_3_T2‐TT) counterparts, indicating that p(Cxg_3_T2‐TT) L‐MW are oligomers in nature with an average of approximately only 10 repeat units. The synthesis of each polymer series is shown in the Supporting Information (SI) Section 10. Their chemical, electrical and optical properties are summarised in Table 1. In addition to these two polymer series, we synthesised another series of CPs for comparison, which is based on a 3,3’‐dialkoxybithiophene‐thiophene backbone with alkyl spacers on the side chain, denoted as p(Cxg_3_T2‐T) (x = 2, 4, 6, 8; Figure 1a). These polymers possess a lower degree of intrinsic collinearity than p(Cxg_3_T2‐TT) and therefore provide a useful test of the wider applicability of our findings. Since their properties are otherwise close to those of p(Cxg_3_T2‐TT) L‐MW (x = 2, 4, 6, 8), we provided their synthesis and property data only in the SI.

**Figure 2:**
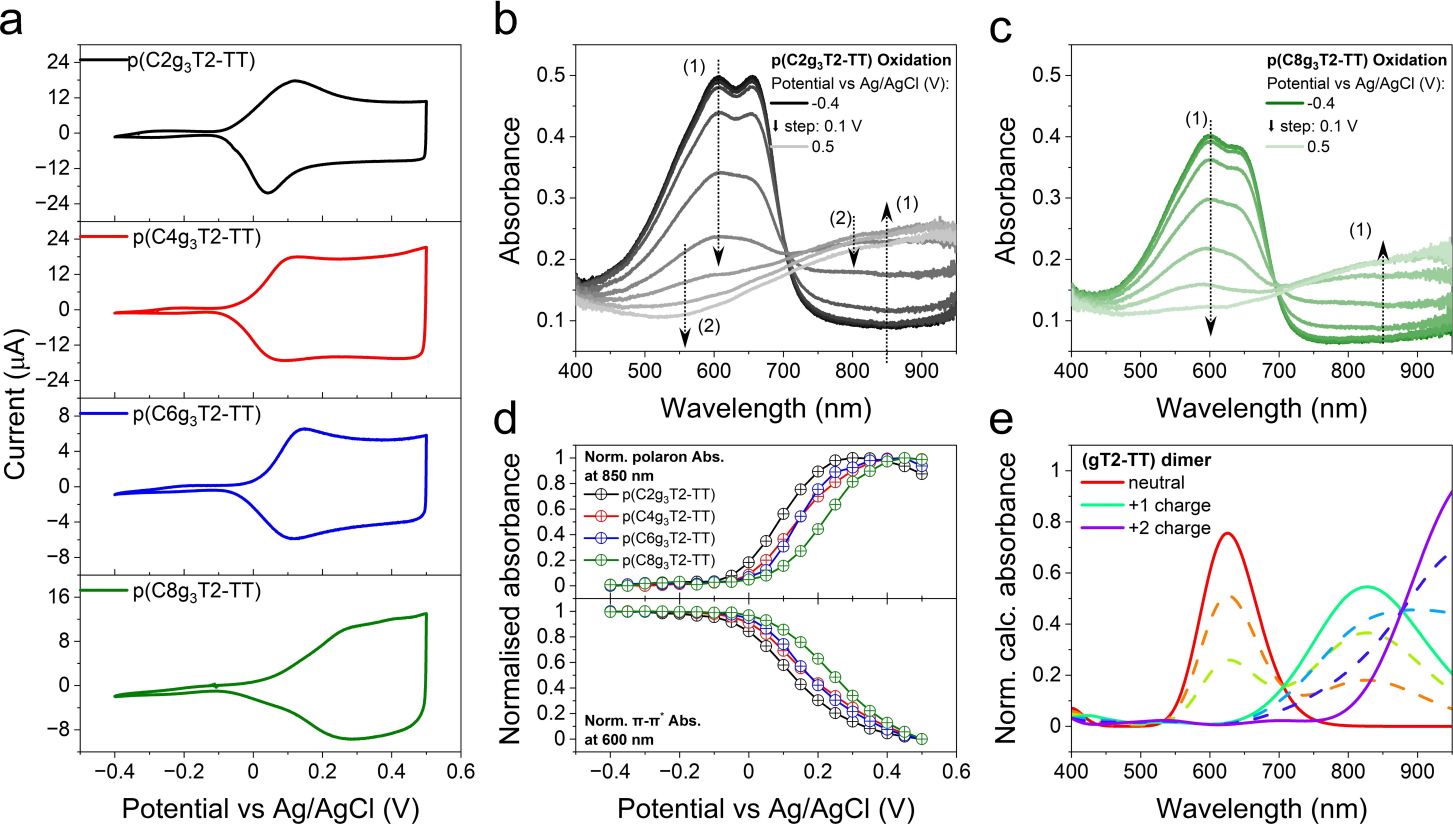
Electrochemical and spectroelectrochemical properties of the p(Cxg_3_T2‐TT) polymers. (a) CV measurements of p(Cxg_3_T2‐TT) polymer thin films on ITO glass substrates in Ar‐saturated 0.1 M NaCl (aq). The potential was scanned from −0.4 V to 0.5 V vs Ag/AgCl at a scan rate of 10 mV/s. The second CV cycle of the four polymer films is shown. (b, c) Changes in the absorbance spectra of (b) p(C2g_3_T2‐TT) and (c) p(C8g_3_T2‐TT) polymer thin films during the second CV oxidation process shown in (a). The spectra were recorded every 0.05 V but are presented here every 0.1 V. The notations (1) and (2) and the adjacent arrows refer to the spectra change due to the formation of hole polarons (1) and bipolarons (2). (d) The normalised absorbance change, relative to the absorbance at −0.4 V, in the intensity of the ground state absorption at 600 nm and the polaron absorption at 850 nm, recorded during the second CV oxidation process at intervals of 0.05 V. (e) Normalised calculated absorbance spectra using TD‐DFT with B3LYP/6–311+g(d,p) level of theory of the (gT2‐TT) dimer in the neutral (red) and charged (+1: hole polaron, green; +2: hole bipolaron, purple) states in a water (SMD) environment. Linear combinations (dash lines) of the absorbance spectra between the neutral and polaronic states and between the polaronic and bipolaronic states are shown to reproduce the experimental absorbance spectra change.

We first performed cyclic voltammetry (CV) measurements on the p(Cxg_3_T2‐TT) thin films in 0.1 M NaCl (aq) electrolytes to investigate the influence of the alkyl spacers on the redox properties of the polymers. As shown in Figure 2a, after the oxidation onset, all four polymers show one oxidation peak followed by a current plateau analogous to an electrochemical double‐layer capacitor. This current–voltage feature reverses when the potential scans back from 0.5 V to −0.4 V vs Ag/AgCl. As the alkyl spacer length increases from 2 to 8, i.e. for p(Cxg_3_T2‐TT) (x = 2 to 8), the oxidation onset potentials of the polymers (*E*_ox,onset_) and the oxidation peak potentials (*E*_p,ox_) shift towards more positive biases. Given that the four polymers share the same backbone structure, and thus similar electronic structures, the positive shifts in *E*_ox,onset_ and *E*_p,ox_ can be attributed to the decreasing hydrophilicity of the polymer thin films with increasing alkyl spacer length. The reduction in hydrophilicity necessitates a higher bias to drive anions to migrate into the polymer films for charge neutralisation. This observed peak shifting towards higher biases due to lower film hydrophilicity corroborates findings in the literature.[^7^, ^29^] The cyclic voltammograms of the other two polymer series exhibit similar redox features and potential shifts, as summarised in Figure S2.

As electrochemical doping can change the energies and occupation of molecular orbitals of mixed conducting polymers, and thus their absorption spectra, we performed *operando* UV‐Vis absorption measurements simultaneously during the CV measurements to probe the changes in the absorbance spectra of the p(Cxg_3_T2‐TT) thin films and quantify their depth of charging (doping). Figures 2b, c and S3 show that with increasing oxidising potentials, all four polymers lose their ground state absorption between 400 and 700 nm and develop a new absorption band in the near‐infrared (NIR) region. This phenomenon is assigned to the formation of hole polarons. Notably, the onset of polaron formation for each polymer is consistent with its *E*_ox,onset_ in the CV (Figure 2d). Further oxidation of p(C2g_3_T2‐TT) beyond 0.3 V vs Ag/AgCl and p(Cxg_3_T2‐TT) (x = 4, 6) beyond 0.4 V vs Ag/AgCl leads to a reduction in polaron absorbance in the NIR (Figures 2b, d and S3), suggesting the conversion of hole polarons to bipolarons. However, such a conversion is not observed for p(C8g_3_T2‐TT) under the highest applied bias of 0.5 V vs Ag/AgCl (Figure 2c).

To interpret the changes in absorption spectra under electrochemical bias, we performed time‐dependent density functional theory (TD‐DFT) calculations with B3LYP/6–311+g(d,p) level of theory on a (gT2‐TT)_2_ dimer (Figure S18), which represents the backbones of p(Cxg_3_T2‐TT) (x = 2, 4, 6, 8). The calculated absorption spectra of (gT2‐TT)_2_ in its neutral, polaronic and bipolaronic states (0, 1 and 2 holes per dimer, respectively) in a water (Solvation Model Based on Density, SMD) environment (Figure 2e) closely approximate the experimental ones, supporting our assignments of the neutral, polaronic and bipolaronic absorption in the experimental spectra, consistent with previous literature reports.[^5^, ^29^] Comparison of the calculations with the experimental spectroelectrochemical absorption spectra indicates that p(Cxg_3_T2‐TT) (x = 2, 4 and 6) can accept more than one hole per two repeat units (dimer) at 0.5 V vs Ag/AgCl. The experimental observation of reduced polaron absorbance around 850 nm correlates well with an evolution in the shape of the calculated absorbance spectra whereby a new absorption band gains strength and the original (polaron) band loses strength, resulting in a net redshift of the charged oligomer's absorption spectrum as it transitions from the +1 charge to +2 charge state. In comparison, p(C8g_3_T2‐TT) appears to accept only one hole per dimer, as this spectral transition is not observed in the experiment.

It is worth noting that there are debates on the naming and the spin nature of polarons and bipolarons in thiophene‐based p‐type CPs.[^30^, ^31^, ^32^] Cavassin *et al*. have made a comprehensive discussion on the previous work defining polarons and bipolarons.^[33]^ In this study, we adopt the assignment of the absorption band around 850 nm to polarons and an additional band in further NIR region to bipolarons, rather than attributing the evolution of these two bands to contributions from both polarons and bipolarons. The spectroelectrochemical data for p(Cxg_3_T2‐TT) L‐MW and p(Cxg_3_T2‐T) are summarised in SI section 3 (Figures S4 and S5) and computational details are summarised in SI section 8. Having analysed the (spectro)electrochemical properties of the p(Cxg_3_T2‐TT) polymer thin films, we now address their charge storage and transport properties, with the aim of understanding the impacts of alkyl spacer length and polymer MW.

**Figure 3:**
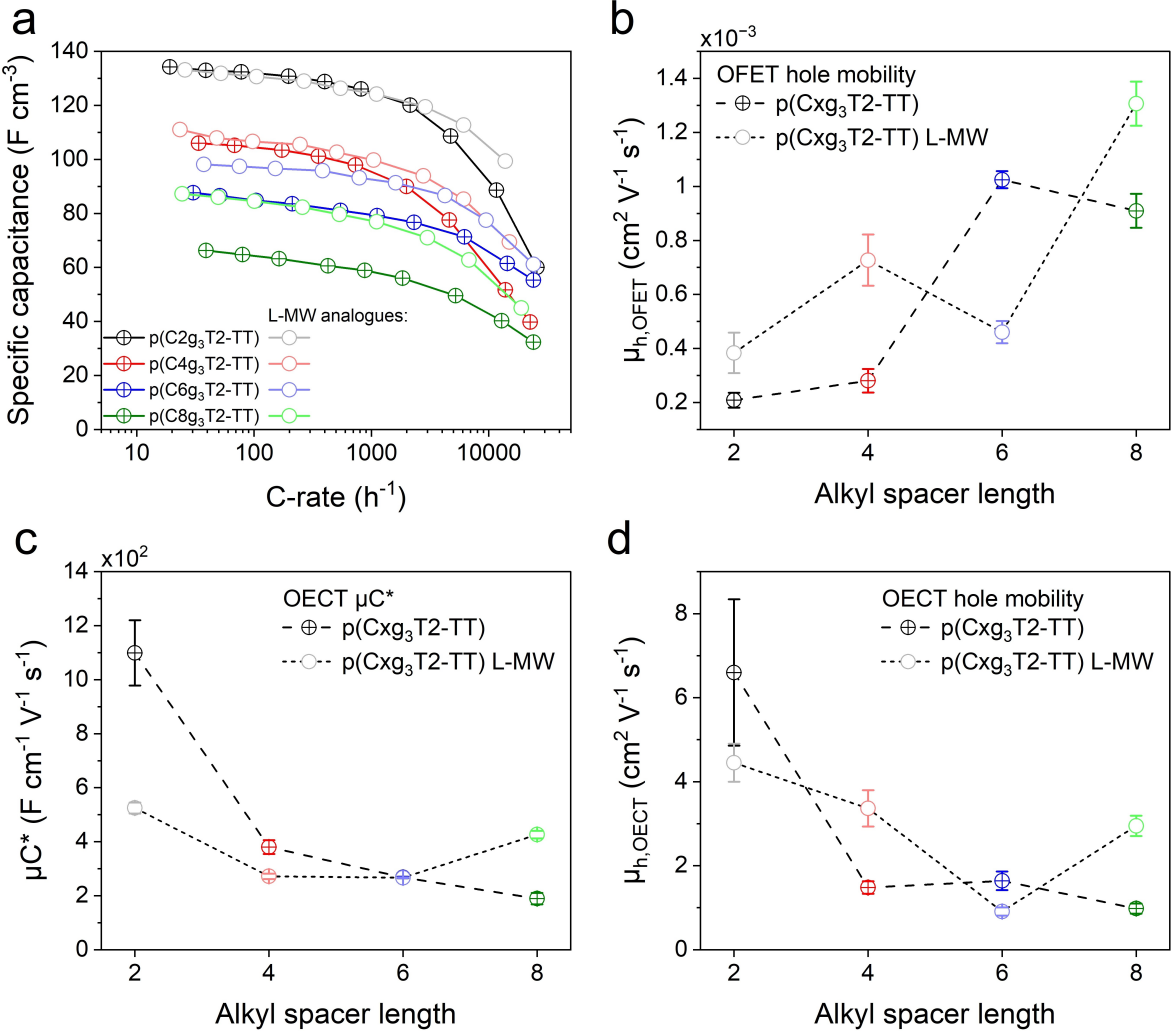
Charge storage and transport properties of the p(Cxg_3_T2‐TT) and p(Cxg_3_T2‐TT) L‐MW polymers. (a) Volumetric specific capacitance as a function of C‐rate for the p(Cxg_3_T2‐TT) (heavy symbols + lines) and p(Cxg_3_T2‐TT) L‐MW (light symbols + lines) polymers. (b) Hole mobilities (*μ*_h,OFET_) in the p(Cxg_3_T2‐TT) (heavy symbols + dash lines) and p(Cxg_3_T2‐TT) L‐MW (light symbols + short dash lines) polymers extracted from OFETs in the saturation regimes. The OFETs were fabricated on glass substrates adopting a top‐gate bottom‐contact configuration, with CYTOP serving as the dielectric. (c) The products of OECT hole mobilities and volumetric capacitance (μC*) and (d) OECT hole mobilities (*μ*_h,OECT_) of the OECTs using p(Cxg_3_T2‐TT) (heavy symbols + dash lines) and p(Cxg_3_T2‐TT) L‐MW (light symbols + short dash lines) polymers as the channel materials. The μC* values were extracted from the linear fitting of the plots of maximum transconductance (*g*_m_) vs channel geometry and operation parameters (Figures S12e and S14e). The *g*_m_ values were calculated from the forward scans of the transfer curves (Figures S12b and S14b), where *V*_G_ was swept at 100 mV/s. The *μ*_h,OECT_ data were calculated from hole transit time measurements at an offset potential of *V*_G_ = *V*_D_ = −0.5 V. The error bars in (c) and (d) were determined by averaging data from six channels with varying channel sizes.

Figure 3a shows the volumetric capacitance as a function of C‐rate of the p(Cxg_3_T2‐TT) and p(Cxg_3_T2‐TT) L‐MW polymers, obtained using the galvanostatic charging/discharging method within the potential range of −0.4 V – 0.5 V vs Ag/AgCl. C‐rate, a concept adapted from battery technology, represents the rate at which a polymer film is charged and discharged (expressed in reciprocal hours, see details in SI Section 1, electrochemical characterisations). We employ C‐rate to assess the charge accumulation dynamics of these polymer films in a simple vertical configuration that is relevant to electrochemical storage applications. However, it should be noted that alternative characterisation methods, such as impedance spectroscopy, may be more appropriate for evaluating the dynamics of these polymer films in other applications, such as OECTs or neuromorphic devices. At low C‐rate (<100 C), p(C2g_3_T2‐TT) and its L‐MW counterpart show a volumetric capacitance of approximately 130 F cm^−3^, consistent with other glycolated thiophene‐based CPs (Table S2).[^5^, ^34^] As the C‐rate was increased to 1000 C, their capacitance decreases by less than 10 %, demonstrating their promising rate capabilities comparable to other p‐type mixed conducting CPs.[^5^, ^6^, ^7^, ^34^] Beyond 1000 C, the two polymers show a rapid drop in capacitance, which can be assigned to the limit of ion mobility in the polymer films and the serial resistance of the ITO glass substrate. The other polymers, p(Cxg_3_T2‐TT) (x = 4, 6, 8) and their L‐MW counterparts, show similar rate capabilities but lower volumetric capacitance, the latter due partly to the reduced doping level and the smaller volumetric density of redox‐active conjugated segments. In addition to the volumetric capacitance, we summarised the volumetric capacities of these polymers in Figure S6 and Table 2.

We probe the hole transport in all polymers in their dry states using top‐gate bottom‐contact organic field‐effect transistors (OFETs). For the p(Cxg_3_T2‐TT) series, the increasing length of alkyl spacers leads to improved OFET hole mobility (*μ*_h,OFET_) (Figure 3b) and on/off ratios (Figure S10). In comparison, although p(Cxg_3_T2‐TT) L‐MW polymers exhibit inferior on/off properties relative to their respective high MW counterparts (Figure S11), they demonstrate comparable *μ*_h,OFET_ values. Therefore, it can be concluded that the incorporation of alkyl spacers can improve the hole transport properties of the p(Cxg_3_T2‐TT) polymers in their dry states, regardless of MW. This improvement could be attributed to the reduced impact of dipoles on the glycol side chains on the electrostatic potential, the fluctuations of which could impede hole transport.[^35^, ^36^, ^37^]

However, OFET hole mobility may not reflect the transport properties of mixed conducting CPs in electrochemical applications, where they function in polar, primarily aqueous, electrolytes.[^38^, ^39^] To capture the CPs’ transport properties in such environments, we fabricated OECTs following a previously reported method and characterised them in 0.1 M NaCl (aq) (see SI for detailed fabrication and characterisation procedures).^[40]^ As summarised in Figure 3c and Table 2, p(C2g_3_T2‐TT) exhibits an exceptional μC* value of 1099 ± 121.0 F cm^−1^ V^−1^ s^−1^, standing as one of the highest reported values among p‐type OECTs. This high value is consistent with a previous report using the same polymers with *M*_n_ ≈ 100 kDa.^[28]^ However, the μC* value declines significantly with increasing alkyl spacer length. This monotonic reduction in μC* values indicates a decrease in mobility and/or volumetric capacitance. In comparison, the μC* values of the p(Cxg_3_T2‐TT) L‐MW (x = 2, 4, 6) are lower than their high MW counterparts, and show a similar downward trend with increasing alkyl spacer length.

**Table 2:** Summary of the charge storage and transport properties of the polymers.

Polymer	*V*_th_^[a]^ (V)	μC* (F cm^−1^ V^−1^ s^−1^)	*μ*_h,OECT_^[b]^ (cm^2^ V^−1^ s^−1^)	Thickness^[c]^ (nm)	*μ*_h,OFET_^[d]^ × 10^−4^ (cm^2^ V^−1^ s^−1^)	Capacity^[e]^ (mAh cm^−3^)
p(**C2**g_3_T2‐TT)	−0.09	1099 ± 121.0	6.60 ± 1.74	203	2.08 ± 0.28	20.9
p(**C4**g_3_T2‐TT)	−0.24	380.3 ± 25.4	1.48 ± 0.15	142	2.80 ± 0.44	15.6
p(**C6**g_3_T2‐TT)	−0.23	267.8 ± 3.1	1.64 ± 0.22	197	10.20 ± 0.32	11.7
p(**C8**g_3_T2‐TT)	−0.28	189.2 ± 21.2	0.98 ± 0.12	145	9.09 ± 0.63	8.1
p(**C2**g_3_T2‐TT) L‐MW	−0.08	524.8 ± 20.6	4.45 ± 0.45	183	3.84 ± 0.75	20.7
p(**C4**g_3_T2‐TT) L‐MW	−0.08	271.8 ± 9.8	3.36 ± 0.43	139	7.27 ± 0.95	17.3
p(**C6**g_3_T2‐TT) L‐MW	−0.19	266.5 ± 1.2	0.91 ± 0.10	103	4.60 ± 0.41	14.4
p(**C8**g_3_T2‐TT) L‐MW	−0.24	426.3 ± 14.1	2.95 ± 0.24	100	13.1 ± 0.81	12.4

[a] The threshold voltage extracted from the $\sqrt{I_{D}}$ vs *V*_G_ plot; [b] The OECT hole mobility extracted from transit time measurements at *V*_G_ = *V*_D_ = −0.5 V. [c] The polymer thickness of the OECTs. [d] The OFET hole mobility extracted from the top‐gate (Al) bottom‐contact (Au) OFET in the saturation regime. [e] The volumetric specific capacity of each polymer measured under the lowest C‐rate during discharging from 0.5 V to −0.4 V.

To understand the decrease in μC* with increasing alkyl spacer length and investigate the impact of alkyl spacers on the hole mobilities of the two polymers series in their electrochemically doped states, we calculated the OECT hole mobilities (*μ*_h,OECT_) from transit time measurements.^[41]^ As summarised in Figure 3d and Table 2, the *μ*_h,OECT_ of p(C4g_3_T2‐TT) is significantly lower than p(C2g_3_T2‐TT) at *V*_G_ = *V*_D_ = −0.5 V, a bias equivalent to 0.5 V vs Ag/AgCl in the CV. This suggests that the reduced *μ*_h,OECT_ is the primary factor contributing to the reduction in μC* for p(C4g_3_T2‐TT). Polymers p(C6g_3_T2‐TT) and p(C8g_3_T2‐TT) exhibit similar *μ*_h,OECT_ values to p(C4g_3_T2‐TT) in contrast to their lower μC* values, indicating that their declined μC* values results from decreased C* values. This attribution is supported by the electrochemical measurements (Figure 3a), which show that p(C6g_3_T2‐TT) and p(C8g_3_T2‐TT) exhibit lower volumetric capacitance than p(C4g_3_T2‐TT). Besides, Chen *et al*. similarly reported that the decreasing C* dominates the lowering in μC* values in polymers with longer alkyl spacers (lower oxygen fractions), whilst the spacer length has a much less significant impact on the *μ*_h,OECT_.^[42]^ In the p(Cxg_3_T2‐TT) L‐MW series, a similar decrease in the *μ*_h,OECT_ is observed from p(C2g_3_T2‐TT) L‐MW to p(C6g_3_T2‐TT) L‐MW, contributing to the decrease in their μC* values.

In summary, in contrast to their positive influence on the *μ*_h,OFET_, incorporating alkyl spacers and increasing MW negatively impact the hole transport of p(Cxg_3_T2‐TT) (x = 4, 6, 8) in polar environments, contradicting observations from previous studies.[^14^, ^15^] The opposite trends in *μ*_h,OFET_ and *μ*_h,OECT_ values with alkyl spacer length suggest the important role of electrolytes in influencing the hole transport. Therefore, we next explore the effect of electrochemical charging on the swelling properties of polymer thin films.

**Figure 4:**
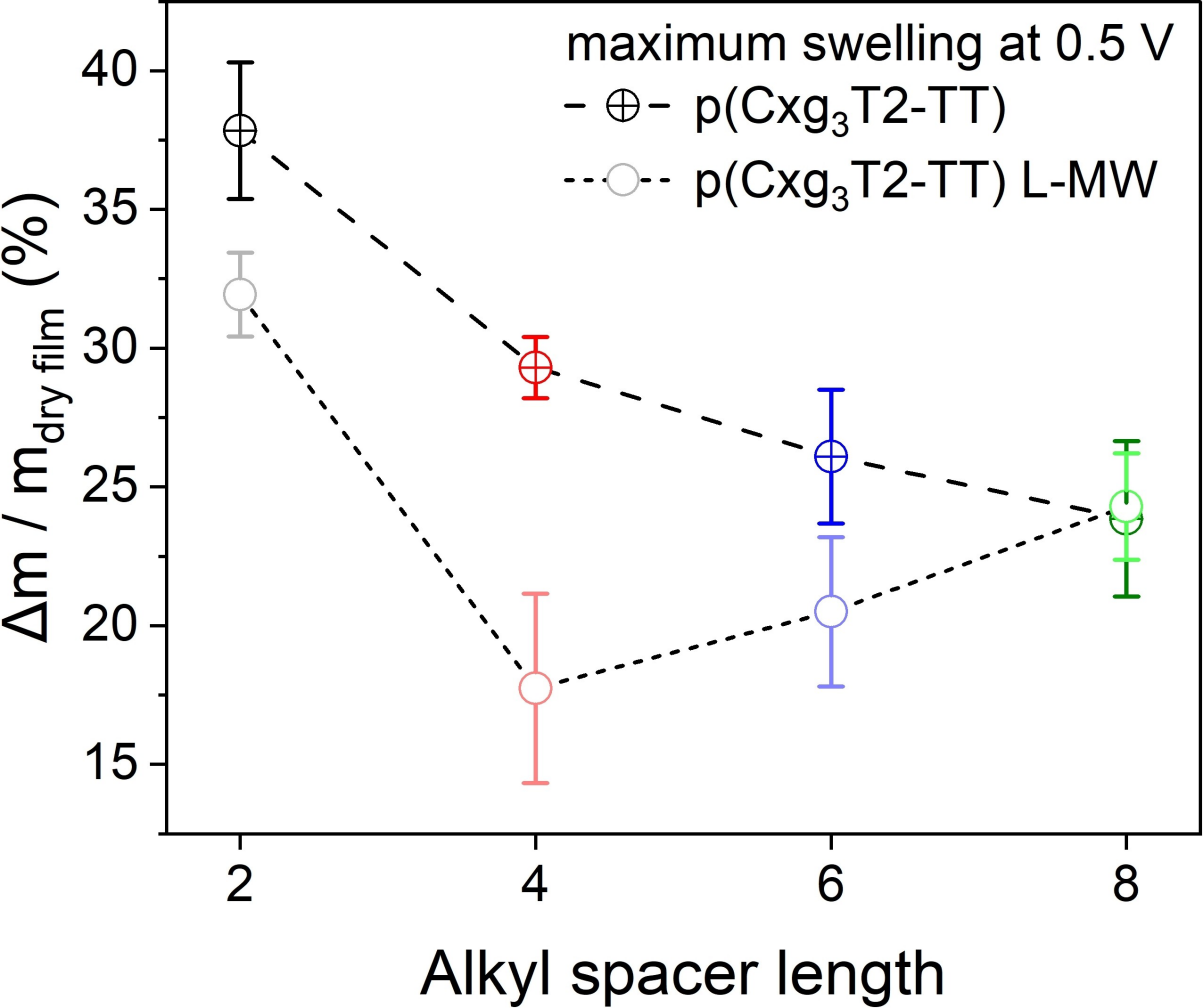
Swelling properties of p(Cxg_3_T2‐TT) and p(Cxg_3_T2‐TT) L‐MW polymers biased at 0.5 V vs Ag/AgCl, measured by EQCM. The swelling is quantified as the fractional change in mass of the swelled thin films with respect to their as‐cast states. Each polymer underwent five consecutive CV cycles, ranging from −0.4 V to 0.5 V vs Ag/AgCl at a scan rate of 10 mV/s. Shown here is the averaged maximum fractional mass change experienced by each polymer film at 0.5 V vs Ag/AgCl during the second to the fifth CV cycle. The error bars represent the averaged swelling data from 5 samples.

To investigate whether swelling—quantified as the fractional change in mass of the swelled thin films with respect to their initial dry pristine states (*Δm*/*m*_dry film_)—directly governs the *μ*_h,OECT_ decline in the p(Cxg_3_T2‐TT) and p(Cxg_3_T2‐TT) L‐MW polymers, we employed electrochemical quartz crystal microbalance (EQCM) to monitor film mass changes during CV measurements. As shown in Figure 4, the swelling of p(Cxg_3_T2‐TT) polymers at 0.5 V vs Ag/AgCl decreases monotonically with increasing alkyl spacer length, reaching 38 %, 29 %, 26 % and 24 % for p(Cxg_3_T2‐TT) (x = 2, 4, 6, 8), respectively. In comparison, p(Cxg_3_T2‐TT) L‐MW polymers tend to show less mass uptake compared with their respective high MW counterparts, consistent with the literature.^[19]^ Interestingly, the mass uptake of p(Cxg_3_T2‐TT) L‐MW polymers does not follow a monotonic downward trend with respect to alkyl spacer length, i.e. p(Cxg_3_T2‐TT) L‐MW (x = 6, 8) swell more than p(C4g_3_T2‐TT) L‐MW. Despite the non‐monotonic swelling trend, both p(Cxg_3_T2‐TT) and p(Cxg_3_T2‐TT) L‐MW series demonstrate that the incorporation of alkyl spacers results in less swelling than the polymers without a spacer.

The observation that alkyl spacers suppress swelling is consistent with a previous study by Maria *et al*. on three NDI‐based n‐type polymers, which showed that a longer alkyl spacer on the NDI units can reduce polymer swelling in 0.1 M NaCl (aq).^[14]^ However, contrary to Maria's study, which showed that reduced swelling led to improved electron mobilities in OECTs, our study exhibited a decline in *μ*_h,OECT_ with reduced swelling in p(Cxg_3_T2‐TT) polymers. This decline, worsening with longer alkyl spacer length and less swelling, aligns with the observation in Ohayon's work, where alkyl spacers on the gT2 units also deteriorated the electron transport.^[12]^ The discrepancy in the improved electron mobility in NDI‐based n‐type polymers in Maria's work and the deteriorated hole mobility in our p‐type p(Cxg_3_T2‐TT) polymers and Ohayon's work suggests that the functionality of alkyl spacer is related to the conjugation unit onto which alkyl spacers are introduced.

**Figure 5:**
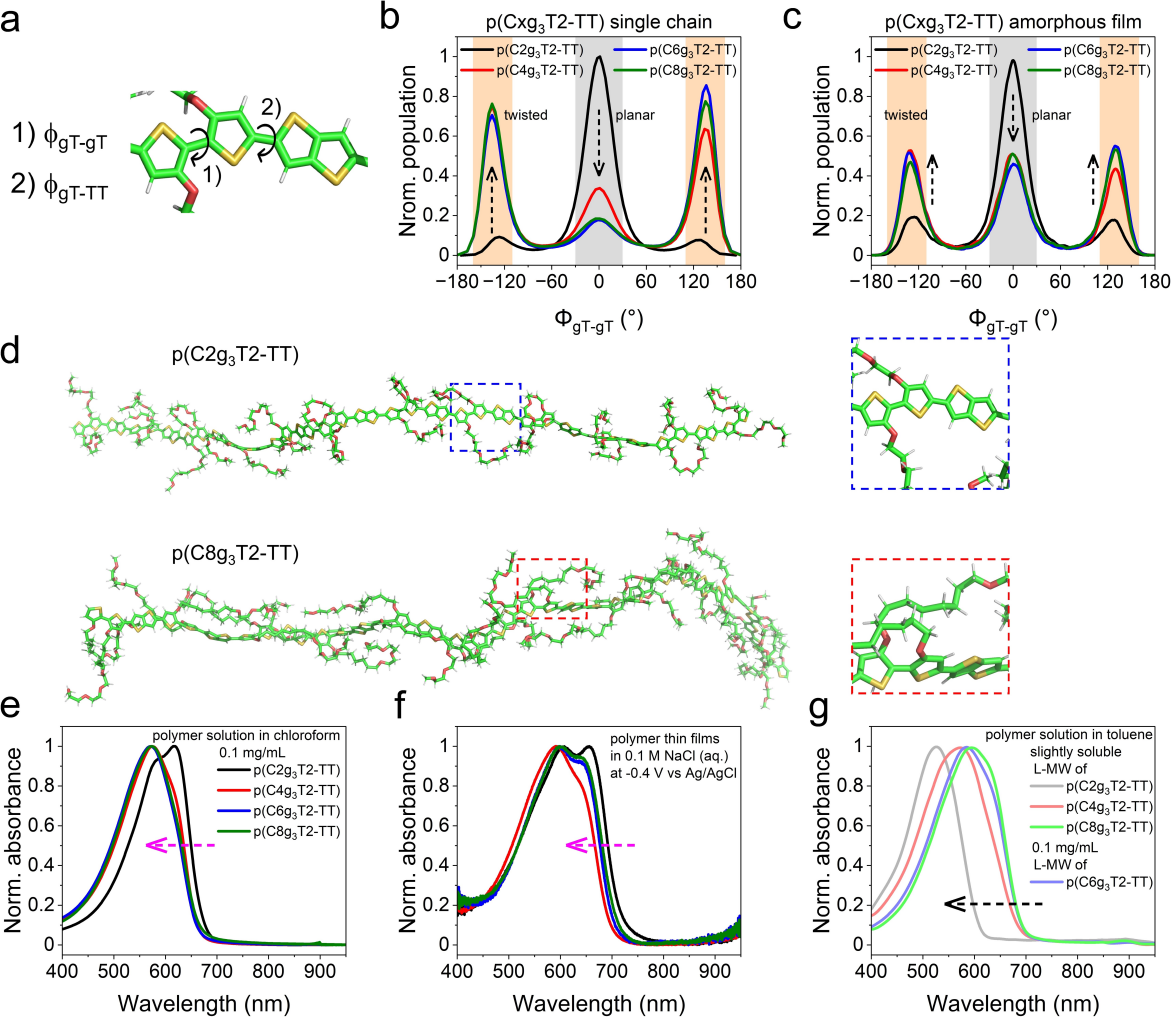
Simulation and experimental evidence for alkyl spacer interaction‐induced backbone twisting. (a) Snapshots, taken from atomistic MD simulations, of the backbone of a p(Cxg_3_T2‐TT) repeat unit, indicating the definition of *φ*_gT‐gT_ and *φ*_gT‐TT_. Colour Scheme: carbon (green), oxygen (red), sulphur (yellow), hydrogen (light grey). (b, c) Normalised *φ*_gT‐gT_ population distribution, extracted from atomistic MD simulations, of (b) p(Cxg_3_T2‐TT) (x = 2, 4, 6, 8) (14 repeat units) single chains and (c) p(Cxg_3_T2‐TT) amorphous films composed of 100 respective single chains in 0.2 M NaCl (aq) solutions. (d) Snapshots, taken from atomistic MD simulations, of p(C2g_3_T2‐TT) and p(C8g_3_T2‐TT) single chains immersed in 0.2 M NaCl (aq) solutions. Water molecules, Na^+^ cations and Cl^−^ anions are hidden for clarity. The enlarged figures in blue and red dashed rectangles beside the polymers illustrate the approximately planar dihedral within the oxy‐bithiophene groups in p(C2g_3_T2‐TT) and the significantly twisted ones in p(C8g_3_T2‐TT), respectively. (e–g) Normalised absorbance spectra of (e) 0.1 mg/mL p(Cxg_3_T2‐TT) solutions in chloroform, (f) p(Cxg_3_T2‐TT) thin films on ITO glass substrates in their neutral states (−0.4 V vs Ag/AgCl in the thin‐film electrochemical measurements) in a 0.1 M NaCl (aq) electrolyte and (g) p(Cxg_3_T2‐TT) L‐MW solutions in toluene at varying concentrations. The polymers p(Cxg_3_T2‐TT) L‐MW (x = 2, 4, 8) are only slightly soluble in toluene, thus their solutions for testing are saturated. (e) and (f) share the same legend. The magenta arrows in (e) and (f) indicate the spectral shift of p(Cxg_3_T2‐TT) (x = 4, 6, 8) relative to p(C2g_3_T2‐TT), whilst the back one in (g) indicates that of p(C2g_3_T2‐TT) L‐MW relative to p(Cxg_3_T2‐TT) L‐MW (x = 4, 6, 8).

One significant difference in the glycol side chain attachment between the polymers studied here and those in literature, where alkyl spacers improve electronic charge carrier mobility, is that in those cases, the glycol chains are attached on opposite sides of a single conjugation unit, e.g. naphthalenediimide (NDI) and diketopyrrolopyrrole (DPP).[^14^, ^43^] In our study, however, they are positioned on two adjacent conjugation units (3,3’‐dialkoxybithiophene) connected by a C−C single bond, which, despite the planarizing effect of the through‐space S—O interaction between the two thiophenes, allows the backbone to twist. A previous MD simulation study showed that the p(gT2) backbone can undergo twisting due to the chelation of one cation by the adjacent two glycol side chains.^[20]^ In our study, the alkyl spacers on the p(Cxg_3_T2‐TT) (x = 4, 6, 8) polymers are non‐polar units, and would not be easily solvated in polar solvents. However, if adjacent alkyl spacers couple together, they could reduce their exposure to the polar solvent, and hence lower the overall free energy of the polymer‐electrolyte system (discussed further later). Such coupling would lead to backbone twisting, reducing both conjugation and linearity of the backbone (as shown in Figure 5d red rectangle and discussed below), and hence to poorer electronic charge transport. It should be noted that similar coupling may also take place between the alkyl spacers and their parent non‐polar backbone.

To test the hypothesis that alkyl spacer interaction‐induced backbone twisting results in inferior hole mobilities in p(Cxg_3_T2‐TT) (x = 4, 6, 8), we conducted atomistic molecular dynamics (MD) simulations on p(Cxg_3_T2‐TT) (x = 2, 4, 6, 8) single chains and amorphous films, with each polymer chain comprising 14 repeat units. Both the single chains and the amorphous films were simulated in 0.2 M NaCl (aq) solution using explicit solvent and solute molecules. Detailed MD simulation methods are summarised in SI section 9. We analysed the dihedral angles between the two glycolated thiophene units (*φ*_gT‐gT_) and between the glycolated thiophene and the thienothiophene (*φ*_gT‐TT_) (Figure 5a). The 0° dihedral conformation is defined as the two thiophene rings in the 3,3’‐dialkoxybithiophene unit being positioned in the same plane, with the two sulphur atoms positioned on different sides of the joint C−C bond. As shown in Figures 5b and S21, the p(C2g_3_T2‐TT) single chain predominately exhibits *φ*_gT‐gT_ around 0° and *φ*_gT‐TT_ around 25°. These small average dihedral angles suggest that the backbone of p(C2g_3_T2‐TT) is approximately planar, with the side chains positioned on either side of the backbone. In contrast, introducing alkyl spacers in p(Cxg_3_T2‐TT) (x = 4, 6, 8) shifts the predominant *φ*_gT‐gT_ to 135°, leaving only a minor population near 0°, whilst the *φ*_gT‐TT_ remains nearly unchanged. The pronounced change in the *φ*_gT‐gT_ indicates substantial torsion within the 3,3’‐dialkoxybithiophene unit, which reduces the free energy of the polymer (Figure S22). Besides, MD snapshots show this torsion is accompanied by the coupling of alkyl spacers within the aqueous environment. Figure 5d illustrates the approximately planar p(C2g_3_T2‐TT) backbone and the twisted p(C8g_3_T2‐TT) backbone. The enlarged section of p(C8g_3_T2‐TT) depicts the coupling of the adjacent alkyl spacers. Moreover, the MD snapshot of p(C8g_3_T2‐TT) also suggests that long alkyl spacers tend to align along the backbone, potentially creating a shielding effect that impedes ions from approaching the backbone, and hence deteriorates the polymer's charge storage properties. In the simulation of p(Cxg_3_T2‐TT) amorphous films, each composed of 100 single chains, the starting backbone conformation of each polymer chain reflects the corresponding single‐chain simulations. As shown in Figures 5c and S21, the film simulation results for *φ*_gT‐gT_ and *φ*_gT‐TT_ variation against increasing alkyl spacer length follow the same trend as those in the single‐chain simulations. In summary, both types of simulations predict that backbone twisting could occur in p(Cxg_3_T2‐TT) (x = 4, 6, 8).

To test the prediction made by the MD simulations, we studied the effect of alkyl spacer length on the UV‐Vis absorption of p(Cxg_3_T2‐TT) polymers in solution. A twisted backbone is expected to limit the π‐conjugation, leading to blue‐shifted absorption spectra compared with those of a planar backbone with the same chemical structure.[^44^, ^45^, ^46^] The absorption spectra of 0.1 mg/mL p(Cxg_3_T2‐TT) polymer solutions in the weakly polar solvent chloroform (*ϵ*_r_ = 4.8) (Figure 5e) show that the absorption onset and peak of p(Cxg_3_T2‐TT) (x = 4, 6, 8) clearly blue‐shift by approximately 10 and 46 nm, respectively, relative to those of p(C2g_3_T2‐TT). For the p(Cxg_3_T2‐TT) (x = 4, 6, 8) thin films processed from chloroform, in their neutral states (−0.4 V vs Ag/AgCl) in 0.1 M NaCl (aq) solution (*ϵ*_r_ close to that of water), their absorption onsets blue‐shift by approximately 18, 10 and 4 nm, respectively, relative to that of p(C2g_3_T2‐TT) thin film at 714 nm (Figure 5f). In addition, the 0–1 vibronic transition peaks of these three polymers are similarly blue‐shifted. The blue‐shifted absorption spectra of both p(Cxg_3_T2‐TT) (x = 4, 6, 8) solutions and thin films suggest that the backbones of p(Cxg_3_T2‐TT) (x = 4, 6, 8) are more twisted than the p(C2g_3_T2‐TT) backbone, corroborating the predictions made by the MD simulations.

To further substantiate the influence of solvent polarity on backbone conformation, we dissolved the p(Cxg_3_T2‐TT) L‐MW polymers in the non‐polar solvent toluene (*ϵ*_r_ = 2.38) and measured their absorption spectra. We chose p(Cxg_3_T2‐TT) L‐MW polymers instead of p(Cxg_3_T2‐TT) polymers because of the insolubility of p(Cxg_3_T2‐TT) (x = 2, 4) in toluene, though p(Cxg_3_T2‐TT) L‐MW (x = 2, 4, 8) are only slightly soluble. In the inherently non‐polar solvent toluene, the non‐polar alkyl spacers and backbones are expected to dissolve well, avoiding backbone twisting, whereas the polar glycol blocks on the side chains may encounter solubility issues. As anticipated, in toluene, the absorption spectra of p(Cxg_3_T2‐TT) L‐MW (x = 4, 6, 8) show no blueshift compared to p(C2g_3_T2‐TT) L‐MW (Figure 5g), in contrast to their behaviour in chloroform. In fact, the absorption spectra of p(C2g_3_T2‐TT) L‐MW now show a blueshift. While this effect could conceivably be explained by backbone twisting to enable glycol group aggregation in toluene, we lack additional evidence for this explanation and therefore other explanations for the blueshift cannot be ruled out. This supplementary experiment provides further evidence that the backbone conformation of p(Cxg_3_T2‐TT) polymers is sensitive to the influence of solvent on amphiphilic side chain interactions.

In this work, we have used p(Cxg_3_T2‐TT) and p(Cxg_3_T2‐TT) L‐MW polymers as representatives to investigate the impact of alkyl spacers and MW on the charge transport properties of oxy‐bithiophene‐based p‐type CPs. This design strategy was based on previous findings that long alkyl spacers and high MW could improve the electronic charge transport properties of mixed conducting CPs.[^14^, ^15^, ^19^] However, our observations contradict some previous findings, suggesting that whilst the alkyl spacers improve hole mobilities in our CPs in OFETs, they diminish the polymers’ charge transport and storage properties in aqueous electrolytes. We attribute this deterioration to the backbone twisting induced by the alkyl spacer coupling in polar environments. This coupling phenomenon can be an intuitive analogue to the formation of micelles, where the hydrophobic domain—comprising alkyl spacers and backbones—rearranges to form the micelle core. This rearrangement reduces the interfacial energy between the hydrophobic domain and the polar solvent, whilst increasing solvent entropy by setting free previously trapped solvent molecules from the solvation shell of the hydrophobic domain. To accommodate this rearrangement, the polymer backbone twists, without interrupting the favourable interactions between glycol chains—acting as the micelle shell—and the polar solvent.

The alkyl spacer coupling‐induced backbone twisting effect is likely to be further amplified by higher MW, as longer polymer chains are more likely to incorporate these twisting defects. This phenomenon is similar to the entanglement commonly observed in long polymer chains.^[24]^ GIWAXS measurements support this hypothesis. Within the p(Cxg_3_T2‐TT) series, where all polymers have long chains, p(C2g_3_T2‐TT) shows more distinct crystalline features than p(Cxg_3_T2‐TT) (x = 4, 6, 8) (Figure S7), and a similar trend is observed within the p(Cxg_3_T2‐TT) L‐MW series (Figure S8). This trend suggests that alkyl spacers disrupt polymer packing. Considering the influence of MW, we find that p(Cxg_3_T2‐TT) L‐MW (x = 4, 6, 8) show significantly more defined GIWAXS patterns than their high MW counterparts, which display minimal scattering patterns. This indicates that the alkyl spacers deteriorate the packing more significantly in p(Cxg_3_T2‐TT) (x = 4, 6, 8) than in their L‐MW counterparts. Although entanglement may broaden GIWAXS patterns, it is unlikely to completely eliminate them.^[24]^ Based on these comparisons, we argue that p(Cxg_3_T2‐TT) with alkyl spacers have more backbone twisting than p(Cxg_3_T2‐TT) L‐MW.

Regarding the improved hole mobilities in OFETs, we hypothesise that although the backbones of p(Cxg_3_T2‐TT) (x = 4, 6, 8) are expected to be twisted, the benefits of alkyl spacers in reducing the impact of dipoles on the glycol side chains on the electrostatic potential—fluctuations of which could impede hole transport—compete with and may outweigh the detrimental effects of the twisted backbone.[^35^, ^36^, ^37^] Besides, since charge carriers in OFETs move in a thickness of a few monolayers of the channel material at the material‐dielectric interface rather than through the bulk of the material, the interface morphology and dielectric property can significantly influence OFET mobility. The presence of the polymer‐dielectric interface is likely to encourage planarization of the polymer chains in that region, even if the polymers were deposited in a relatively polar solvent. Moreover, during the OFET fabrication (see details in SI Section 1, Organic field‐effect transistor (OFET) fabrication and measurements), the polymer layer was annealed at 100 °C both before and after dielectric (CYTOP) deposition. The annealing process, combined with the hydrophobic nature of the CYTOP surface, may induce the polymer chains to realign. Additionally, as reported by Schmode *et al*., the alkyl spacers in their polythiophene‐based mixed conducting CPs seem to enhance the lamellar structure of the film, thereby improving the OFET hole mobility.^[15]^

Our simulation and spectroscopic findings demonstrate that the functionality of alkyl spacers is sensitive to solvating environments (polymer solvents and electrolyte solvents). Enthalpic interactions between the alkyl spacers and the solvating environments can strongly influence the polymer backbone conformation, thereby impacting the electronic properties of the polymer. Such solvent polarity‐controlled polymer conformation has been observed in other CPs featuring polarity mismatches between polymer segments and the solvent. For example, Rakchart *et al*. observed that polymer solvents that are either too polar (alkyl alcohols) or too non‐polar (e.g. aliphatic alkanes) would cause the CP—phenylene vinylene (PPV) featuring alkyl side chains—to collapse, forming a coiled conformation, whereas a good solvent (e.g. chloroform) that matched the polarity of the CP allowed the backbones to adopt an extended conformation.^[44]^ Similarly, Zhihua *et al*. reported that a water‐soluble PPV featuring amphiphilic side chains adopted an extended conformation in chloroform but a coiled conformation in methanol and water.^[47]^ In a recent simulation study by Siemons *et al*., the authors showed that oxy‐bithiophene‐based CPs with mixed glycol/alkyl side chains tended to twist their backbones within the adjacent oxy‐bithiophene units where alkyl side chains are attached.^[29]^ This twisting resulted from the alkyl side chain aggregation and was driven by a significant reduction in free energy associated with the CP chain conformation. Compared to these cases, the polymers in our study likely show polarity mismatches between the alkyl spacers and chloroform/water and between the glycol blocks and toluene. These polarity mismatches drive backbone twisting in order to reduce the free energy.

Although the alkyl spacers in oxy‐bithiophene‐based CPs lead to backbone twisting and the deterioration of electronic properties, this phenomenon holds potential for exploitation. For example, precise adjustments to the density, length and distribution of alkyl spacers provide an opportunity to control the backbone conformation, and hence to control, to some degree, the electronic structure of mixed conducting polymers. Besides, the incorporation of extended alkyl spacers could enhance polymer solubility in non‐polar, non‐chlorinated solvents like toluene, wherein the polymer can maintain a flat backbone conformation. These non‐chlorinated organic solvents are generally more environmentally friendly and less toxic for users compared with chlorinated solvents such as chloroform and chlorobenzene.

## Conclusion

In this work, we investigate the impact of alkyl spacer length and molecular weight on the charge transport and storage properties of oxy‐bithiophene‐based mixed conducting polymers. Contrary to previous studies, we demonstrate that the introduction of alkyl spacers close to the conjugated polymer backbone leads to a reduction in both hole mobility and volumetric capacitance of p(Cxg_3_T2‐TT) polymers in aqueous electrolytes, and high molecular weight can amplify these negative effects. The deterioration in hole mobilities is assigned to the backbone twisting induced by alkyl spacer coupling in polar environments, whilst the decrease in charge storage properties is partly attributed to the reduced volumetric density of redox sites and depth of charging. Our findings highlight the often‐overlooked relationship between the side chain polarity, molecular weight and polymer solvents/device electrolytes in influencing polymer performance. Furthermore, our study underscores the importance of considering the interactions between the polymers and their solvation environment when designing new mixed conducting polymers.

## Experimental Section

The experimental section is available in the Supporting Information of the manuscript.

## Conflict of Interests

The authors declare no conflict of interest.

1

## Supporting information

As a service to our authors and readers, this journal provides supporting information supplied by the authors. Such materials are peer reviewed and may be re‐organized for online delivery, but are not copy‐edited or typeset. Technical support issues arising from supporting information (other than missing files) should be addressed to the authors.

Supporting Information

## Data Availability

The data that support the findings of this study are available from the corresponding author upon reasonable request.
