# Supplementary material for: The Influence of Alkyl Spacers and Molecular Weight on the Charge Transport and Storage Properties of Oxy‐Bithiophene‐Based Conjugated Polymers
Source: Angew Chem Int Ed Engl. 2024 Dec 23;64(6):e202417897. doi: 10.1002/anie.202417897 (PMC11795722; doi:10.1002/anie.202417897)
Supplement: Supplementary file 1 — Supporting Information [file ANIE-64-e202417897-s001.pdf]

Angewandte Chemie
Eine Zeitschrift der Gesellschaft Deutscher Chemiker

Supporting Information

The Influence of Alkyl Spacers and Molecular Weight on the Charge Transport and Storage Properties of Oxy-Bithiophene-Based Conjugated Polymers

*H. Yu, A. Marks, S. M. Tuladhar, N. Siemons, I. Anderson, S. Bidinger, S. T. Keene, T. J. Quill, R. Wu, O. Gough, G. Wu, F. Eisner, A. Salleo, J. Rivnay, G. G. Malliaras, P. R. F. Barnes, I. McCulloch, J. Nelson**

Supporting information

The Influence of Alkyl Spacers and Molecular Weight on the Charge Transport and Storage Properties of Oxy-bithiophene-based Conjugated Polymers

Hang Yu,[a] Adam Marks,[b,c] Sachetan M. Tuladhar,[a] Nicholas Siemons,[a] Iona Anderson,[a] Sophia Bidinger,[d] Scott T. Keene,[d] Tyler J. Quill,[c] Ruiheng Wu,[e] Olivia Gough,[a] Guanchen Wu,[a] Flurin Eisner,[a] Alberto Salleo,[c] Jonathan Rivnay,[e] George G. Malliaras,[d] Piers R. F. Barnes,[a] Iain McCulloch,[b,f] and Jenny Nelson*[a]

[a] H. Yu, S. M. Tuladhar, N. Siemons, I. Anderson, O. Gough, G. Wu, F. Eisner, P. R. F. Barnes, J. Nelson
Department of Physics and Centre for Processable Electronics
Imperial College London
London SW7 2AZ, United Kingdom
Email: jenny.nelson@imperial.ac.uk

[b] A. Marks, I. McCulloch
Department of Chemistry
University of Oxford
Oxford OX1 2JD, United Kingdom

[c] A. Marks, T. J. Quill, A. Salleo
Department of Materials Science and Engineering
Stanford University
Stanford CA 94305, United States

[d] S. Bidinger, S. T. Keene, G. G. Malliaras
Department of Engineering
University of Cambridge
Cambridge CB3 0FA, United Kingdom

[e] R. Wu, J. Rivnay
Department of Biomedical Engineering
Northwestern University
Evanston IL 60208, United States

[f] I. McCulloch
Andlinger Center for Energy and the Environment, and Department of Electrical and Computer Engineering
Princeton University
Princeton NJ 08544, USA

Table of Contents

1. Experimental section 1

2. Solution UV-Vis measurements and properties of p(Cxg_3T2-T) 6

3. Electrochemical and spectroelectrochemical measurements of polymer thin films 7

3.1. CV measurements in aqueous electrolytes 7

3.2. Spectroelectrochemical measurements in aqueous electrolytes 8

3.2.1. p(Cxg_3T2-TT) 8

3.2.2. p(Cxg_3T2-TT) L-MW 8

3.2.3. p(Cxg_3T2-T) 9

3.3. Charge storage properties and comparison 9

4. Grazing-Incidence Wide-Angle X-ray Scattering (GIWAXS) of polymer thin films 11

4.1. p(Cxg_3T2-TT) 11

4.2. p(Cxg_3T2-TT) L-MW 12

4.3. p(Cxg_3T2-T) 12

5. Organic field effect transistor (OFET) measurements 13

5.1. p(Cxg_3T2-TT) 13

5.2. p(Cxg_3T2-TT) L-MW 14

6. Organic electrochemical transistor (OECT) measurements 15

6.1. p(Cxg_3T2-TT) 15

6.2. p(Cxg_3T2-TT) L-MW 17

7. Electrochemical quartz crystal microbalance (EQCM) measurements 19

7.1. p(Cxg_3T2-TT) 19

7.2. p(Cxg_3T2-TT) L-MW 20

8. Quantum chemical simulations 21

8.1. Methods 21

8.2. Results 22

8.2.1. $(gT2\text{-}TT)_2$ and $(gT2\text{-}T)_2$ at different oxidised states 22

8.2.2. $(gT\text{-}TT)_3$ with flat and twisted backbone 24

9. Molecular dynamics simulation 26

10. Materials synthesis and characterisations 28

10.1. Synthesis of 13-(thiophen-3-yloxy)-2,5,8,11-tetraoxatridecane 28

10.2. Synthesis of 3,3'-bis((2,5,8,11-tetraoxatridecan-13-yl)oxy)-2,2'-bithiophene 29

10.3. Synthesis of 13,13'-((5,5'-dibromo-[2,2'-bithiophene]-3,3'-diyl)bis(oxy))bis(2,5,8,11-tetraoxatridecane) 31

10.4. Synthesis of Polymer p($C2g_3$T2-TT) 33

10.5. Synthesis of Polymer p($C2g_3$T2-T) 35

10.6. Synthesis of 4-(thiophen-3-yloxy)butan-1-ol 37
10.7. Synthesis of 4-(thiophen-3-yloxy)butyl 4-methylbenzenesulfonate 39
10.8. Synthesis of 15-(thiophen-3-yloxy)-2,5,8,11-tetraoxapentadecane 40
10.9. Synthesis of 15-((2-bromothiophen-3-yl)oxy)-2,5,8,11-tetraoxapentadecane 42
10.10. Synthesis of 3,3'-bis((2,5,8,11-tetraoxapentadecan-15-yl)oxy)-2,2'-bithiophene 43
10.11. Synthesis of 15,15'-((5,5'-dibromo-[2,2'-bithiophene]-3,3'-diyl)bis(oxy))bis(2,5,8,11-tetraoxapentadecane) 45
10.12. Synthesis of Polymer p(C4g_3T2-TT) 47
10.13. Synthesis of Polymer p(C4g_3T2-T) 49
10.14. Synthesis of 6-(thiophen-3-yloxy)hexan-1-ol 51
10.15. Synthesis of 6-(thiophen-3-yloxy)hexyl 4-methylbenzenesulfonate 52
10.16. Synthesis of 17-(thiophen-3-yloxy)-2,5,8,11-tetraoxaheptadecane 53
10.17. Synthesis of 17-((2-bromothiophen-3-yl)oxy)-2,5,8,11-tetraoxaheptadecane 55
10.18. Synthesis of 3,3'-bis((2,5,8,11-tetraoxaheptadecan-17-yl)oxy)-2,2'-bithiophene 57
10.19. Synthesis of 17,17'-((5,5'-dibromo-[2,2'-bithiophene]-3,3'-diyl)bis(oxy))bis(2,5,8,11-tetraoxaheptadecane) 58
10.20. Synthesis of Polymer p(C6g_3T2-TT) 59
10.21. Synthesis of Polymer p(C6g_3T2-T) 62
10.22. Synthesis of 8-(thiophen-3-yloxy)octan-1-ol 63
10.23. Synthesis of 8-(thiophen-3-yloxy)octyl 4-methylbenzenesulfonate 65
10.24. Synthesis of 19-(thiophen-3-yloxy)-2,5,8,11-tetraoxanonadecane 66
10.25. Synthesis of 19-((2-bromothiophen-3-yl)oxy)-2,5,8,11-tetraoxanonadecane 68
10.26. Synthesis of 3,3'-bis((2,5,8,11-tetraoxanonadecan-19-yl)oxy)-2,2'-bithiophene 70
10.27. Synthesis of 19,19'-((5,5'-dibromo-[2,2'-bithiophene]-3,3'-diyl)bis(oxy))bis(2,5,8,11-tetraoxanonadecane) 72
10.28. Synthesis of Polymer p(C8g_3T2-TT) 74
10.29. Synthesis of Polymer p(C8g_3T2-T) 75
References 76

1. Experimental section

Electrochemical characterisation:

The preparation of samples for electrochemical and spectroelectrochemical measurements was performed using the following procedure. Indium-doped tin oxide (ITO) glass substrates (25 × 12 × 1.1 mm) were cleaned by ultrasonication in acetone, deionised water (DIW) and propan-2-ol (IPA) for 10 min, sequentially, and dried by N_2 flow for 30 s. The polymers were dissolved in chloroform (concentration: 10 mg/mL) and coated from the solution onto the cleaned ITO glass substrates via blade coating in an ambient environment. Acetone-wetted swabs were used to define the polymer thin film area of about 14 × 9 mm by wiping off the excess films. The thin films' length, width and thickness were measured by taking an average of at least 6 scans on each pristine film using a profilometer (Bruker DektakXT). The thickness of all films was measured to be within the range of 70 ~ 160 nm.

Cyclic voltammetry (CV) characterisation of polymer thin films was performed using a potentiostat (Ivium CompactStat.h10800) in a custom-made three-electrode cell, i.e. a quartz cuvette with transparent front and back windows. The polymer-coated ITO glass served as the working electrodes (WE), alongside an Ag/AgCl (3M NaCl (aq.)) reference electrode (RE) and a platinum mesh (35 × 25 mm) counter electrode (CE). The electrolyte, 0.1 M NaCl (aq.) solution, was flushed with argon for 20 min to remove the dissolved O_2 molecules before starting a measurement. Argon was continuously injected into the cuvette (above the liquid level) to maintain an inert atmosphere during measurements. All the CV characterisations were performed at a scan rate of 10 mV/s unless otherwise stated.

Galvanostatic charging/discharging (GCD) measurements were performed by applying a constant charging/discharging current of the same magnitude to the samples in the same setup as the abovementioned. The potentiostat monitored the potential change on the WE during the measurements and limited the potential within the range of -0.4 V ~ 0.5 V vs RE. Three consecutive charging/discharging cycles were performed for one current intensity and the second discharging process was used to calculate the specific capacity and C-rate illustrated in Figure 3a via:

$$C = \frac{\int_0^t I\,dt}{V_{film}} \tag{1}$$

and

$$C\text{-}rate = \frac{1}{t} \tag{2}$$

where C is the volumetric specific capacity of a polymer thin film, t is the second discharging time in units of hours, I is the applied current and V_{film} is the volume of the polymer thin film. The current was applied from low to high intensities to evaluate the rate capability of the WE, where a higher current

density led to a shorter charging/discharging time and thus a higher C-rate. The specific capacitance at each C-rate was extracted from the slope of the specific capacity-potential curves above the oxidation onset potential.

Spectroelectrochemical characterisation:

Operando UV-Vis measurements were carried out simultaneously with CV measurements using a UV-Vis spectrometer (OceanOptics Flame-S-VIS-NIR Spectrometer Assembly, 350-1000 nm). The spectrometer collected the transmitted light through the cuvette, electrolyte and the samples (WE) from a tungsten lamp (OceanOptics HL-2000-FHSA) as the light source. A MATLAB code was used to control the spectrometer and process the data.

Electrochemical quartz crystal microbalance (EQCM):

Electrogravimetric measurements were carried out on a quartz crystal microbalance (QCM200, Stanford Research System) interfaced with a potentiostat (Metrohm Autolab PGSTAT302N) to apply electrochemical bias. The substrates, 5 MHz AT-cut gold-coated quartz crystals, were ultrasonically cleaned in acetone, DIW and IPA sequentially for 10 min each and dried by N_2 flow for 30 s. The cleaned substrates were loaded into the QCM sample holder and the absolute frequencies of each substrate (F_0) were read. Polymers were then spin-coated onto the substrates (dynamic spin coating, 2000 rpm for 60 s) from 10 mg/mL polymer solutions in chloroform, and any excess material outside of the gold-coated area on the substrate was wiped off using acetone-wetted swabs. The polymer-coated crystals (samples) were again loaded into the QCM and the absolute frequencies of each sample (F_1) were recorded, whereby the frequency change due to the loaded polymer thin films (Δf_1) can be calculated via $\Delta f_1 = F_1 - F_0$.

A three-electrode cell filled with degassed NaCl (aq.) electrolyte was constructed to measure the active swelling of the polymer thin films during electrochemical processes. The cell consisted of the QCM holder with a sample as the WE, an Ag/AgCl (3M NaCl (aq.)) as the RE and a platinum wire as the CE. During a measurement, the potentiostat was used to control the applied potential, recorded the current signals and simultaneously received the external potential change (ΔV) fed by the QCM. The QCM monitored the frequency change of the samples (Δf_2), which is due to the swelling of the polymer thin film during an electrochemical process, and converted it into ΔV at a pre-set scaling factor, 200 Hz/V. The change in frequency (Δf_1 and Δf_2) can be converted into the change of mass using the Sauerbrey equation, assuming a rigid film. Here, we normalised the active swelling using:

$$Swelling\% = \frac{\Delta f_2}{\Delta f_1} \times 100\% = \frac{-C_f \times \Delta m}{-C_f \times m_{dry\,film}} \times 100\% = \frac{\Delta m}{m_{dry\,film}} \times 100\% \quad (3)$$

where C_f is the sensitivity factor for the quartz crystal substrate used, Δm is the mass change of the thin film during electrochemical processes and $m_{dry\ film}$ is the mass of the dry pristine film on the substrate.

Photoelectron spectroscopy in air (PESA):

PESA measurements were performed using an air photoemission system (APS02, KP Technology) to test the ionisation potential of the pristine polymer thin films. Samples were chosen from the same batch as the electrochemical measurements.

Ultraviolet-Visible-Near-infrared (UV-Vis-NIR) absorption measurements:

For solution absorption measurements, all polymer solutions with a concentration of 0.1 mg/mL in chloroform were contained in a pre-cleaned quartz cuvette (Hellma QS High Precision Cell). The transmittance of the solutions (T_{sol}) in the range of 220 nm ~ 1400 nm was measured using a Shimadzu UV-2600 spectrophotometer. The absorbance of the solutions (A_{sol}) was calculated from the transmittance by comparing T_{sol}, first, with the transmittance of the cleaned cuvette filled with chloroform (T_{ref}) and, second, with the transmittance of the air (T_{air}), using the following equation:

$$A_{sol} = -log\left(\frac{T_{sol}/T_{ref}}{T_{air}/100}\right) \quad (4)$$

The absorption onsets of the resulting spectra were extracted by linear fitting the linear regime of the absorption peaks on the longer wavelength side. The wavelength at which the fitted curve intersects the corresponding spectrum baseline was identified as the absorption onset.

Grazing-Incidence Wide-Angle X-ray Scattering (GIWAXS):

Grazing incidence wide-angle x-ray scattering (GIWAXS) measurements of the p(Cxg_3T2-TT) series were conducted at Stanford Synchrotron Radiation Lightsource, SLAC National Accelerator Laboratory, Beamline 11-3. An incident x-ray energy of 12.7 keV was used. The incidence angle α of the x-ray beam was 0.1°. This angle is below the critical angle of the silicon substrate, such that complete external reflection occurs from the silicon, but the angle is below that of the polymer film, such that the beam probes the entire thickness of the polymer film. Scattering intensity was detected with a Rayonix MX225 CCD 2D area detector. The sample-to-detector distance was calibrated with a LaB_6 polycrystalline standard. All data is reported in terms of the scattering vector, $q = (4\pi/\lambda)\sin(\theta)$, where θ is the angle of scattering. GIWAXS raw data was corrected for geometric distortions introduced

by the flat area detector with Nika 1D SAXS and WAXStools software packages in Igor Pro.[1,2] Polymer thin films were prepared by spin coating onto silicon substrates with native oxide.

Grazing incidence wide-angle X-ray scattering (GIWAXS) of the p(Cxg$_3$T2-TT) L-MW and the p(Cxg$_3$T2-T) series was conducted at the Advanced Photon Source (APS) at Argonne National Laboratory using an incident beam energy of 10.92 keV and an incident angle of 0.14°. Raw scattering data were processed using GIXSGUI and analysed using a custom MATLAB script.[3]

Organic field effect transistor (OFET) fabrication and measurements:

OFETs were fabricated on glass substrates using a top-gate bottom-contact configuration. Detailed procedures are as follows. The glass substrates (20 × 20 × 1.1 mm) were cleaned by successive ultrasonication in 5% Decon 90, DIW, acetone and IPA for 10 min each and dried by N_2 flow. The gold source/drain (S/D) contact electrodes (~ 40 nm) were thermally evaporated atop the glass substrates under a high vacuum (2 ~ 5 × 10^{-6} mbar). The resultant S/D electrodes were first immersed in 2,3,4,5,6-Pentafluorothiophenol (Sigma-Aldrich) solution (1:1000 in IPA) for 20 min to lower the working function of the gold and then washed by IPA. Polymer layers were dynamically spin-coated from 10 mg/mL solution in chlorobenzene onto the S/D contact electrodes at 3000 rpm for 60 s and subsequently heated at 100 °C for 30 min to remove the solvent. Then, the dielectric, CYTOP (AGC Chemicals), was spin-coated from the as-received solvated form onto the polymer layers at 2000 rpm for 60 s and again heated at 100 °C for 30 min. Lastly, the aluminium gate (G) electrodes (~ 60 nm) were thermally evaporated atop the CYTOP under the same conditions as the S/D evaporation. All the above procedures, except for the cleaning of the substrates and the chemical treatment of S/D contact electrodes, were carried out in a glovebox.

OFET devices were characterised using an Agilent B2902A dual-channel Precision Source/Measure Unit (SMU) at room temperature in an N_2 atmosphere (glovebox). Transfer and output features of the devices were measured and the hole mobilities in the saturation regime (μ_{sat}) of each device were extracted from the transfer characters using the following equation[4]:

$$\mu_{sat} = \frac{2L}{C_{diel}W}\left(\frac{\partial\sqrt{I_D}}{\partial V_G}\right)^2 \tag{5}$$

where L is the channel length, W the channel width, C_{diel} the capacitance per unit area of the gate dielectric, I_D the drain current and V_G the gate voltage. In this study, all devices used L = 30, 40 or 50 μm and W = 1000 μm. For a top-gate staggered OFET with a CYTOP dielectric, C_{diel} = 2.1 nF cm^{-2}.

<u>Organic electrochemical transistor (OECT) fabrication and measurements:</u>

OECT substrates were microfabricated based on a previously published protocol for the parylene peel-off technique.[5] On glass wafer substrates, electrodes and interconnects were defined using AZnLOF 2035 (Microchemicals GmbH) negative photoresist. Following this first lithography step, the substrates were activated using oxygen plasma (Diener Electronic Femto) for 1 min for metal adhesion. 5 nm of titanium and 100 nm of gold were then deposited on the substrates in an e-beam evaporator (Kurt J Lesker PVD-75). The gold-coated substrates were placed in acetone for gold liftoff and rinsed with IPA. To ensure adhesion of the insulating parylene-C (PaC) to the glass substrates, the substrates were treated with a 3-(trimethoxysilyl)propyl methacrylate (Silane A174, Thermo Fisher Scientific) solution before PaC deposition. Two layers of 2 µm PaC were deposited with a spin-coated layer of 2% micro-90 solution in between as an anti-adhesive. The resulting substrates entered the second photolithography step to finish the OECT substrate fabrication to finish the OECT substrate fabrication. In this step, the OECT electrode, contact pad, and channel areas were defined using AZ 10XT (Microchemicals GmbH). These areas were formed by reactive ion etching (Oxford 80 Plasmalab plus).

The completed OECT substrates were cleaned by successive ultrasonication in acetone, DIW and IPA for 5 min each and dried by N_2 flow. Before coating with polymers, the substrates were activated using oxygen plasma (Emitech K1050X Oxygen plasma asher) at 80 W for 3 min. Polymers were dynamically spin-coated from 10mg/mL solution in chloroform atop the OECT substrates at 2000 rpm for 60 s. Excessive polymer thin films outside the channel areas were removed via peeling off the sacrificial PaC layer (the top PaC layer) before OECT characterisations.

OECT devices were characterised in an ambient environment using an Agilent B2902A dual-channel SMU controlled by a custom-written Python code. An Ag/AgCl pellet (diameter D = 2 mm and height H = 2 mm) was used as the gate electrode for all measurements. The pellet was immersed in 300 µL 0.1 M NaCl (aq.) electrolyte contained in a PDMS well on top of the OECT devices. Transfer curves of the devices were measured within a gate voltage (V_G) range of 0.2 V to -0.5 V at a scan rate of 100 mV/s and under a constant drain voltage (V_D) of -0.5 V. Output curves were measured within a V_D range of 0 V to -0.5 V at a scan rate of 100 mV/s and under a certain V_G varied from 0.2 V to -0.5 V increasing at the interval of -0.1 V. The hole mobility was determined by a series of AC measurements, in which a sinusoidal voltage sweep with an amplitude of 10 mV (ΔV_G) atop an offset V_G of -0.5 V at varying frequencies (1 Hz to 10 kHz) was applied to the gate and a constant V_D of -0.5 V was applied to the drain. The resulting gate (I_G) and drain current (I_D) at each frequency were collected and analysed by the Python code to determine the gate (ΔI_G) and drain current amplitude (ΔI_D), whereby the frequency-dependent transconductance (g_m, $\Delta I_D/\Delta V_G$) and impedance ($|Z|$, $\Delta I_G/\Delta V_G$) can be extracted. The frequency (f) domain relation between the ΔI_G and ΔI_D is:[6]

$$\Delta I_G(f) = 2\pi f \tau_e \Delta I_D(f) \qquad (6)$$

By linear fitting the $\Delta I_G(f)$ vs $\Delta I_D(f)$ curve, the hole transit time τ_e can be extracted. Using τ_e, channel length (L) and the applied V_D, the hole mobilities of the OECTs ($\mu_{h,OECT}$)can be calculated:

$$\mu_{h,OECT} = \frac{L^2}{\tau_e V_D} \qquad (7)$$

2. Solution UV-Vis measurements and properties of p(Cxg$_3$T2-T)

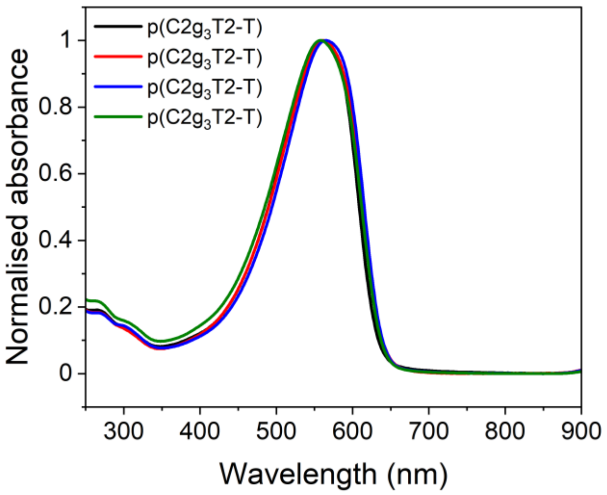

Figure S1. Normalised UV-Vis absorption spectra of 0.1mg/mL p(Cxg3T2-T) polymer solutions in chloroform.

Table S1. Summary of the polymers' properties

Polymer	M_n[a] (kDa) [$Đ$]	IP[b] (eV)	$E_{ox,onset}$[c] (V)	λ_{onset}[d] (nm)
p(**C2**g$_3$T2-T)	51.1 [2.15]	4.45	-0.06 (-0.06)	632
p(**C4**g$_3$T2-T)	19.9 [2.20]	4.51	-0.10 (-0.10)	638
p(**C6**g$_3$T2-T)	28.0 [2.11]	4.56	-0.06 (-0.06)	640
p(**C8**g$_3$T2-T)	13.3 [2.26]	4.51	0.12 (0.12)	637

[a] GPC measurements were carried out using low-D (<1.10) polystyrene standards and DMF as the eluent at 40 °C. [b] Ionisation potential (IP) measured by photoelectron spectroscopy in air (PESA). [c] Oxidation onset potential of the polymer thin films vs Ag/AgCl in their first (and second in parentheses) CV scans (10 mV/s) in Ar-saturated 0.1 M NaCl (aq.) solutions. [d] Absorption onset of 0.1 mg/mL polymer solutions in chloroform.

3. Electrochemical and spectroelectrochemical measurements of polymer thin films

3.1. CV measurements in aqueous electrolytes

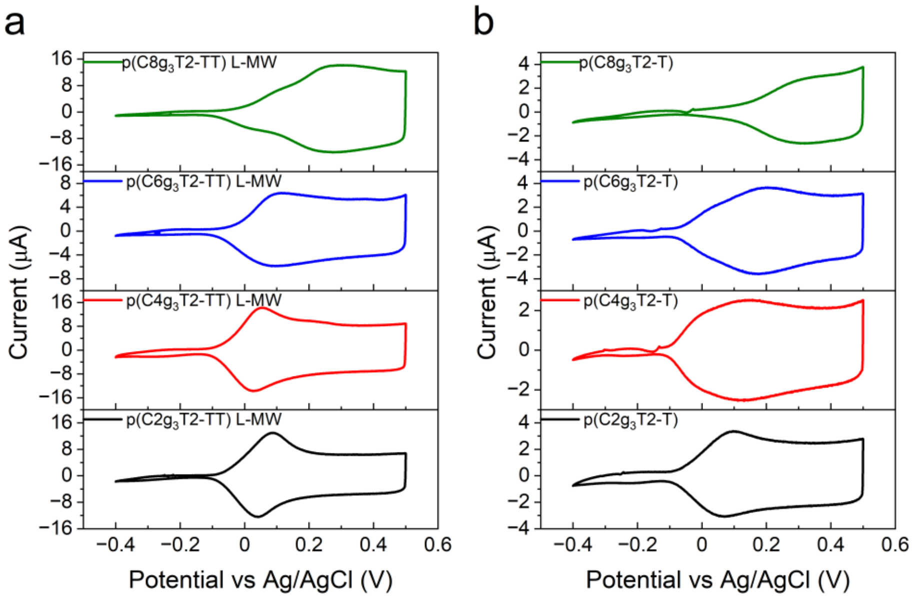

Figure S2. CV measurements of polymer thin films on ITO glass substrates in Ar-saturated 0.1 M NaCl (aq.) with a scan rate of 10 mV/s for (a) p(Cxg3T2-TT) L-MW series and 5 mV/s for (b) p(Cxg3T2-T) series.

3.2. Spectroelectrochemical measurements in aqueous electrolytes

3.2.1. p(Cxg_3T2-TT)

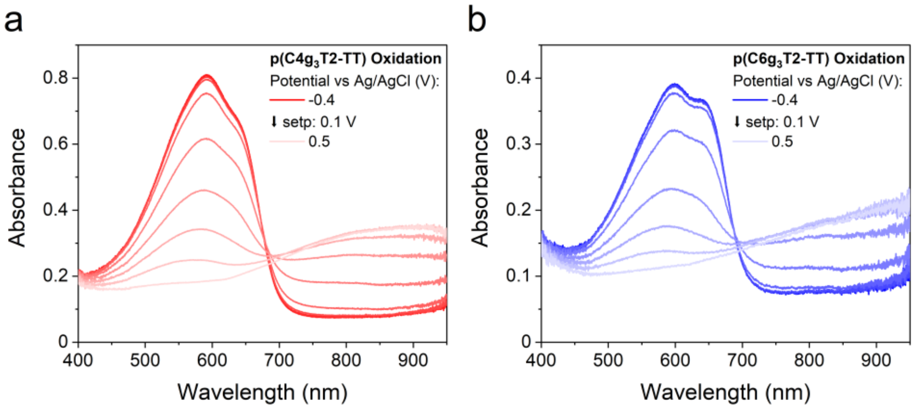

Figure S3. Changes of the absorbance spectra of (a) p(C4g_3T2-TT) and (b) p(C6g_3T2-TT) polymer thin films on ITO glass substrates during the second CV oxidation process. The potential was scanned from -0.4 V to 0.5 V vs Ag/AgCl with a scan rate of 10 mV/s.

3.2.2. p(Cxg_3T2-TT) L-MW

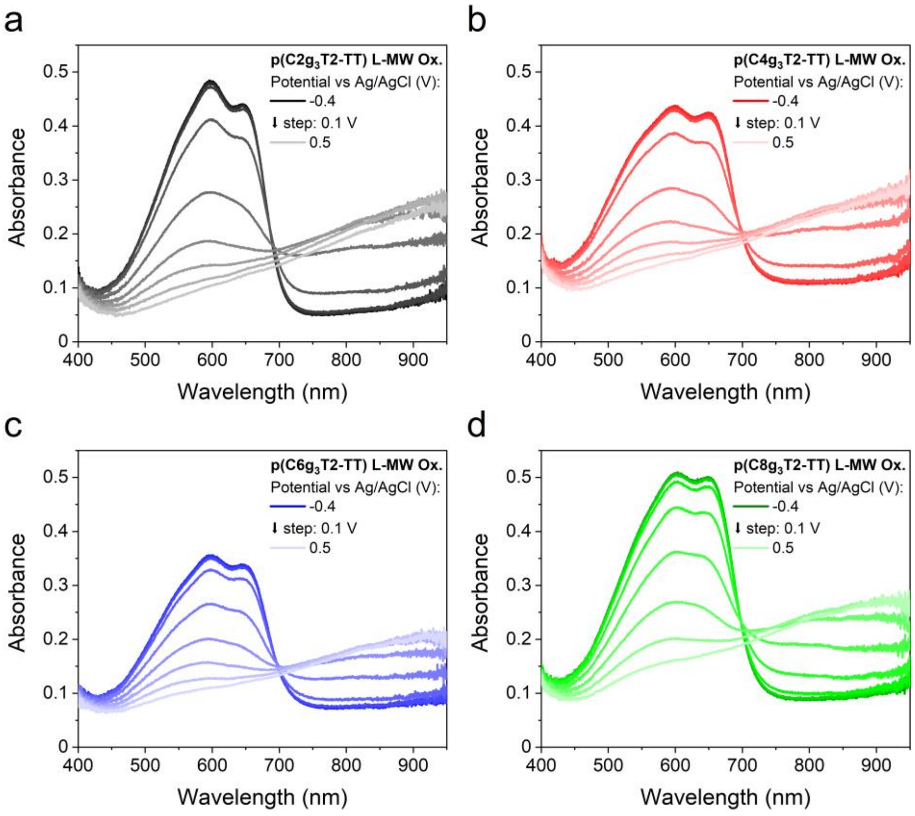

Figure S4. Changes of the absorbance spectra of (a) p(C2g_3T2-TT) L-MW; (b) p(C4g_3T2-TT) L-MW; (c) p(C6g_3T2-TT) L-MW and (d) p(C8g_3T2-TT) L-MW polymer thin films on ITO glass substrates during the second CV oxidation process. The potential was scanned from -0.4 V to 0.5 V vs Ag/AgCl with a scan rate of 10 mV/s.

3.2.3. p(Cxg$_3$T2-T)

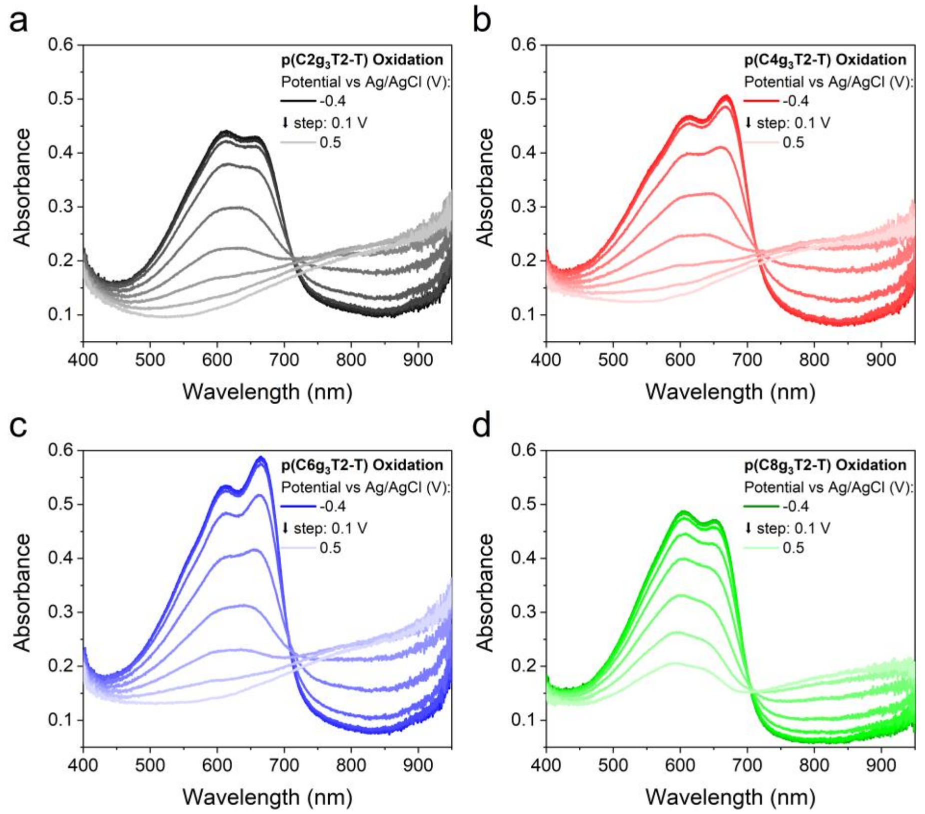

Figure S5. Changes of the absorbance spectra of (a) p(C2g$_3$T2-T); (b) p(C4g$_3$T2-T); (c) p(C6g$_3$T2-T) and (d) p(C8g$_3$T2-T) polymer thin films on ITO glass substrates during the second CV oxidation process. The potential was scanned from -0.4 V to 0.5 V vs Ag/AgCl with a scan rate of 5 mV/s.

3.3. Charge storage properties and comparison

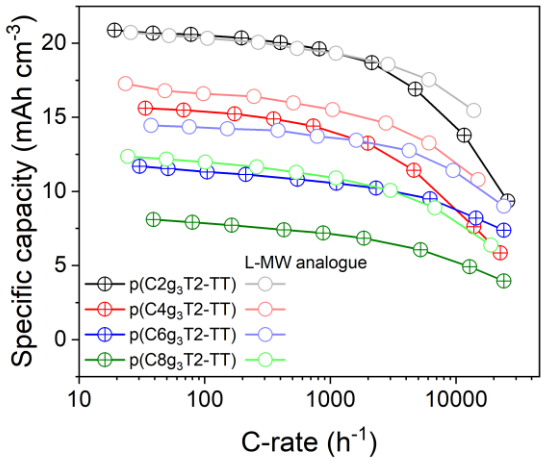

Figure S6. Volumetric specific capacity as a function of C-rate for the p(Cxg$_3$T2-TT) (heavy symbols + lines) and p(Cxg$_3$T2-TT) L-MW (light symbols + lines) polymers.

Table S2. Comparison of volumetric capacitance values between this work and literature

Polymer	Volumetric capacitance (F cm^{-3})	References in the main text
p(**C2**g_3T2-TT)	134.2	This work
p(**C4**g_3T2-TT)	106.0	
p(**C6**g_3T2-TT)	87.7	
p(**C8**g_3T2-TT)	66.3	
P3MEET	80 ± 9	[15]
P3MEEMT	160 ± 12	[15]
P3MEEET	242 ± 17	[15]
p(g3T2)	156 ± 1	[11]
p(g2T2-g4T2)	187 ± 8	[11]
p(g1T2-g5T2)	133 ± 3	[11]
p(g0T2-g6T2)	74 ± 4	[11]
p(g2T-TT)	171.4 ± 8.41	[13]
p(p2T-TT)	103.21 ± 8.17	[13]
p(b2T-TT)	113.93 ± 5.07	[13]
P3APPT	152.0 ± 21.1	[42]
P3AAPT	167.2 ± 7.7	[42]
P3PAAT	84.0 ± 5.9	[42]

4. Grazing-Incidence Wide-Angle X-ray Scattering (GIWAXS) of polymer thin films

4.1. p(Cxg$_3$T2-TT)

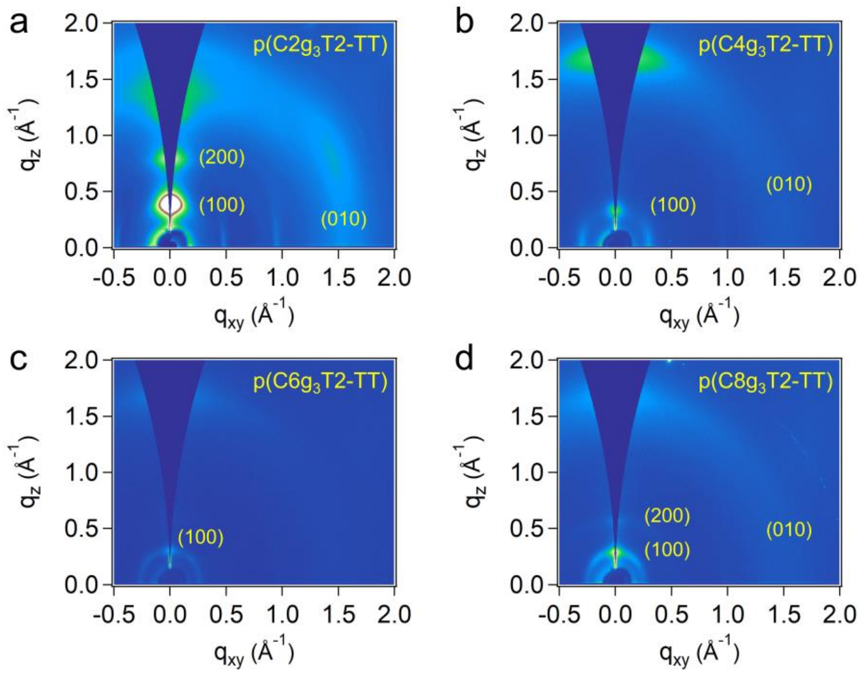

Figure S7. 2D GIWAXS patterns of (a) p(C2g$_3$T2-TT); (b) p(C4g$_3$T2-TT); (c) p(C6g$_3$T2-TT) and (d) p(C8g$_3$T2-TT) polymer thin films in their dry, as-cast condition.

4.2. p(Cxg$_3$T2-TT) L-MW

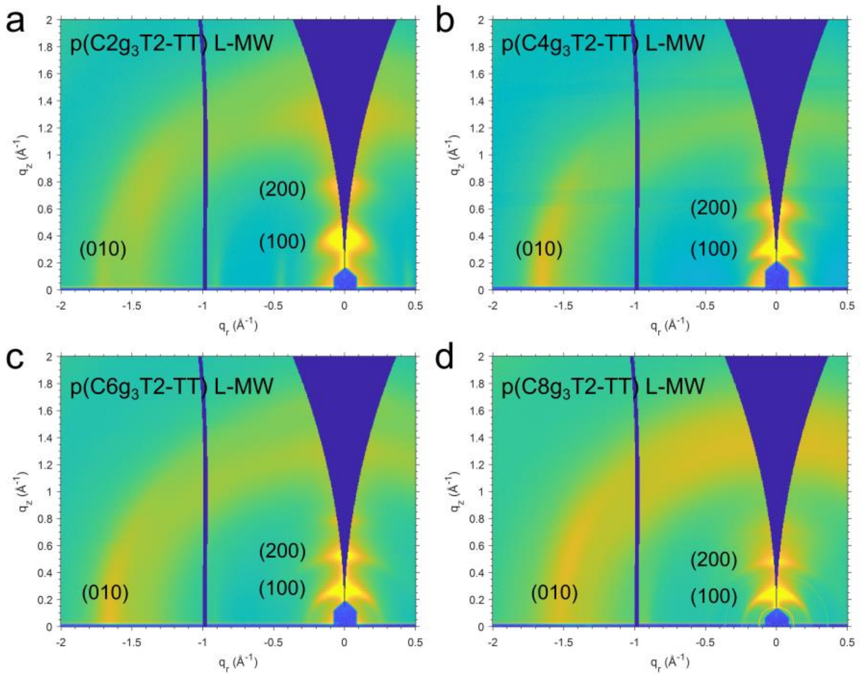

Figure S8. 2D GIWAXS patterns of (a) p(C2g$_3$T2-TT) L-MW; (b) p(C4g$_3$T2-TT) L-MW; (c) p(C6g$_3$T2-TT) L-MW and (d) p(C8g$_3$T2-TT) L-MW polymer thin films in their dry, as-cast condition.

4.3. p(Cxg$_3$T2-T)

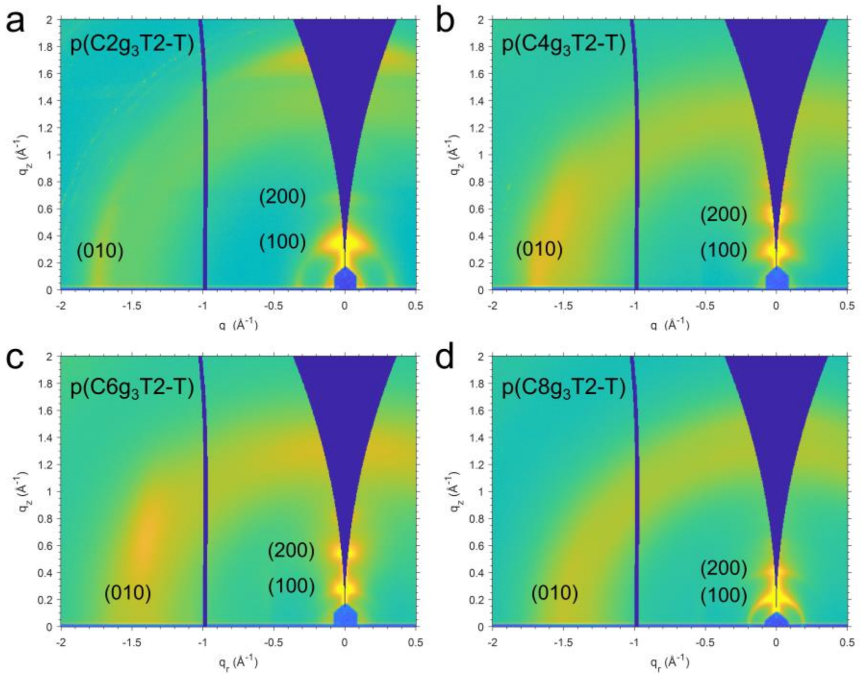

Figure S9. 2D GIWAXS patterns of (a) p(C2g$_3$T2-T); (b) p(C4g$_3$T2-T); (c) p(C6g$_3$T2-T) and (d) p(C8g$_3$T2-T) polymer thin films in their dry, as-cast condition.

5. Organic field effect transistor (OFET) measurements

5.1. p(Cxg_3T2-TT)

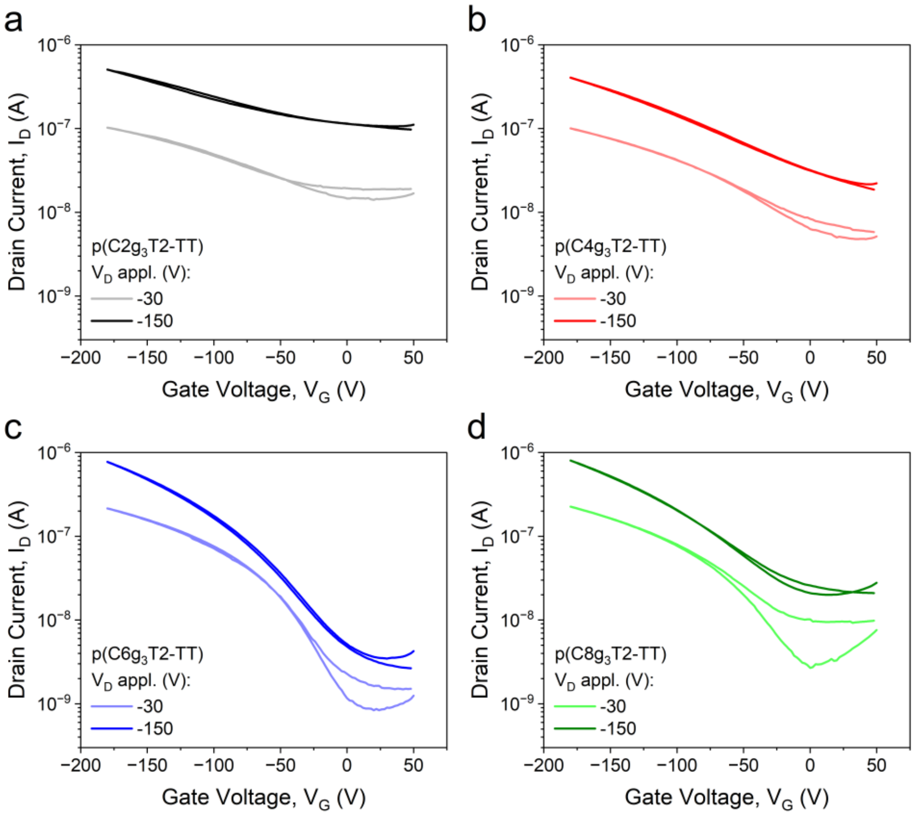

Figure S10. Transfer characteristics of the OFETs made of (a) p(C2g_3T2-TT); (b) p(C4g_3T2-TT); (c) p(C6g_3T2-TT) and (d) p(C8g_3T2-TT).

5.2. p(Cxg_3T2-TT) L-MW

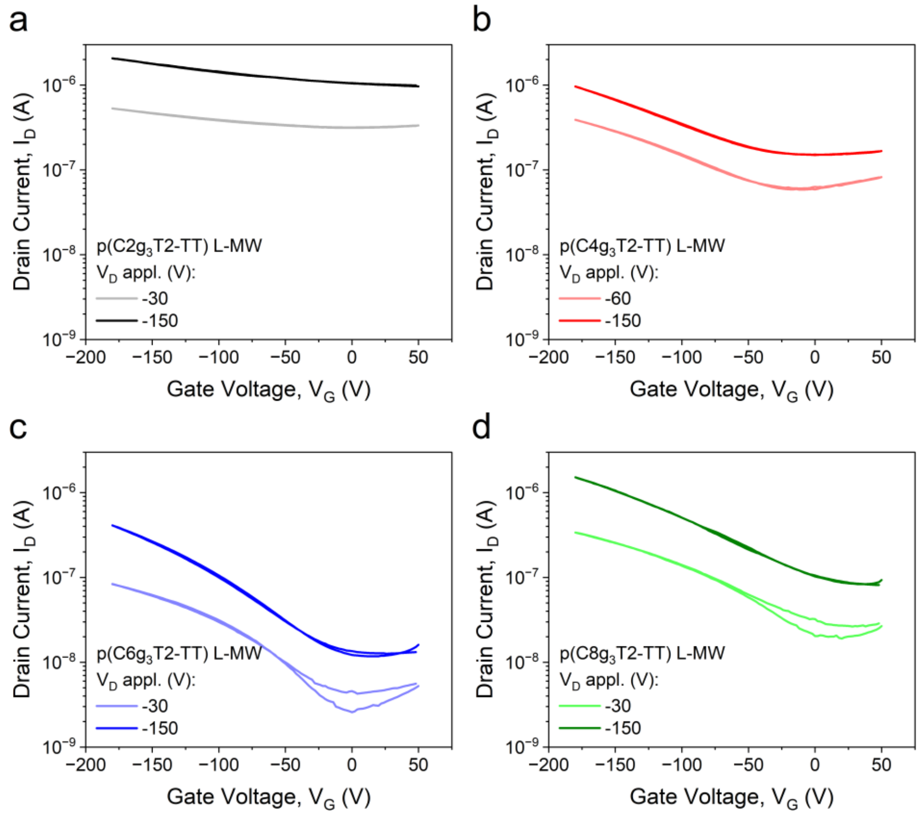

Figure S11. Transfer characteristics of the OFETs made of (a) p($C2g_3T2$-TT) L-MW; (b) p($C4g_3T2$-TT) L-MW; (c) p($C6g_3T2$-TT) L-MW and (d) p($C8g_3T2$-TT) L-MW.

6. Organic electrochemical transistor (OECT) measurements

6.1. p(Cxg$_3$T2-TT)

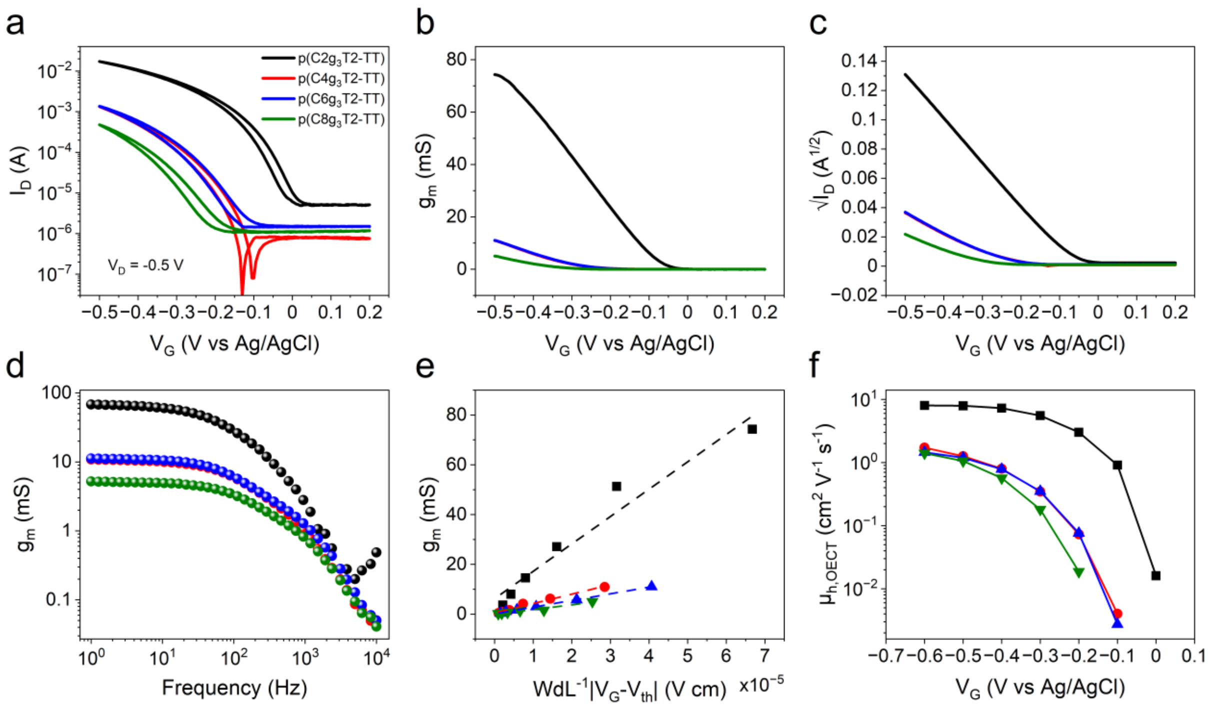

Figure S12. (a) Transfer characteristics of OECTs made of p(C2g$_3$T2-TT) (black), p(C4g$_3$T2-TT) (red), p(C6g$_3$T2-TT) (blue) and p(C8g$_3$T2-TT) (olive). (b) Transconductance and (c) square root of I_D vs V_G plots; fittings in the linear regime of the curves in (c) were used to extract the OECT threshold voltage (V_{th}). (d) Frequency-dependent transconductance plot measured at V_G = -0.5 V and ΔV_G = 10 mV. (e) Transconductance vs channel geometry and operation parameters plot; dashed lines indicate the linear fittings to extract the μC^* value of each polymer. (f) Hole mobility in OECTs vs V_G plot; measurements were performed on the channels that showed the highest mobility for each polymer. Panels (a-e) share the same legend.

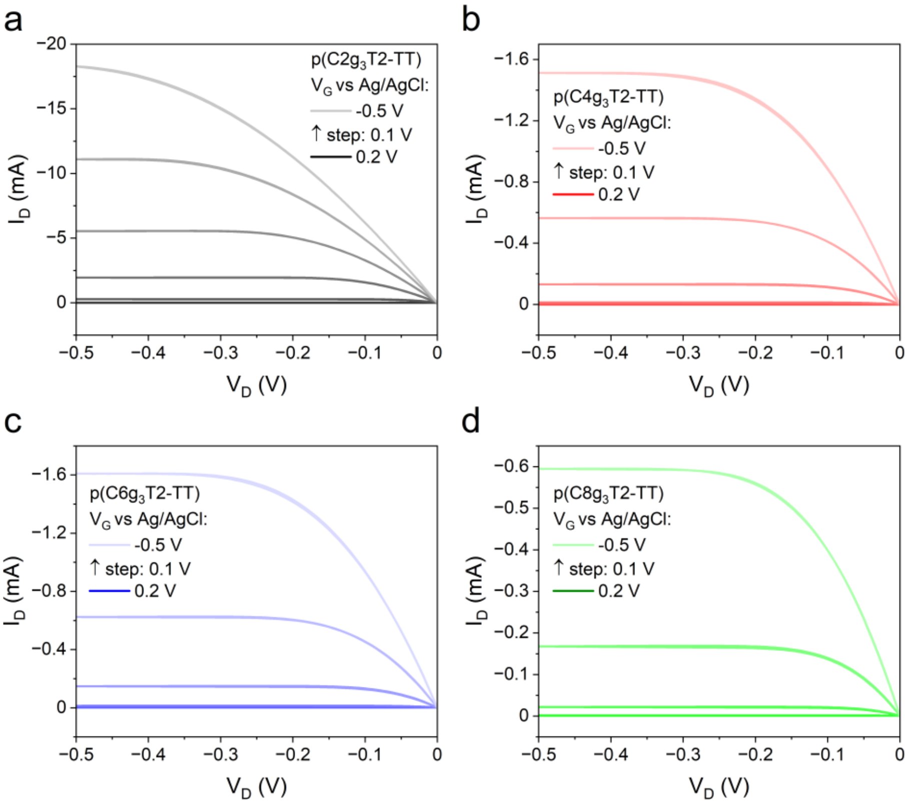

Figure S13. Output characteristics of the OECTs made of (a) p(C2g$_3$T2-TT); (b) p(C4g$_3$T2-TT); (c) p(C6g$_3$T2-TT) and (d) p(C8g$_3$T2-TT).

6.2. p(Cxg$_3$T2-TT) L-MW

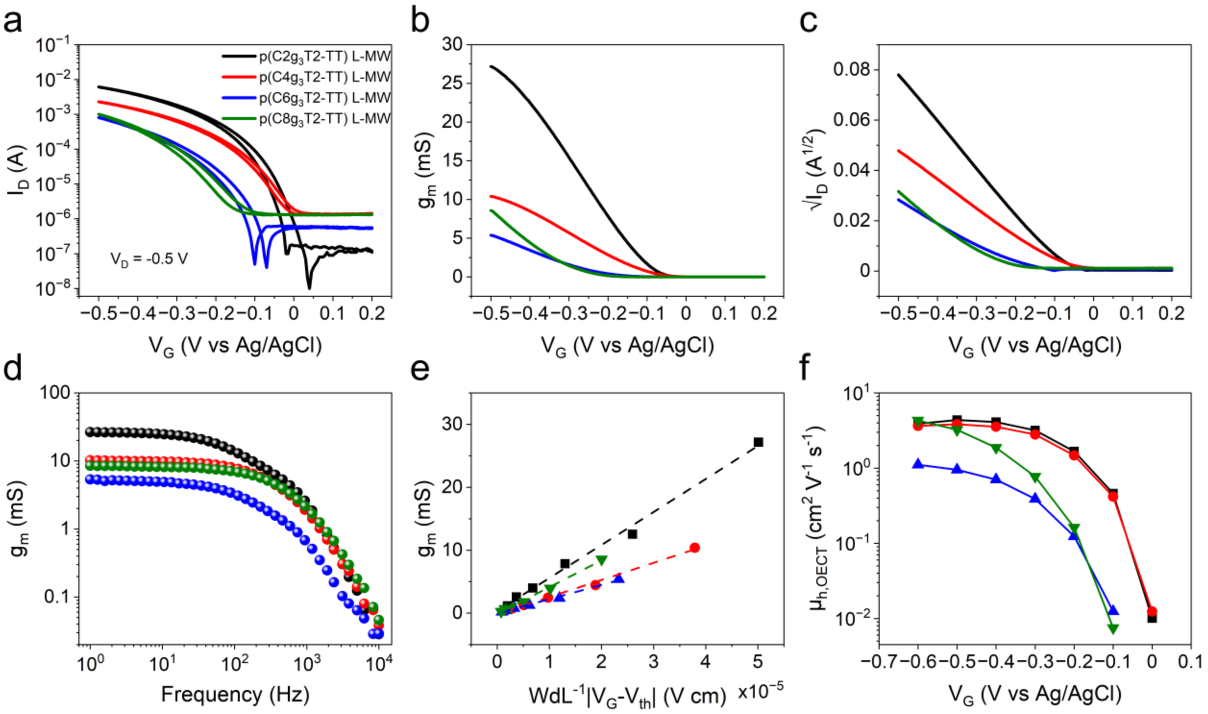

Figure S14. (a) Transfer characteristics of OECTs made of p(C2g$_3$T2-TT) L-MW (black), p(C4g$_3$T2-TT) L-MW (red), p(C6g$_3$T2-TT) L-MW (blue) and p(C8g$_3$T2-TT) L-MW (olive). (b) Transconductance and (c) square root of I_D vs V_G plots; fittings in the linear regime of the curves in (c) were used to extract the OECT threshold voltage (V_{th}). (d) Frequency-dependent transconductance plot measured at V_G = -0.5 V and ΔV_G = 10 mV. (e) Transconductance vs channel geometry and operation parameters plot; dashed lines indicate the linear fittings to extract the μC^* value of each polymer. (f) Hole mobility in OECTs vs V_G plot; measurements were performed on the channels that showed the highest mobility for each polymer. Panels (a-e) share the same legend.

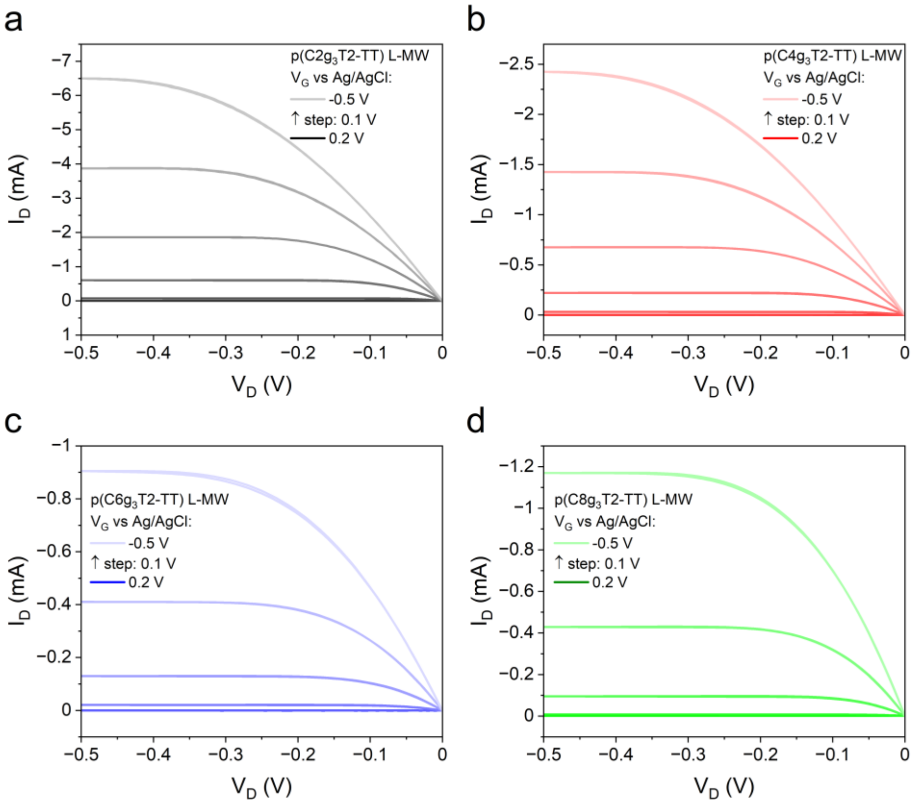

Figure S15. Output characteristics of the OECTs made of (a) p(C2g$_3$T2-TT) L-MW; (b) p(C4g$_3$T2-TT) L-MW; (c) p(C6g$_3$T2-TT) L-MW and (d) p(C8g$_3$T2-TT) L-MW.

7. Electrochemical quartz crystal microbalance (EQCM) measurements

Electronic current from a current collector to a polymer thin film under electrochemical biases can be correlated to a flow of ionic current, i.e. the rate of mass change ($\partial\Delta m/\partial t$), from an electrolyte to the polymer thin film. Analysing the rate of mass change against applied electrochemical potential can be named 'gravimetric cyclic voltammetry' (gCV).[7] The $\partial\Delta m/\partial t$ data was obtained by differentiating the mass change of the thin film during an electrochemical process (Δm) against time and smoothed in the Origin software using the Savitzky–Golay method with a polynomial order of 5 and points of window of 200. All the CV and gCV data shown in this section were chosen from the 2nd CV cycle out of 5 cycles.

7.1. p(Cxg$_3$T2-TT)

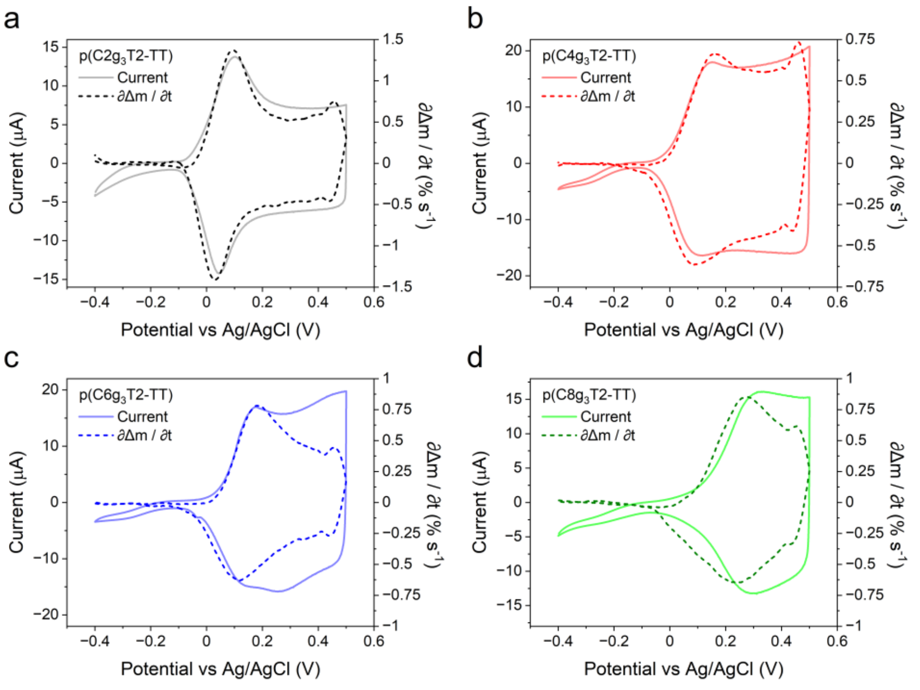

Figure S16. CV and gCV comparison of polymer thin films in Ar-saturated 0.1 M NaCl (aq.) with a scan rate of 10 mV/s for (a) p(C2g$_3$T2-TT); (b) p(C4g$_3$T2-TT); (c) p(C6g$_3$T2-TT) and (d) p(C8g$_3$T2-TT). The good match between the CV and the gCV curves indicates that the current is mostly correlated to the anion insertion/expulsion.

7.2. p(Cxg_3T2-TT) L-MW

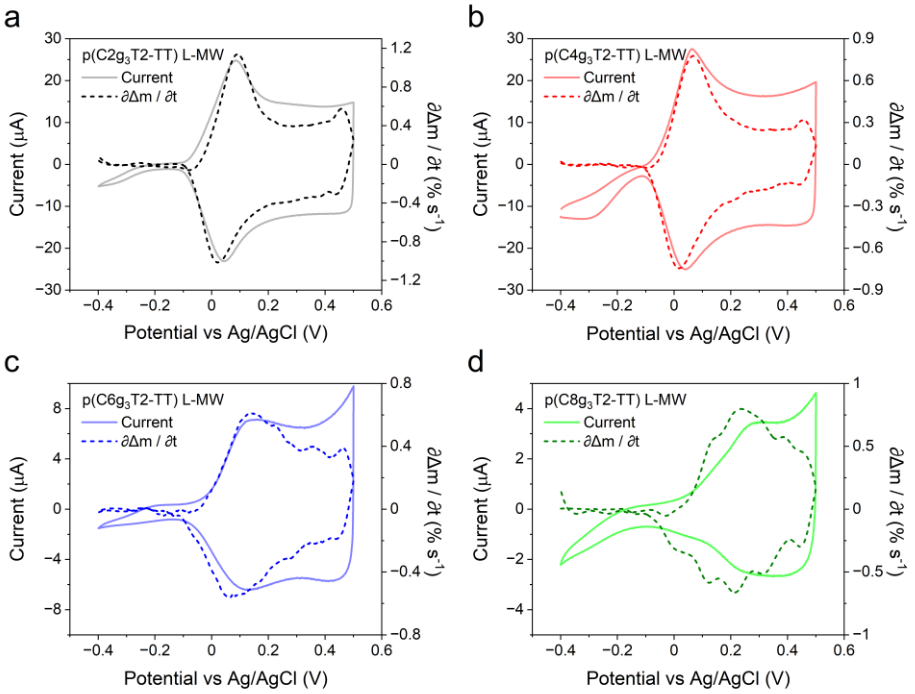

Figure S17. CV and gCV comparison of polymer thin films in Ar-saturated 0.1 M NaCl (aq.) with a scan rate of 10 mV/s for (a) p(C2g_3T2-TT) L-MW; (b) p(C4g_3T2-TT) L-MW; (c) p(C6g_3T2-TT) L-MW and (d) p(C8g_3T2-TT) L-MW. The good match between the CV and the gCV curves indicates that the current is mostly correlated to the anion insertion/expulsion.

8. Quantum chemical simulations

8.1. Methods

Molecules in their neutral, singly and doubly oxidised states were optimised using density functional theory (DFT) with B3LYP/6-311+g(d,p) level of theory in a water or chloroform environment (Solvation Model Based on Density, SMD). Excited state time-dependent density functional theory (TD-DFT) with the same functional, basis set and environment was performed on the molecules in their optimised geometries to calculate the absorption spectra. All the quantum chemical calculations were performed in Gaussian 16, with GaussView 6 to build the molecules and visualise the calculation results.

To reproduce the absorption spectra change of the polymer thin films upon charging the polymers and minimise the calculation complexity, dimers ($(gT2\text{-}TT)_2$ and $(gT2\text{-}T)_2$) with methoxy side chains of the p(Cxg_3T2-TT) and the p(Cxg_3T2-T) polymer series (Figure S17) and the water (SMD) environment were used in the calculations. The choice was based on the good match between their calculated spectra and experimental results and the fact that side chains have little influence on the electronic structure of the conjugation system.[8,9]

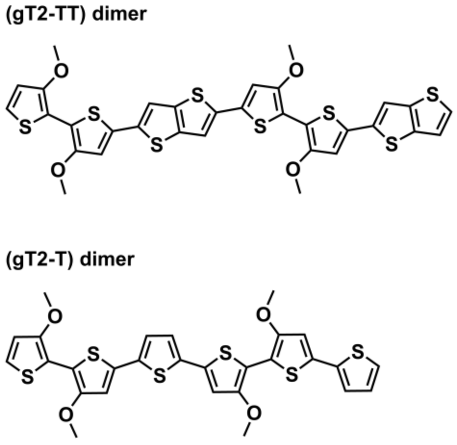

Figure S18. Chemical structures of the (gT2-TT) and (gT2-T) dimers.

As predicted by the MD simulation, in polar environments, p(Cxg_3T2-TT) polymers with alkyl spacer lengths longer than 2 (x =4, 6, 8) on the side chains exhibit a large dihedral angle within the oxy-bithiophene groups ($\phi_{gT\text{-}gT} \approx 75°$), which leads to their more twisted backbones and blue-shifted absorption spectra compared with p($C2g_3T2$-TT). To reproduce the blue-shifted absorption spectra of p(Cxg_3T2-TT) (x =4, 6, 8) and minimise the calculation complexity, we performed (TD-)DFT calculations on neutral (gT2-TT) trimers ($(gT\text{-}TT)_3$) using B3LYP/6-311+g(d,p) level of theory in a water (SMD) environment. Prior to the geometry optimisation, the dihedral angles of the middle repeat

unit of the (gT2-TT) trimers were manually set as $\phi_{gT\text{-}gT}$ = 17° or 75° and $\phi_{gT\text{-}TT}$ = 25°, adopted from the MD simulation results on p(Cxg$_3$T2-TT) single chains in 0.2 M NaCl (aq.). Their absorption spectra were then calculated using the optimised geometry.

8.2. Results

8.2.1. (gT2-TT)$_2$ and (gT2-T)$_2$ at different oxidised states

Table S3. Summary of the calculated energies (≤ 3.10 eV, i.e. ≥ 400 nm) and the oscillator strengths of the neutral, singly (1+) and doubly (2+) oxidised (gT2-TT)$_2$ in the water environment (SMD).

Neutral (gT2-TT)$_2$		(gT2-TT)$_2^{1+}$		(gT2-TT)$_2^{2+}$	
Energy (eV) [Wavelength (nm)]	Oscillator strength	Energy (eV) [Wavelength (nm)]	Oscillator strength	Energy (eV) [Wavelength (nm)]	Oscillator strength
1.98 [625.6]	2.5333	0.78 [1599.5]	0.7458	1.25 [989.7]	3.3521
2.49 [497.9]	0.0009	1.46 [850.3]	1.1974	1.77 [700.6]	0.0738
2.65 [468.3]	0.0008	1.55 [799.1]	0.6863	2.14 [580.6]	0.0025
3.08 [402.0]	0.0355	1.72 [722.7]	0.2609	2.22 [557.3]	0.0008
		1.98 [626.9]	0.0093	2.27 [546.6]	0.0338
		2.14 [580.5]	0.0007	2.30 [538.1]	0.0322
		2.18 [568.4]	0.0131	2.46 [503.7]	0.0160
		2.25 [551.7]	0.0014	2.56 [485.2]	0.0219
		2.29 [541.3]	0.0139	2.57 [482.9]	0.0048
		2.31 [536.3]	0.0110	2.74 [451.9]	0.0196
		2.37 [523.3]	0.0127	2.84 [436.5]	0.0036
		2.38 [521.4]	0.0080		
		2.46 [503.1]	0.0003		
		2.63 [471.0]	0.0273		
		2.72 [455.9]	0.0230		
		2.75 [450.4]	0.0152		
		2.92 [425.1]	0.0678		
		2.96 [418.3]	0.0544		

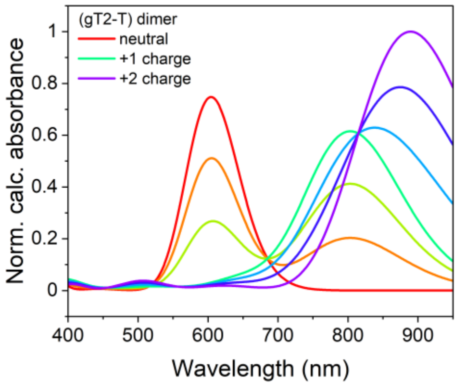

Figure S19. Normalised calculated absorbance spectra of the (gT2-T) dimers in their neutral (red), singly (green) and doubly (purple) oxidised states. Linear combinations of the absorbance spectra between the neutral and singly oxidised state and between the singly and doubly oxidised state are shown to reproduce the experimental absorbance spectra change.

Table S4. Summary of the calculated energies (≤ 3.10 eV, i.e. ≥ 400 nm) and the oscillator strengths of the neutral, singly (1+) and doubly (2+) oxidised $(gT2\text{-}T)_2$ in the water environment (SMD).

Neutral $(gT2\text{-}T)_2$		$(gT2\text{-}T)_2^{1+}$		$(gT2\text{-}T)_2^{2+}$	
Energy (eV) [Wavelength (nm)]	Oscillator strength	Energy (eV) [Wavelength (nm)]	Oscillator strength	Energy (eV) [Wavelength (nm)]	Oscillator strength
2.05 [604.3]	2.0287	0.84 [1468.4]	0.5267	1.39 [889.4]	2.7140
2.60 [476.4]	0.0021	1.54 [806.4]	1.6063	1.99 [624.4]	0.0509
2.81 [441.9]	0.0176	1.68 [740.3]	0.1059	2.37 [522.3]	0.0142
		1.89 [654.7]	0.1362	2.40 [516.4]	0.0585
		2.14 [579.6]	0.0213	2.49 [498.9]	0.0041
		2.29 [540.4]	0.0004	2.53 [490.2]	0.0343
		2.34 [529.1]	0.0157	2.64 [469.7]	0.0129
		2.37 [522.5]	0.0046	2.92 [424.1]	0.0083
		2.38 [520.7]	0.0110	2.95 [420.9]	0.0031
		2.45 [505.2]	0.0051	3.06 [405.2]	0.0521
		2.61 [475.4]	0.0228		
		2.77 [447.7]	0.0051		
		2.84 [436.6]	0.0016		
		2.92 [425.3]	0.0032		
		2.97 [417.4]	0.0349		
		2.99 [415.2]	0.0078		

8.2.2. $(gT\text{-}TT)_3$ with flat and twisted backbone

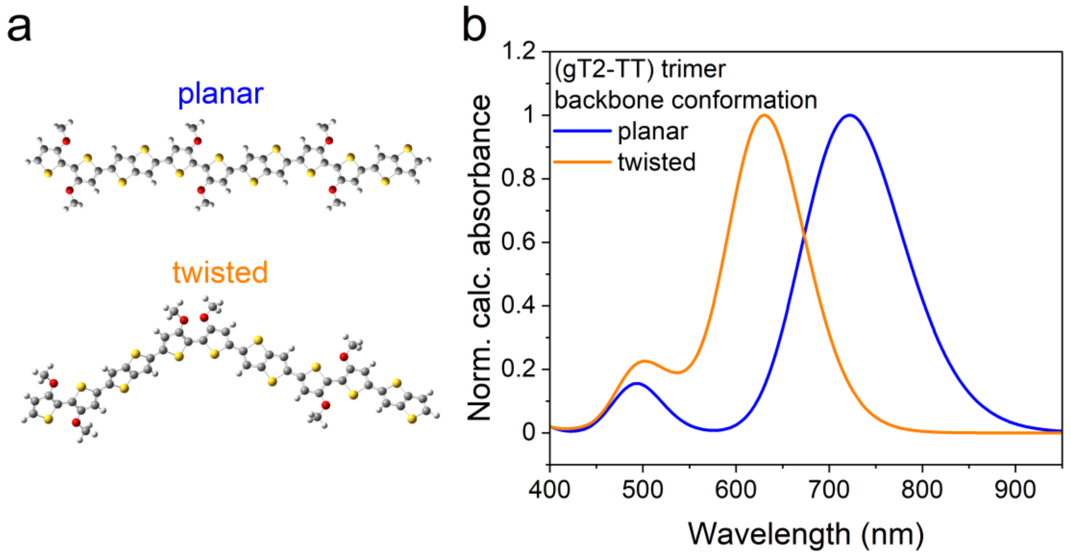

Figure S20. (a) Schematic representation of (gT2-TT) trimers with a planar and a twisted backbone conformation. Colour scheme: carbon (grey), oxygen (red), sulfur (yellow), hydrogen (light grey). (b) Normalised calculated absorbance spectra of the neutral (gT2-TT) trimers with the two backbone conformations. The calculations were performed using TD-DFT with B3LYP/6-311+g(d,p) level of theory in a water (SMD) environment.

Table S5. Summary of the calculated energies (≤ 3.10 eV, i.e. ≥ 400 nm) and the oscillator strengths of the neutral (gT2-TT) trimers with flat and twisted backbones in the water environment (SMD).

$(gT2\text{-}TT)_3$ flat		$(gT2\text{-}TT)_3$ twisted	
Energy (eV) [Wavelength (nm)]	Oscillator strength	Energy (eV) [Wavelength (nm)]	Oscillator strength
1.72 [721.85]	3.8873	1.97 [630.88]	3.1982
2.13 [583.03]	0.0038	2.26 [549.36]	0.2448
2.18 [570.09]	0.0001	2.32 [534.03]	0.0983
2.46 [504.60]	0.0094	2.50 [496.16]	0.6124
2.49 [497.41]	0.4903	2.66 [466.89]	0.0467
2.60 [476.12]	0.1441	2.74 [452.29]	0.0139
2.82 [439.04]	0.0022	2.90 [427.74]	0.0223
2.93 [423.68]	0.0045	2.97 [417.29]	0.0042
		3.08 [402.55]	0.0101

After being optimised by DFT calculations, the (gT2-TT) trimers either feature a planar backbone conformation with both $\phi_{gT\text{-}gT}$ and $\phi_{gT\text{-}TT} = 0°$ or significant twisting at the middle oxybithiophene group with $\phi_{gT\text{-}gT} = 131°$ and $\phi_{gT\text{-}TT} = 19°$. (Figure S19a). For the (gT2-TT) trimer with a planar backbone, its calculated absorption spectrum shows a pronounced absorption peak at 722 nm due to the HOMO to

LUMO transition and a weak absorption peak at 493 nm due to the HOMO-1 to LUMO+1 transition (Figure S19b). In comparison, the (gT2-TT) trimer with a twisted backbone shows its main absorption peak at 630 nm, which is significantly blue-shifted by 92 nm compared to that of the flat (gT2-TT) trimer, whilst the weak satellite absorption peak of the twisted (gT2-TT) trimer undergoes a slight red shift to 503 nm. This red shift is likely a result of the B3LYP functional over-delocalising the charge density.

9. Molecular dynamics simulation

Gromacs 2018.2 (reference 10) was used to perform molecular dynamics simulations on oligomers of the p(Cxg$_3$T2-TT), p(Cxg$_3$T2-T) and p(Cxg$_3$T2) (x = 2, 4, 6, 8) polymers, using force fields based on those published in references 11 and 12. For each sidechain and backbone combination, Gromacs was used to simulate initial conformations for solvated oligomers of 14, 16 and 20 repeat units for each backbone type respectively, different numbers of repeat units were selected to maintain oligomers of similar lengths between all simulation variants. Four replicas of each oligomer were simulated to sample a wider range of potential conformations. For each simulation, three equilibration steps were carried out starting with energy minimisation. Energy minimisation was completed in 1 fs timesteps using the steepest decent algorithm. The minimisation was considered converged when the change in energy between subsequent steps was lower than the available machine precision. After energy minimisation, equilibration was carried out in both the NVT and NPT ensembles. Starting with NVT, oligomers were simulated for 2500000 steps at 298 K with 2 fs timesteps followed by a final equilibration in the NPT ensemble at 1 bar for 50 ns. Finally, a production run was performed for 200 ns in the NPT ensemble with Berendsen pressure coupling, for temperature coupling the velocity rescale thermostat was used.[13] The Verlet cut-off scheme was applied for neighbour searching and particle-mesh-Ewald summation was used for electrostatics and Van der Waals force calculations with a cut-off of 1 nm.[14,15]

The procedure described above simulates isolated oligomers in solvent, we also report the simulation of amorphous films starting from the previously simulated single chains. In order to build a film that reflects a realistic microstructure we simulated 100 oligomers in their solvated state before completing an iterative evaporation procedure. This simulates experimental film formation from solution. The first step uses PackMol to place 100 chains (25 $\times$ 4 replicas) from the previous simulations in a box in random orientations to provide suitable conformational diversity. The box is then solvated with 400000 water molecules. This system then goes through an energy minimisation process followed by an NVT equilibration step for 1 ns and a 40 ns NPT run, simulation conditions are the same as described above. The evaporation process is then simplified into 11 water removal steps, every 40 ns 40000 water molecules are removed from the simulation cell and the system is re-equilibrated. This process is repeated until all water has been removed. What is left is a dry amorphous film, the film then undergoes a final NPT simulation for 40 ns.

After simulation the dihedral angle distribution is extracted for all backbone dihedrals, these dihedrals are separated into “inner”, between the T-T in the Cxg$_3$T2 unit, and the “outer”, between a T in the Cxg$_3$T2 unit and the adjacent backbone moiety. For the single solvated chains the time-averaged dihedral angle was calculated for each dihedral, this was then averaged across the oligomer for each dihedral type. Further averaging across the oligomer replicas was carried out to give the reported values.

The analysis remains the same between films and single solvated oligomer since the only difference is the number of samples, instead of extracting the average dihedral angles for each oligomer this is done on a film-wide basis.

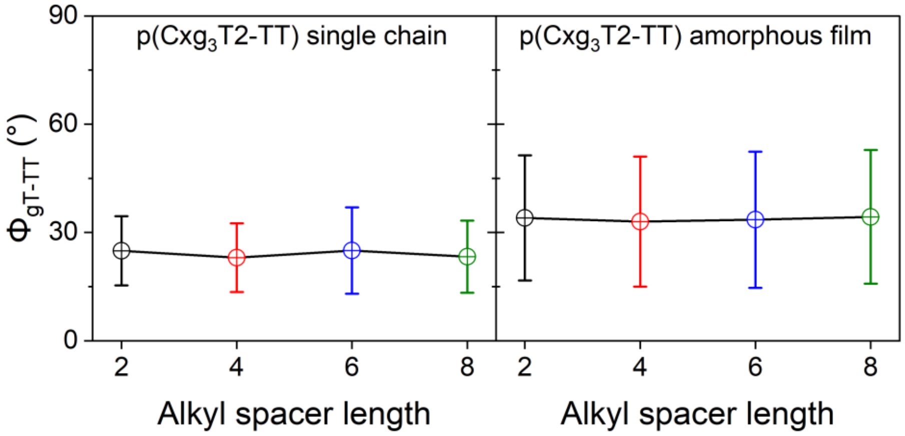

Figure S21. Average $\phi_{gT\text{-}TT}$ values, extracted from atomistic MD simulations, of p(Cxg$_3$T2-TT) (x = 2, 4, 6, 8) (14 repeat units) single chains and amorphous films composed of 100 respective single chains in 0.2 M NaCl (aq.) solutions.

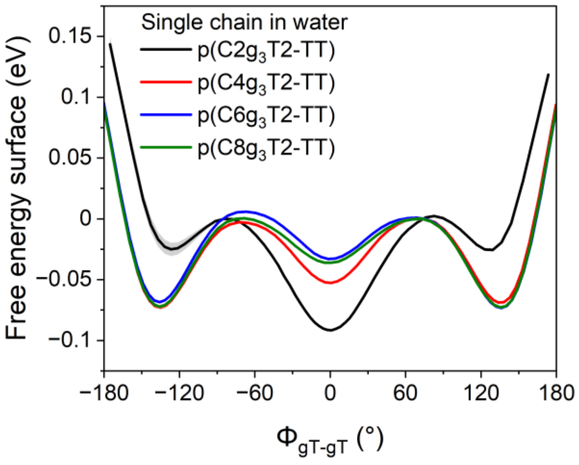

Figure S22. Free energy surface of p(Cxg$_3$T2-TT) (x = 2, 4, 6, 8) single chains in water as a function of $\phi_{gT\text{-}gT}$.

10. Materials synthesis and characterisations

All chemicals were purchased from commercial suppliers (Sigma Aldrich or VWR Scientific) unless otherwise specified. Column chromatography was conducted with silica gel for flash chromatography supplied from VWR Scientific. ^{1}H- and ^{13}C-NMR spectra were obtained on a Bruker AV-400 spectrometer at 298 K and are reported in ppm relative to a TMS chemical shift reference. Deuterated solvents were purchased from Sigma Aldrich. UV-Vis absorption spectra were recorded on a UV-1601 UV-Vis spectrometer at 298 K. MALDI-ToF mass spectroscopy was performed on a Micromass MALDImxTOF using trans-2-[3-(4-tert-butylphenyl)-2-methyl-2-propenylidene]malononitrile (DCTB) as the matrix.

10.1. Synthesis of 13-(thiophen-3-yloxy)-2,5,8,11-tetraoxatridecane

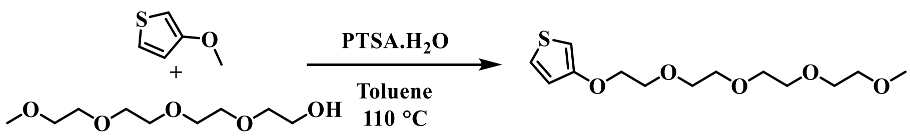

3-Methoxythiophene (6.1 mL, 61.4 mmol, 1.0 eq.) was dissolved in 70 mL toluene, inside a 2-neck 250 mL RBF. 2,5,8,11-tetraoxatridecan-13-ol (18.4 mL, 92.1 mmol, 1.5 eq.) was added dropwise, followed by the addition of *p*-toluenesulfonic acid monohydrate (1.16 g, 6.14 mmol, 0.1 eq.) in a single portion. The reaction was refluxed overnight at 110 °C before being cooled to room temperature poured into water, washed with brine and extracted into DCM. The organic layer was separated, dried over $MgSO_4$ and the solvent removed under reduced pressure. The crude was purified via column chromatography on silica gel using DCM as the eluent to afford a yellow oil (9.99 g, 56%).

^{1}H NMR (400 MHz, Chloroform-*d*) δ 7.15 (ddd, *J* = 5.2, 3.1, 1.2 Hz, 1H), 6.77 (dt, *J* = 5.2, 1.4 Hz, 1H), 6.25 (dt, *J* = 3.1, 1.4 Hz, 1H), 4.11 (ddd, *J* = 4.9, 4.1, 1.2 Hz, 2H), 3.83 (td, *J* = 4.7, 1.2 Hz, 2H), 3.75 – 3.48 (m, 12H), 3.37 (s, 3H).

^{13}C NMR (101 MHz, Chloroform-*d*) δ 157.71, 124.74, 119.71, 97.63, 72.05, 70.90, 70.74, 70.63, 69.80, 69.70, 59.14.

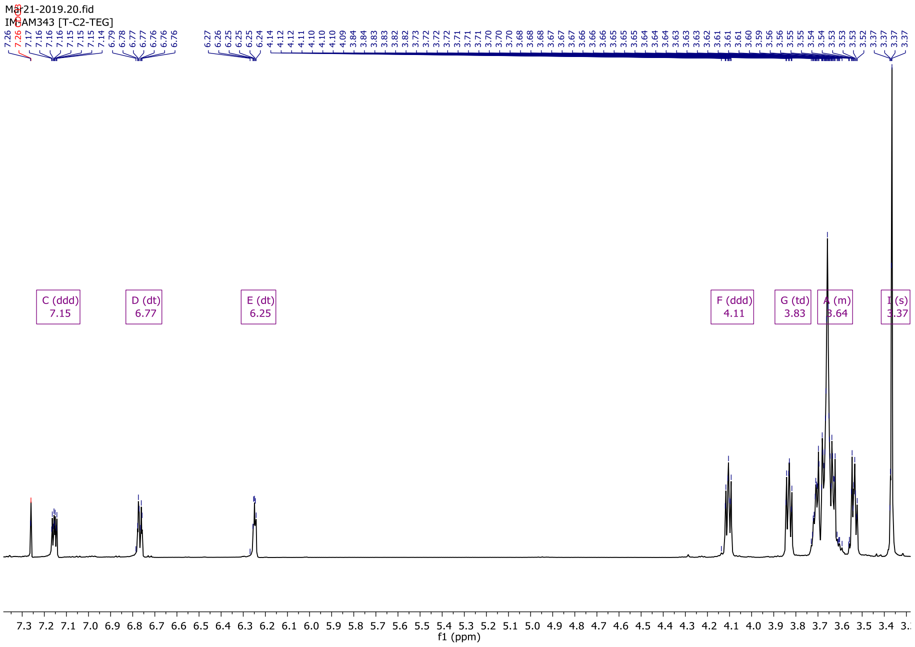

Figure S23. ^{1}H NMR in $CDCl_3$.

10.2. Synthesis of 3,3'-bis((2,5,8,11-tetraoxatridecan-13-yl)oxy)-2,2'-bithiophene

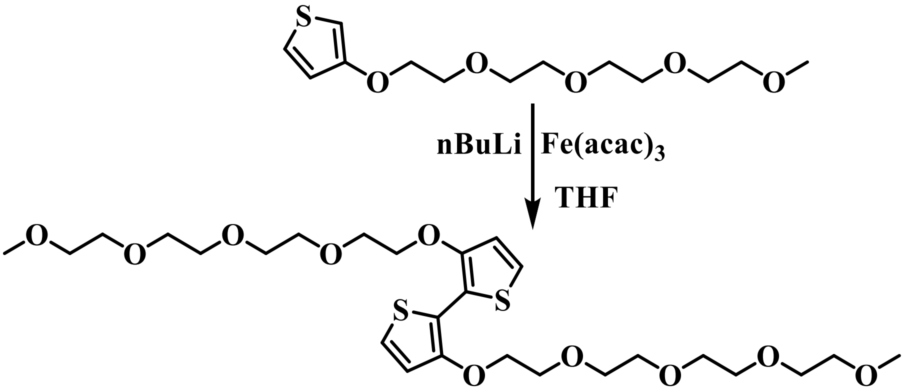

13-(thiophen-3-yloxy)-2,5,8,11-tetraoxatridecane (9.99 g, 34.4 mmol, 1.0 eq.) was dissolved in dry THF (100 mL) cooled to -10 °C, under a Nitrogen atmosphere. *n*-BuLi (1.6 M, 16.5 mL, 41.3 mmol, 1.2 eq.) was added slowly dropwise and the reaction was stirred for 2 hours at 0 °C. In a separate flask $Fe(acac)_3$ (12.15 g, 34.4 mmol, 1.0 eq.) was dissolved in 100 mL anhydrous THF, under a Nitrogen atmosphere, cooled to 0 °C. The lithiate solution was transferred via cannula to the $Fe(acac)_3$ solution over a period of 15 minutes. The reaction was heated at reflux for 2 hours before cooling to room temperature and passing through a short silica plug, eluting with ethyl acetate. The crude material was purified by column chromatography on silica gel using ethyl acetate as the eluent system. Solvent was removed under reduced pressure to yield a yellow oil (4.41 g, 44%).

^{1}H NMR (400 MHz, Chloroform-*d*) δ 7.07 (d, *J* = 5.6 Hz, 2H), 6.85 (d, *J* = 5.6 Hz, 2H), 4.24 (t, *J* = 4.9 Hz, 4H), 3.90 (d, *J* = 4.9 Hz, 4H), 3.75 (dd, *J* = 6.1, 3.7 Hz, 4H), 3.72 – 3.58 (m, 16H), 3.59 – 3.49 (m, 4H), 3.36 (s, 6H).

^{13}C NMR (101 MHz, Chloroform-*d*) δ 151.84, 122.03, 116.68, 114.88, 72.06, 71.51, 71.05, 70.81, 70.75, 70.65, 70.15, 59.15.

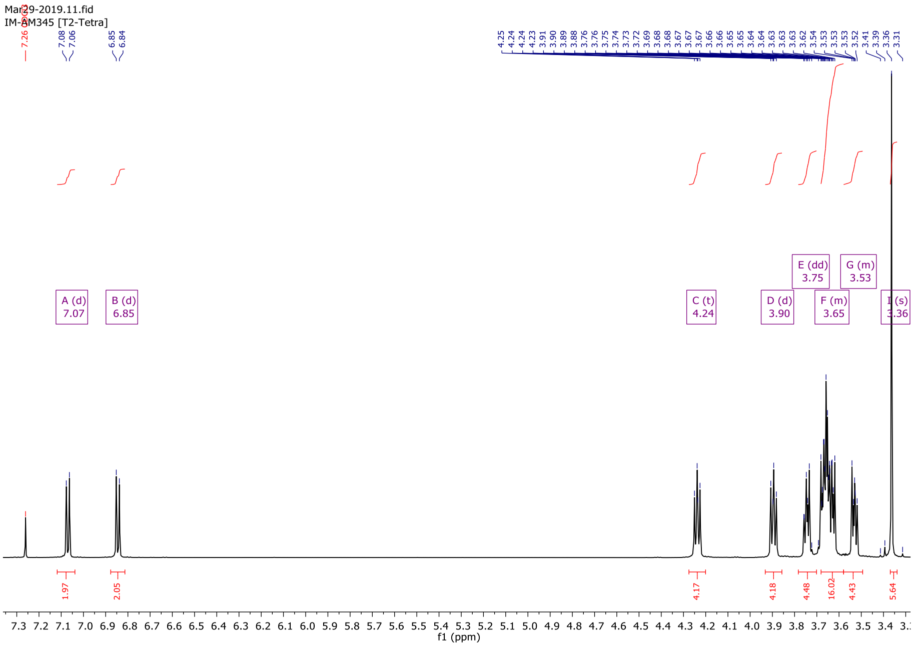

Figure S24. ^{1}H NMR in $CDCl_3$.

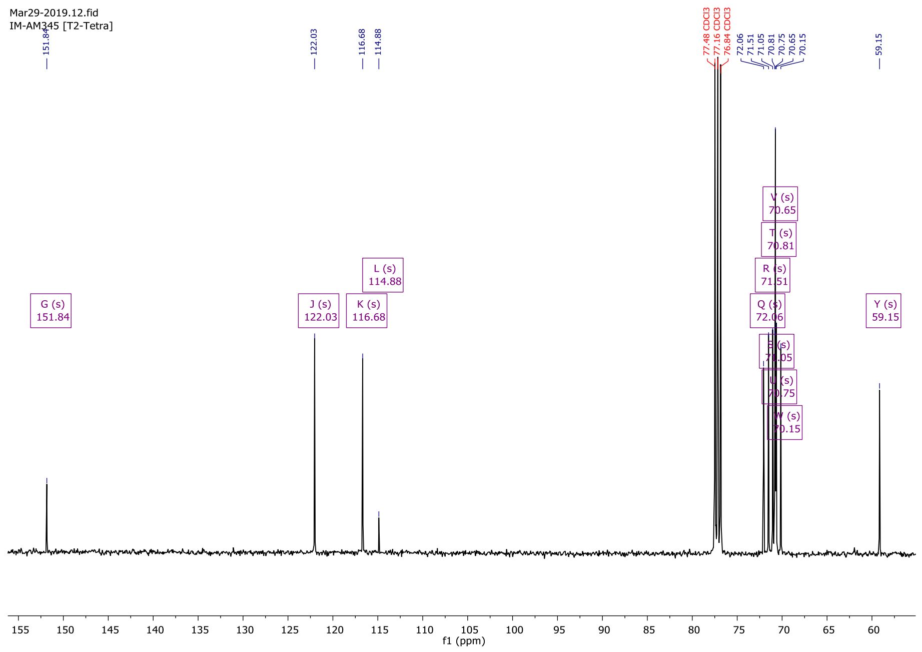

Figure S25. ^{13}C NMR in $CDCl_3$.

10.3. Synthesis of 13,13'-((5,5'-dibromo-[2,2'-bithiophene]-3,3'-diyl)bis(oxy))bis(2,5,8,11-tetraoxatridecane)

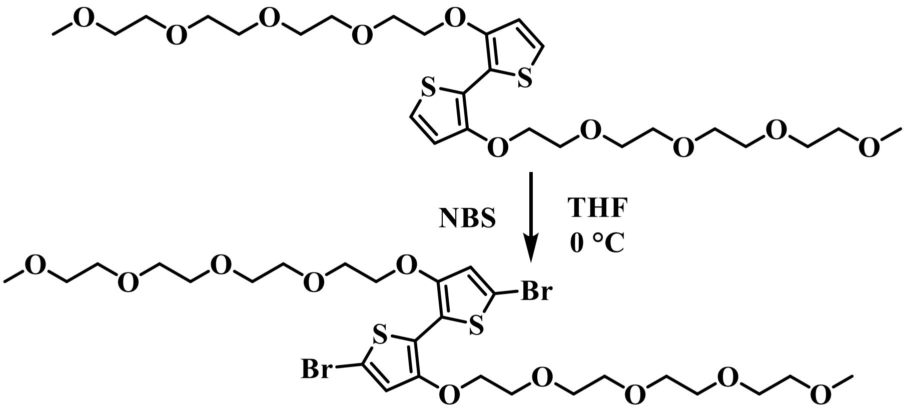

3,3'-bis((2,5,8,11-tetraoxatridecan-13-yl)oxy)-2,2'-bithiophene (2 g, was added to an oven dried 2-neck 100 mL RBF, dissolved in dry THF (40 mL) under a Nitrogen atmosphere. Cooled to 0 °C and covered from light prior to the portion wise addition of N-bromosuccinimide (4 × 325 mg, 7.27 mmol, 2.1 eq.) leaving approximately 5 minutes between the addition of each portion. The reaction was stirred for 45 minutes before being poured into water, washed with saturated sodium carbonate and quenched with sodium metabisulphite. The organic layer was extracted into DCM and dried over Na_2SO_4; solvent removed under reduced pressure. The crude was purified by column chromatography, on silica gel, using ethyl acetate as the eluent to afford a yellow oil which solidified at low temperature (2.46 g, 97%).

^{1}H NMR (400 MHz, Chloroform-*d*) δ 6.85 (s, 2H), 3.90 – 3.82 (m, 4H), 3.78 – 3.60 (m, 16H), 3.57 – 3.50 (m, 4H), 3.37 (s, 6H).

^{13}C NMR (101 MHz, Chloroform-*d*) δ 150.31, 119.77, 115.97, 110.18, 72.06, 71.82, 71.03, 70.80, 70.75, 70.63, 70.00, 59.16, 29.71.

Mass (MALDI-ToF): 736.9 $[M+H]^+$ (calc. 736.0 $C_{26}H_{40}Br_2O_{10}S_2$).

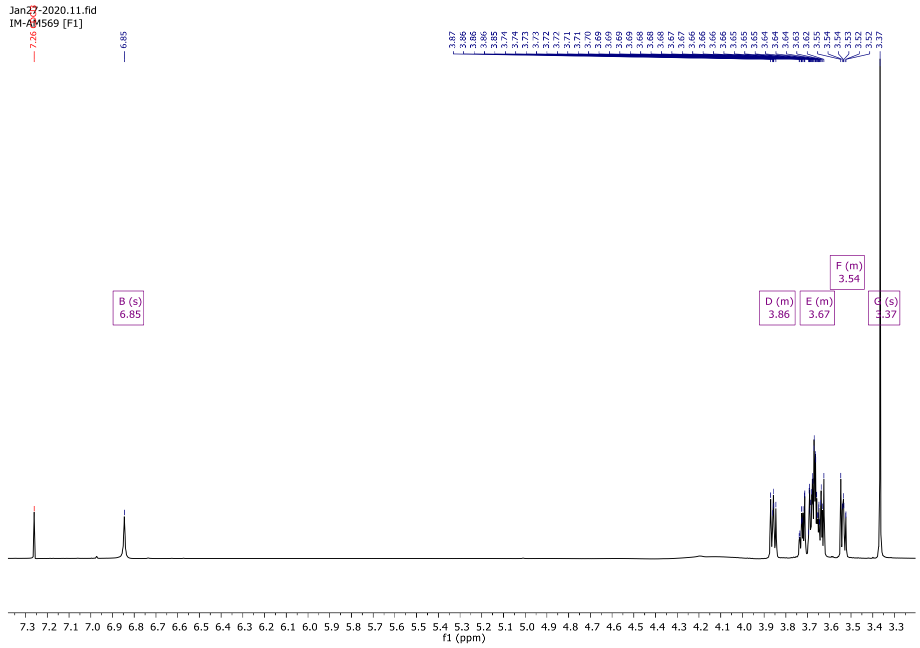

Figure S26. ^{1}H NMR in $CDCl_3$.

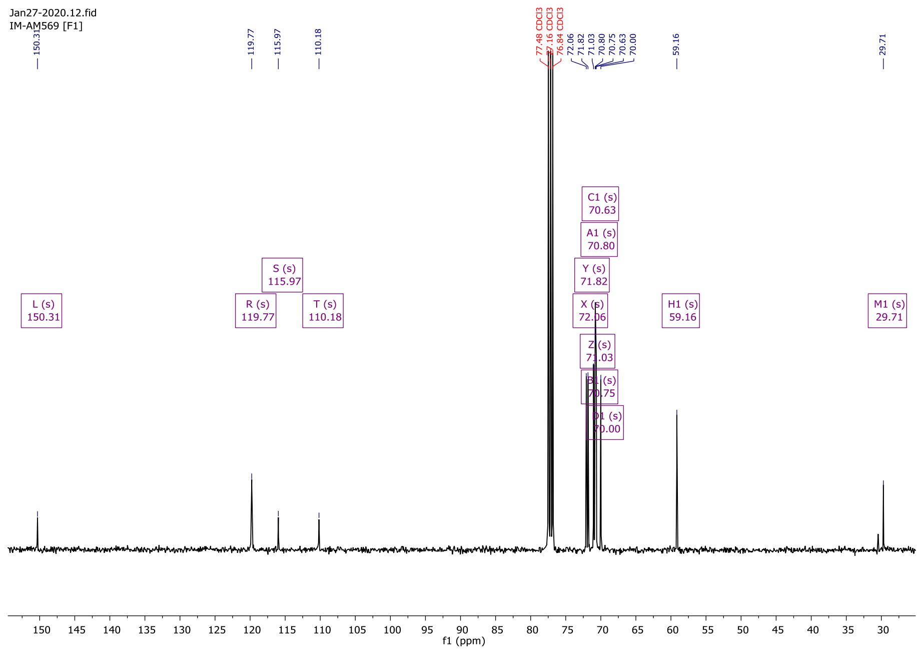

Figure S27. ^{13}C NMR in $CDCl_3$.

10.4. Synthesis of Polymer p(C2g$_3$T2-TT)

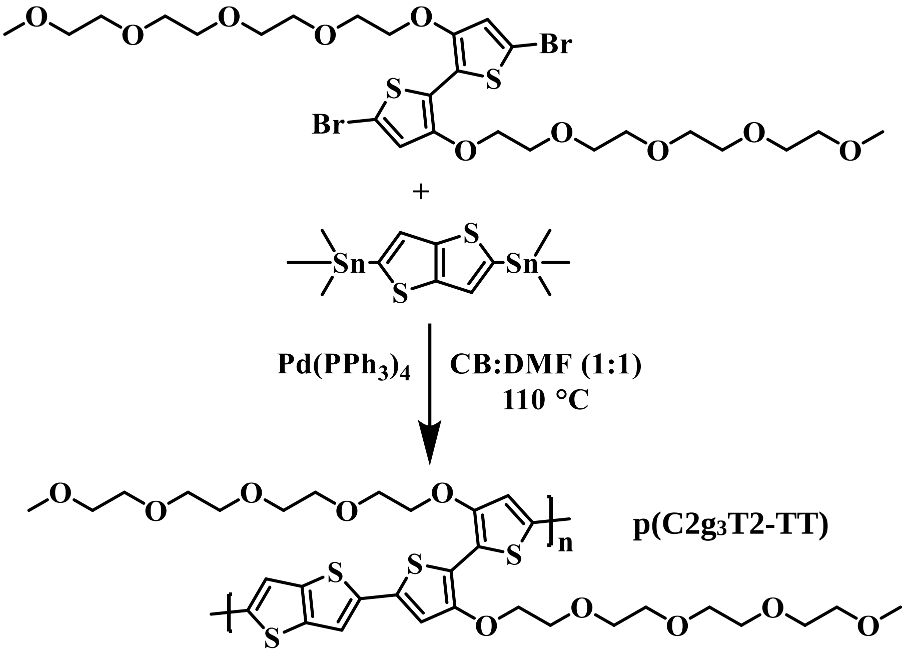

An oven dried 5 mL microwave vial was charged with 13,13'-((5,5'-dibromo-[2,2'-bithiophene]-3,3'-diyl)bis(oxy))bis(2,5,8,11-tetraoxatridecane) (200 mg, 0.31 mmol, 1.0 eq.), 2,5-bis(trimethylstannyl)thieno[3,2-*b*]thiophene (144 mg, 0.31 mmol, 1.0 eq.) and $Pd(PPh_3)_4$ (7.1 mg, 0.006 mmol, 0.02 eq.). Cap was sealed and the vial was purged with Nitrogen for 5 minutes prior to the addition of anhydrous DMF (2.06 mL) and anhydrous chlorobenzene (2.06 mL). The reaction was

stirred overnight at 110 °C then cooled to room temperature, upon which the solution formed a purple gel. Crude polymer was precipitated into 100 mL methanol and subsequently filtered into a thimble. Purification was conducted via Soxhlet extraction, washing with hexane, methanol, acetone, ethyl acetate and chloroform (in that order). The chloroform fraction was collected, solvent removed under reduced pressure and re-precipitated into 100 mL methanol, filtered to yield a blue metallic film (140 mg, 61%). Gel-Permeation Chromatogram (GPC) (DMF, 40 °C): M_n 83.3 kDa, M_w 113.3 kDa, $Đ$ 1.4.

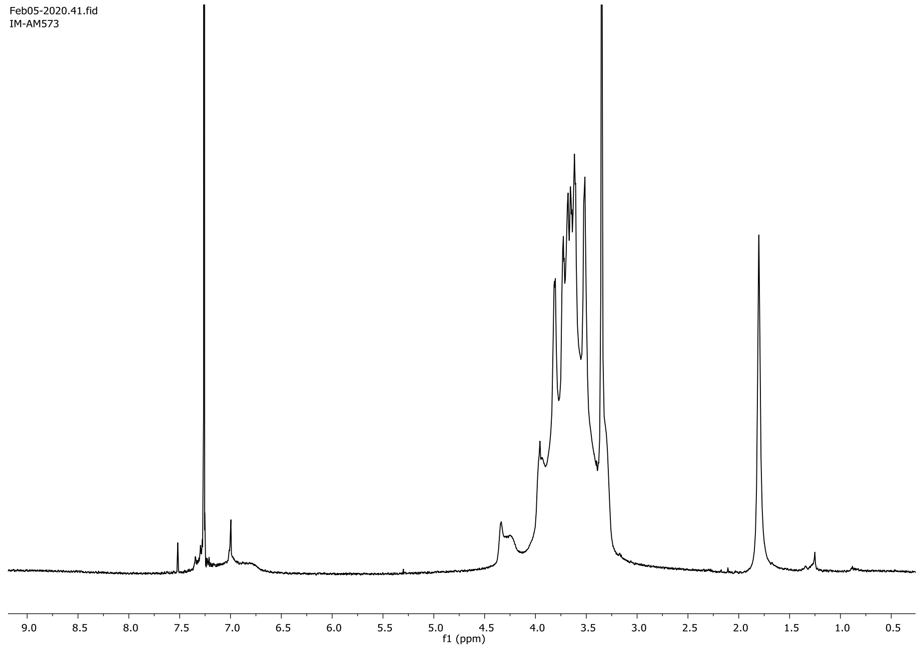

Figure S28. ^{1}H NMR in $CDCl_3$.

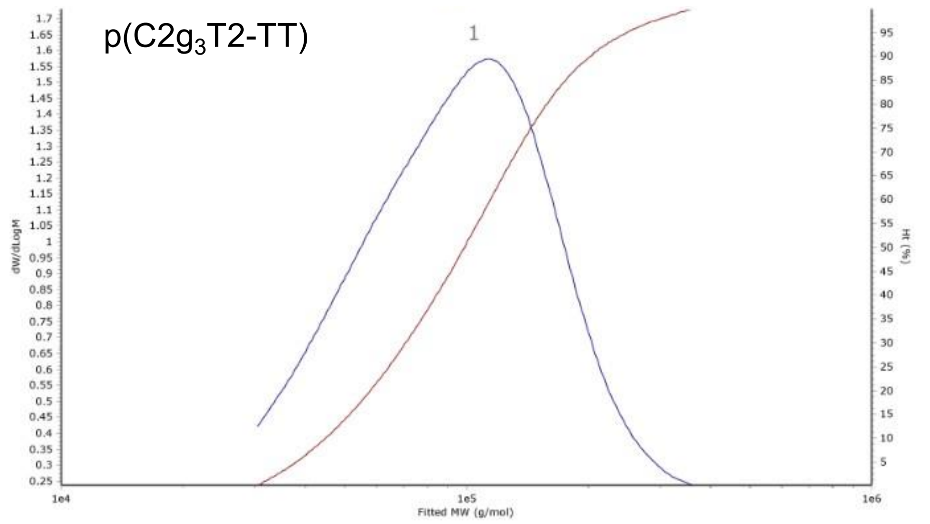

Figure S29. GPC spectra. M_n, M_w, and $Đ$ (M_w/M_n) were determined by GPC using low-D (<1.10) polystyrene standards and DMF as the eluent at 40 °C.

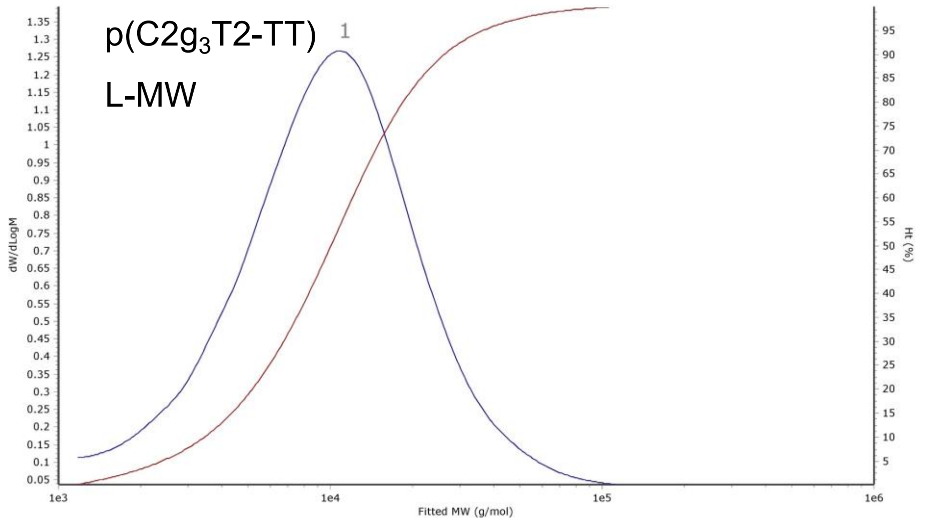

Figure S30. GPC spectra. M_n, M_w, and $Đ$ (M_w/M_n) were determined by GPC using low-D (<1.10) polystyrene standards and DMF as the eluent at 40 °C.

10.5. Synthesis of Polymer p(C2g$_3$T2-T)

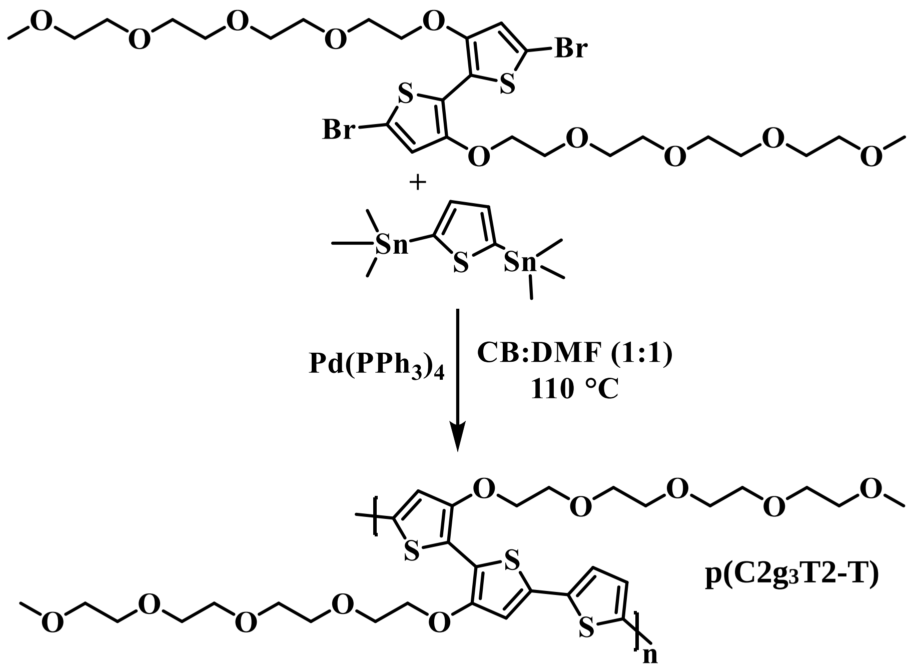

An oven dried 5 mL microwave vial was charged with 13,13'-((5,5'-dibromo-[2,2'-bithiophene]-3,3'-diyl)bis(oxy))bis(2,5,8,11-tetraoxatridecane) (200 mg, 0.31 mmol, 1.0 eq.), 2,5-bis(trimethylstannyl)thiophene (126.4 mg, 0.31 mmol, 1.0 eq.) and $Pd(PPh_3)_4$ (7.1 mg, 0.006 mmol, 0.02 eq.). Cap was sealed and the vial was purged with Nitrogen for 5 minutes prior to the addition of anhydrous DMF (2.06 mL) and anhydrous chlorobenzene (2.06 mL). The reaction was stirred overnight

at 110 °C then cooled to room temperature, upon which the solution formed a purple gel. Crude polymer was precipitated into 100 mL methanol and subsequently filtered into a thimble. Purification was conducted via Soxhlet extraction, washing with hexane, methanol, acetone, ethyl acetate and chloroform (in that order). The chloroform fraction was collected, solvent removed under reduced pressure and re-precipitated into 100 mL methanol, filtered to yield a blue metallic film (167 mg, 79%). GPC (DMF, 40 °C): M_n 51.1 kDa, M_w 109.6 kDa, *Đ* 2.2.

^{1}H NMR (400 MHz, Chloroform-*d*) δ 7.08 (s, 2H), 6.97 (s, 2H), 4.34 (t, *J* = 4.8 Hz, 4H), 3.97 (dd, *J* = 5.9, 4.0 Hz, 4H), 3.84 – 3.77 (m, 4H), 3.80 – 3.65 (m, 6H), 3.70 – 3.59 (m, 8H), 3.61 (d, *J* = 3.0 Hz, 2H), 3.55 – 3.48 (m, 4H), 3.35 (s, 6H).

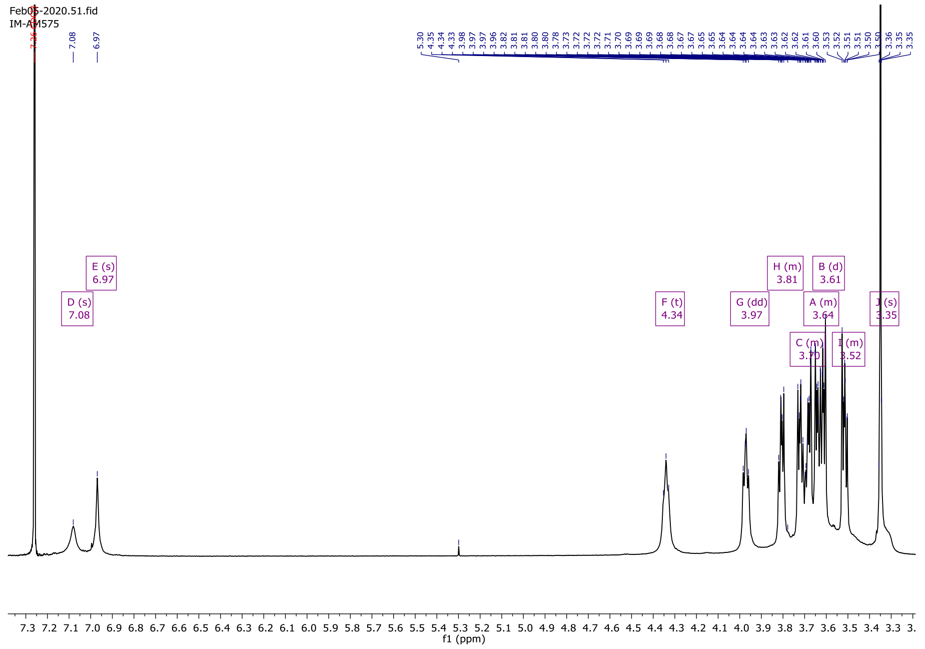

Figure S31. ^{1}H NMR in $CDCl_3$.

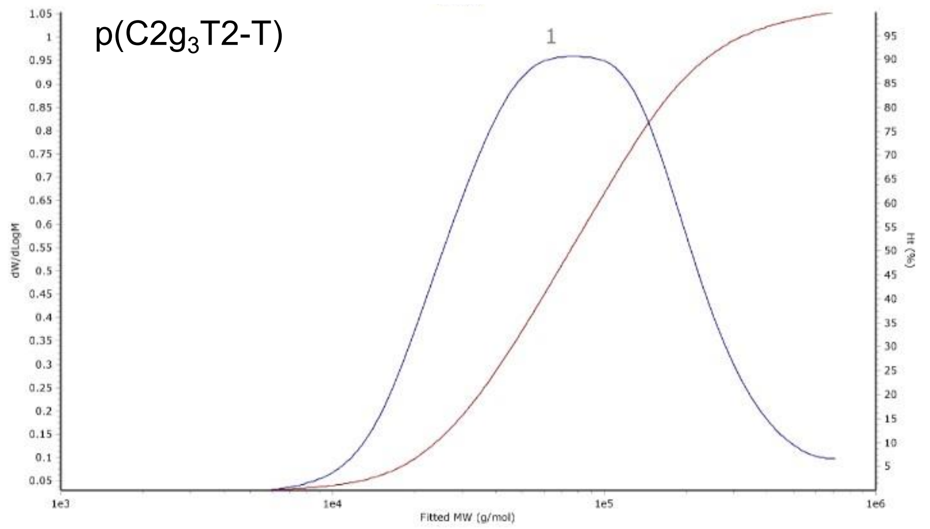

Figure S32. GPC spectra. M_n, M_w, and $Đ$ (M_w/M_n) were determined by GPC using low-D (<1.10) polystyrene standards and DMF as the eluent at 40 °C.

10.6. Synthesis of 4-(thiophen-3-yloxy)butan-1-ol

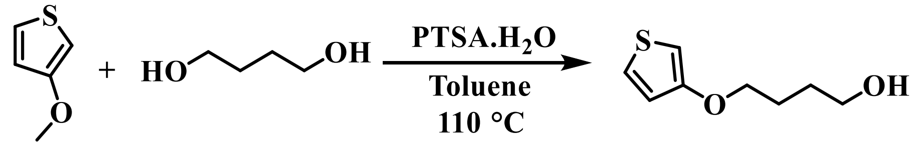

3-Methoxythiophene (17.5 mL, 175.4 mmol, 1.0 eq.) was dissolved in 250 mL of toluene, inside a 2-neck 500 mL RBF. 1,4-Butanediol (23.3 mL, 263.2 mmol, 1.5 eq.) was added dropwise prior to the addition of *p*-toluenesulfonic acid monohydrate (3.3 g, 17.5 mmol, 0.1 eq.) in a single portion. The reaction was heated to reflux (110 °C) and stirred for 90 minutes. Upon cooling to room temperature, the mixture was poured into water, the organic layer was extracted into DCM, separated and dried over $MgSO_4$. After the solvent was removed, under reduced pressure, the crude material was purified by column chromatography, on silica gel, using DCM as the eluent. Product fractions were consolidated to afford the title compound (11.55 g, 38%).

^{1}H NMR (400 MHz, Chloroform-*d*) δ 7.21 – 7.13 (m, 1H), 6.74 (dd, *J* = 5.2, 1.5 Hz, 1H), 6.24 (dd, *J* = 3.1, 1.5 Hz, 1H), 3.98 (td, *J* = 6.2, 0.7 Hz, 2H), 3.71 (td, *J* = 6.3, 1.0 Hz, 2H), 1.93 – 1.78 (m, 3H), 1.82 – 1.67 (m, 2H).

^{13}C NMR (101 MHz, Chloroform-*d*) δ 157.83, 124.76, 119.53, 97.37, 70.07, 62.60, 29.55, 25.89.

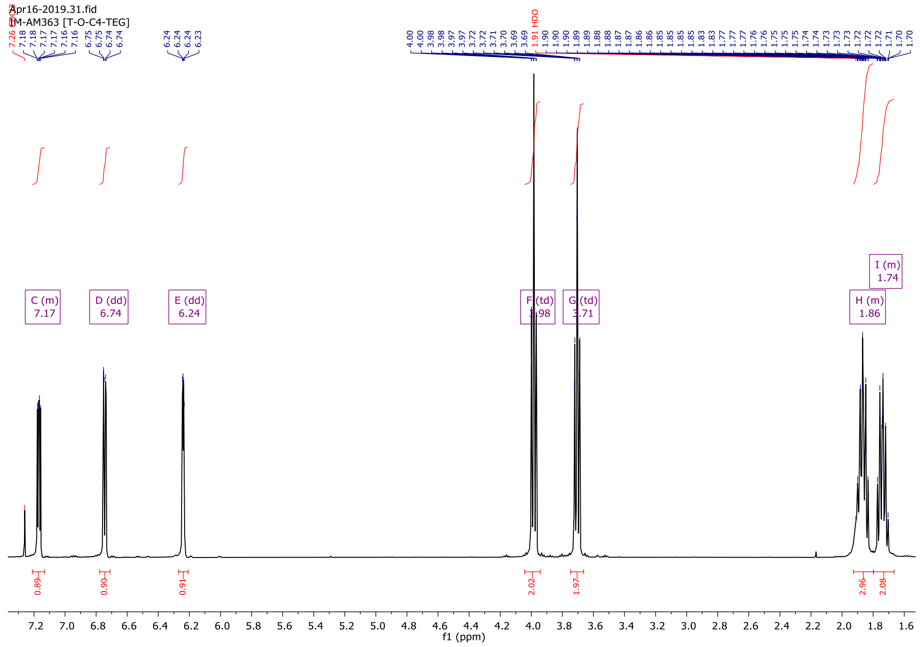

Figure S33. ^{1}H NMR in $CDCl_3$.

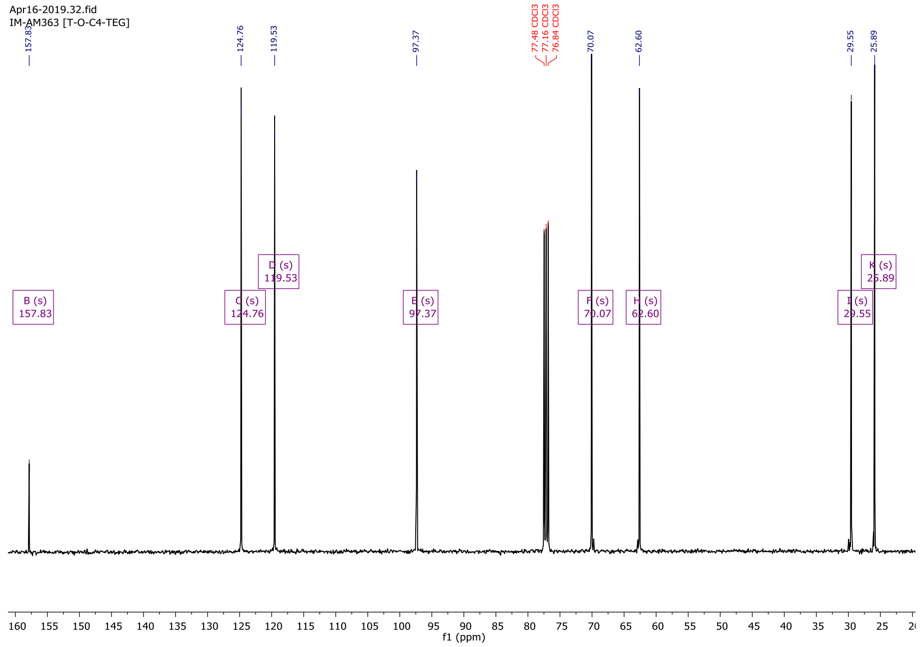

Figure S34. ^{13}C NMR in $CDCl_3$.

10.7. Synthesis of 4-(thiophen-3-yloxy)butyl 4-methylbenzenesulfonate

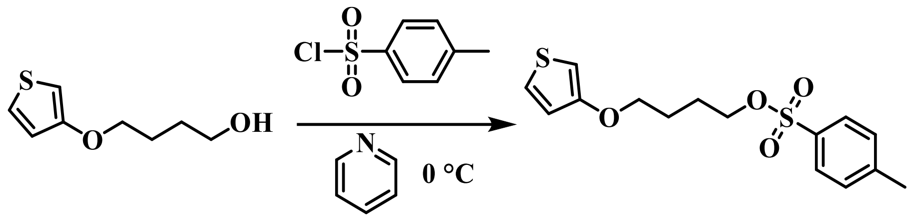

4-(thiophen-3-yloxy)butan-1-ol (11.55 g, 67.1 mmol, 1.0 eq.) was dissolved in 30 mL pyridine and cooled to 0 °C. 4-Toluenesulfonyl chloride (15.4 g, 80.6 mmol, 1.2 eq.) was added, in a single portion, and the reaction was stirred vigorously for 20 minutes, during which the solution turned an intense yellow colour. The reaction was stored in the refrigerator overnight, afterwards 30 mL 2 M HCl was added and the solution stirred rapidly for 30 minutes. The mixture was poured into water, extracted with DCM and the organic layer was dried over Na_2SO_4 prior to removing the solvent under reduced pressure. The product was dried under vacuum and used in the next step without further purification. (18.41 g, 84%).

^{1}H NMR (400 MHz, Chloroform-*d*) δ 7.79 (d, *J* = 8.3 Hz, 2H), 7.34 (d, *J* = 8.2 Hz, 2H), 7.16 (dd, *J* = 5.2, 3.2 Hz, 1H), 6.69 (dd, *J* = 5.2, 1.5 Hz, 1H), 6.17 (dd, *J* = 3.1, 1.6 Hz, 1H), 4.10 (t, *J* = 6.0 Hz, 2H), 3.89 (t, *J* = 5.8 Hz, 2H), 2.44 (s, 3H), 1.82 (ttd, *J* = 11.7, 6.1, 5.6, 2.3 Hz, 4H).

^{13}C NMR (101 MHz, Chloroform-*d*) δ 157.75, 144.91, 133.22, 133.22, 129.99, 128.03, 124.82, 119.49, 97.36, 70.22, 69.12, 25.85, 25.41, 21.78.

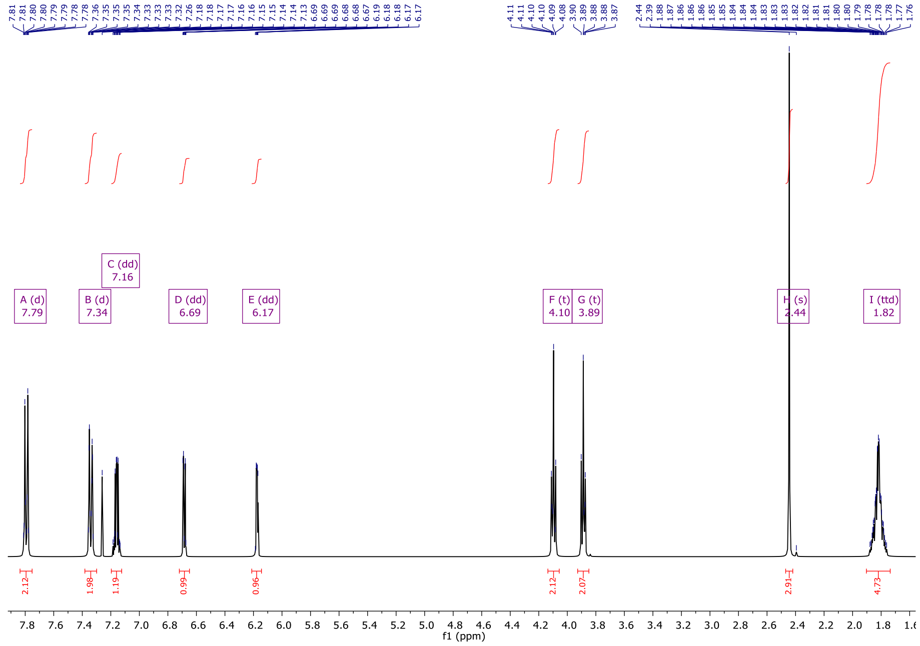

Figure S35. ^{1}H NMR in $CDCl_3$.

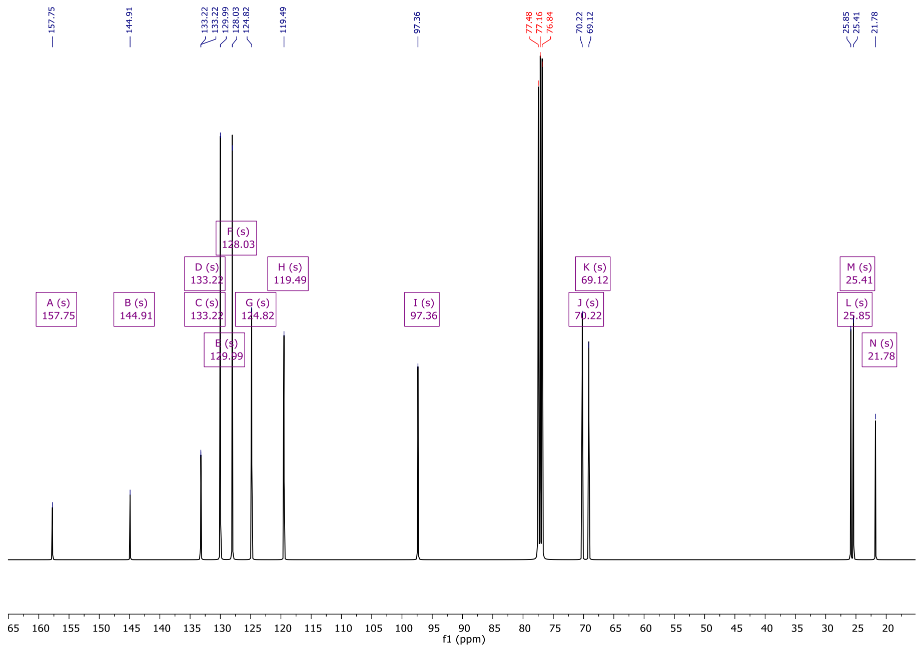

Figure S36. ^{13}C NMR in $CDCl_3$.

10.8. Synthesis of 15-(thiophen-3-yloxy)-2,5,8,11-tetraoxapentadecane

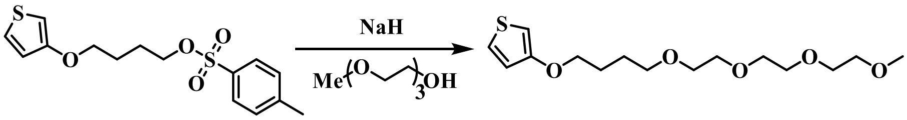

To an oven dried 2-neck 250 mL RBF was added sodium hydride (60% in mineral oil) (6.62 g, 165.4 mmol, 3.0 eq.), dissolved in dry THF (50 mL) at 0 °C, under a Nitrogen atmosphere. Triethylene glycol monomethyl ether (26.5 mL, 165.4 mmol, 3.0 eq.) was added dropwise and the mixture was stirred for 30 minutes until effervescence ceased. 4-(thiophen-3-yloxy)butyl 4-methylbenzenesulfonate (18 g, 55.14 mmol, 1.0 eq.) dissolved in 30 mL THF was added dropwise, the reaction was stirred overnight. The mixture was then poured into water, washed multiple times before being extracted into DCM and dried over $MgSO_4$. The crude residue was purified via column chromatography, on silica gel, using hexane:ethyl acetate (1:1) as the eluent system, product fractions were consolidated to afford a yellow oil (4.73 g, 27%).

^{1}H NMR (400 MHz, Chloroform-*d*) δ 7.15 (dd, *J* = 5.2, 3.1 Hz, 1H), 6.72 (dd, *J* = 5.2, 1.6 Hz, 1H), 6.21 (dd, *J* = 3.1, 1.5 Hz, 1H), 3.95 (t, *J* = 6.3 Hz, 2H), 3.73 – 3.60 (m, 10H), 3.63 – 3.45 (m, 4H), 3.36 (s, 3H), 1.89 – 1.66 (m, 4H).

^{13}C NMR (101 MHz, Chloroform-*d*) δ 151.81, 121.67, 115.99, 71.95, 71.61, 70.93, 70.63, 70.53, 70.10, 59.04, 26.51, 26.27.

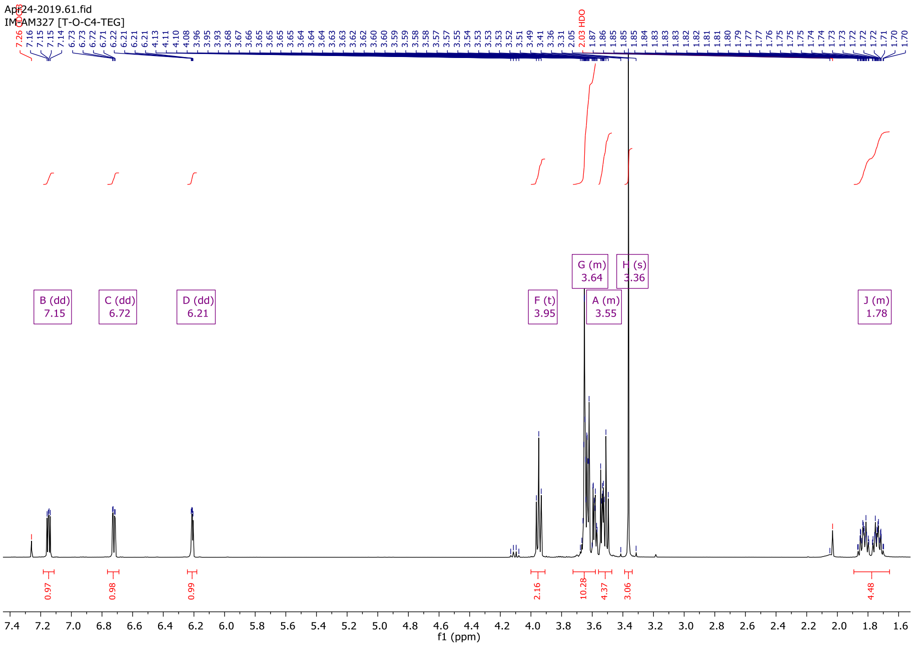

Figure S37. ^{1}H NMR in $CDCl_3$.

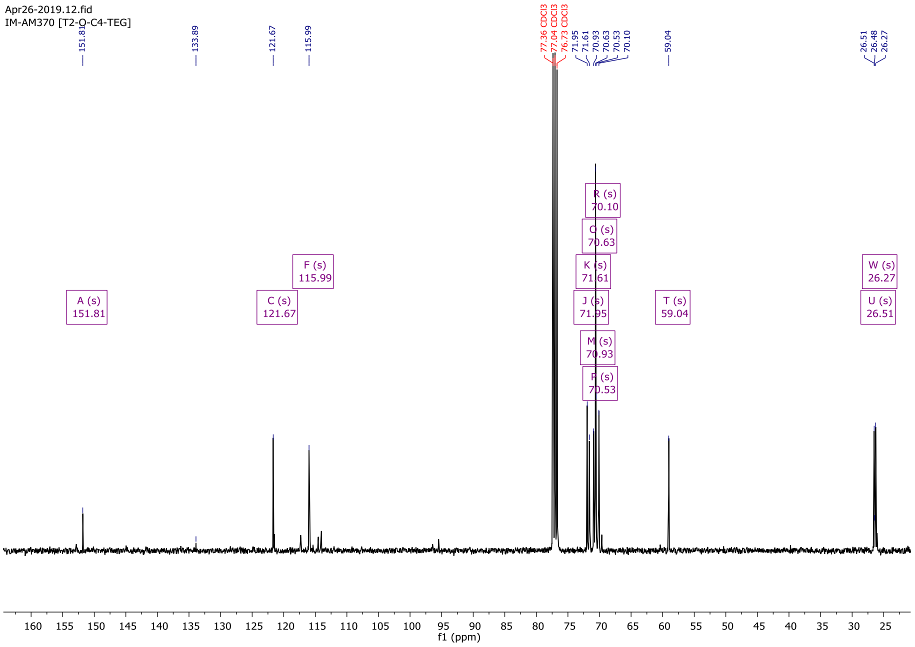

Figure S38. ^{13}C NMR in $CDCl_3$.

10.9. Synthesis of 15-((2-bromothiophen-3-yl)oxy)-2,5,8,11-tetraoxapentadecane

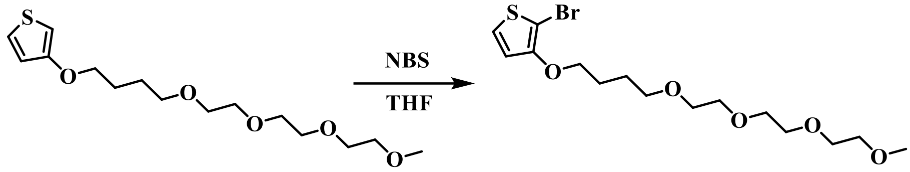

15-(thiophen-3-yloxy)-2,5,8,11-tetraoxapentadecane (4.7 g, 14.8 mmol, 1.0 eq.) was added to an oven dried 250 mL 2-neck RBF, dissolved in 100 mL dry THF. Cooled to 0 °C, in the dark prior to the portion wise addition of N-bromosuccinimide (4 × 700 mg, 15.01 mmol, 1.02 eq.) leaving approximately 4 minutes between each addition. After stirring for 90 minutes the reaction was poured into water, washed with saturated sodium bicarbonate solution and quenched with sodium metabisulphite. The aqueous phase was extracted three times with DCM, the organic layers were combined, dried over Na_2SO_4 and solvent removed under reduced pressure. The crude was purified via column chromatography, on silica gel, using hexane:ethyl acetate (1:1) (v/v) as the eluent system. Product fractions were consolidated to yield a pale-yellow oil (5.79 g, 97%).

^{1}H NMR (400 MHz, Chloroform-*d*) δ 7.18 (d, *J* = 6.0 Hz, 1H), 6.73 (d, *J* = 5.9 Hz, 1H), 4.05 (t, *J* = 6.2 Hz, 2H), 3.70 – 3.54 (m, 10H), 3.57 – 3.47 (m, 4H), 3.37 (s, 3H), 1.88 – 1.69 (m, 4H).

^{13}C NMR (101 MHz, Chloroform-*d*) δ 154.55, 124.29, 117.58, 91.68, 72.05, 72.00, 70.98, 70.73, 70.63, 70.21, 59.15, 26.42, 26.15.

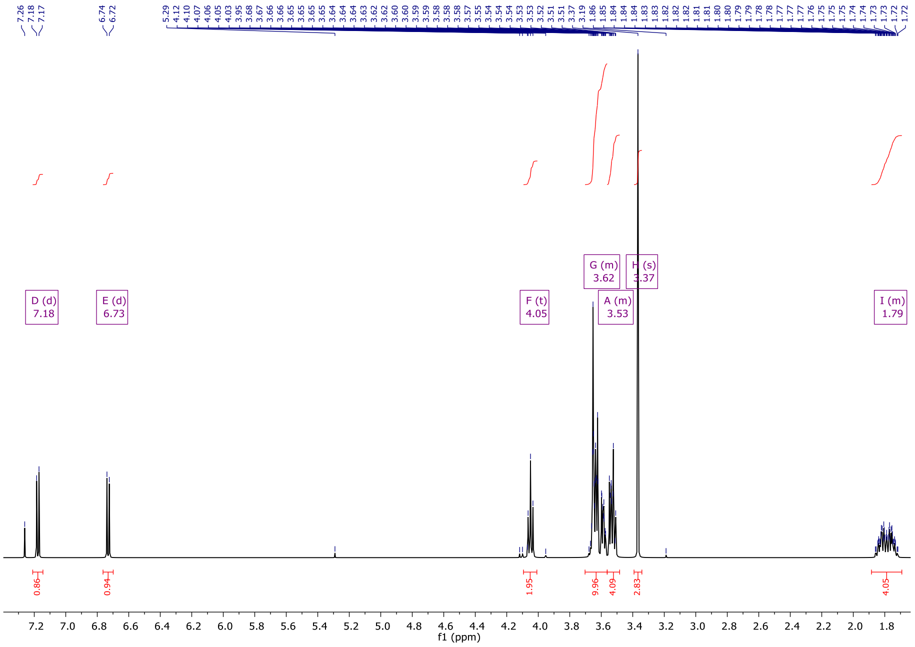

Figure S39. ^{1}H NMR in $CDCl_3$.

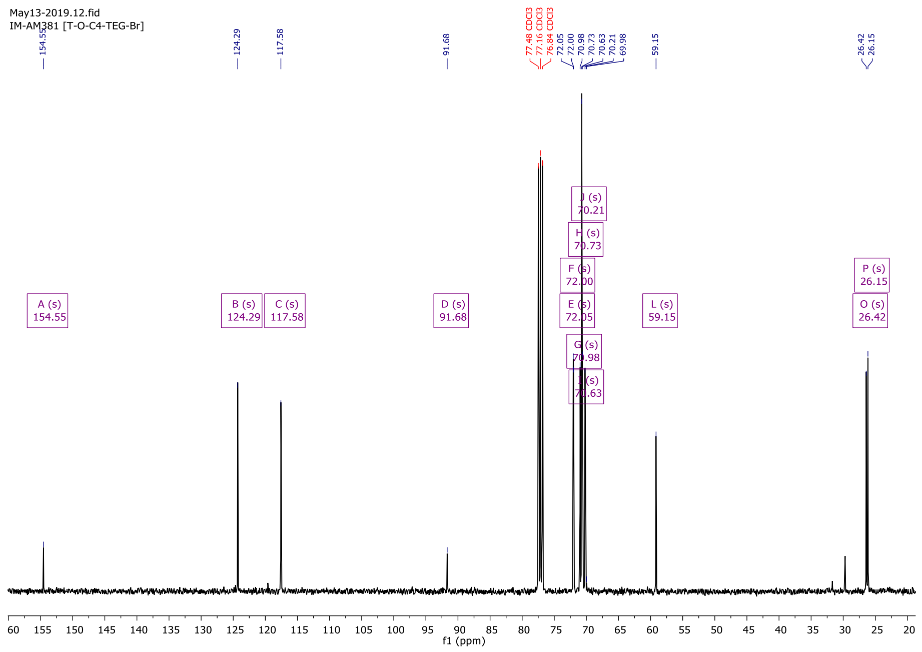

Figure S40. ^{13}C NMR in $CDCl_3$.

10.10. Synthesis of 3,3'-bis((2,5,8,11-tetraoxapentadecan-15-yl)oxy)-2,2'-bithiophene

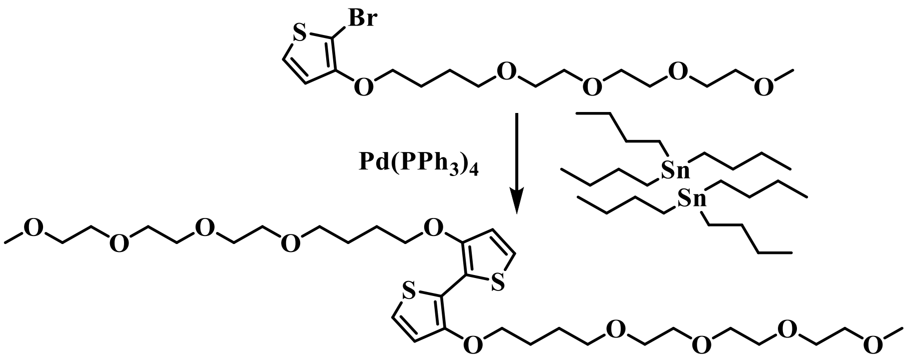

15-(thiophen-3-yloxy)-2,5,8,11-tetraoxapentadecane (4.7 g, 11.8 mmol, 1.0 eq.) was added to an oven dried 2-neck 250 mL RBF and dissolved in a 1:1 mixture of anhydrous chlorobenzene (37 mL) and dry DMF (37 mL), under a nitrogen atmosphere. Bis(tributyltin) (3.73 mL, 5.91 mmol, 0.5 eq.) was added dropwise, followed by the addition of $Pd(PPh_3)_4$ (1.71 g, 1.18 mmol, 0.01 eq.). The reaction was stirred for 48 hours at 100 °C, upon cooling to room temperature the mixture was passed through a short silica plug, eluting with ethyl acetate. The solvent was removed under reduced pressure and the crude was purified by column chromatography, on silica gel, using ethyl acetate as the eluent system. Product fractions were consolidated to yield a yellow oil which solidified at reduced temperature (3.37 g, 58%).

^{1}H NMR (400 MHz, Chloroform-*d*) δ 7.07 (d, J = 5.5 Hz, 2H), 6.83 (d, J = 5.6 Hz, 2H), 4.12 (t, J = 6.2 Hz, 4H), 3.69 – 3.50 (m, 28H), 3.37 (s, 6H), 1.96 – 1.84 (m, 4H), 1.84 (ddd, J = 8.1, 4.9, 1.6 Hz, 4H).

^{13}C NMR (101 MHz, Chloroform-*d*) δ 151.93, 121.79, 116.11, 72.06, 71.73, 71.05, 70.75, 70.65, 70.22, 59.16, 26.63, 26.39.

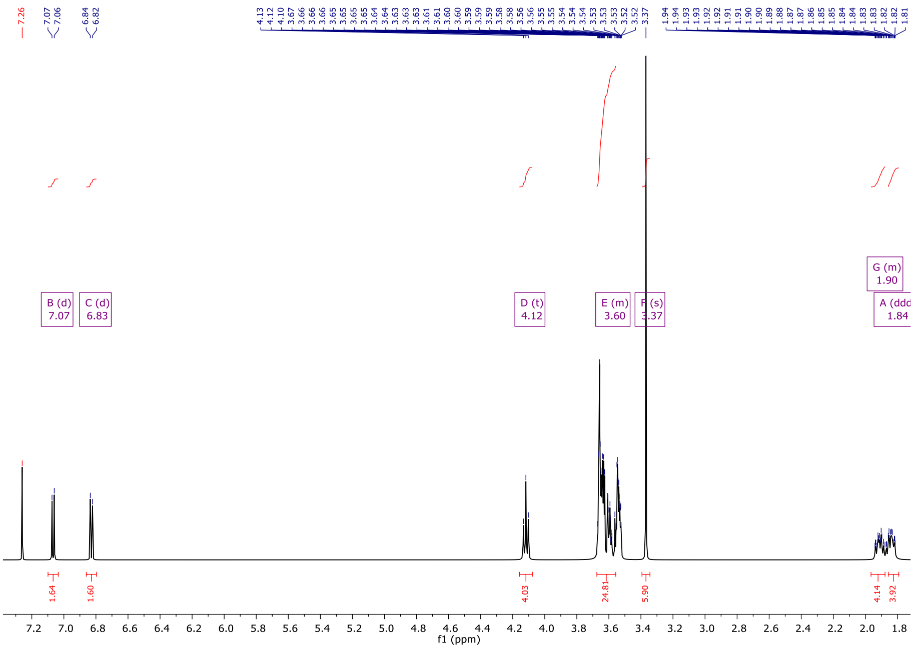

Figure S41. ^{1}H NMR in $CDCl_3$.

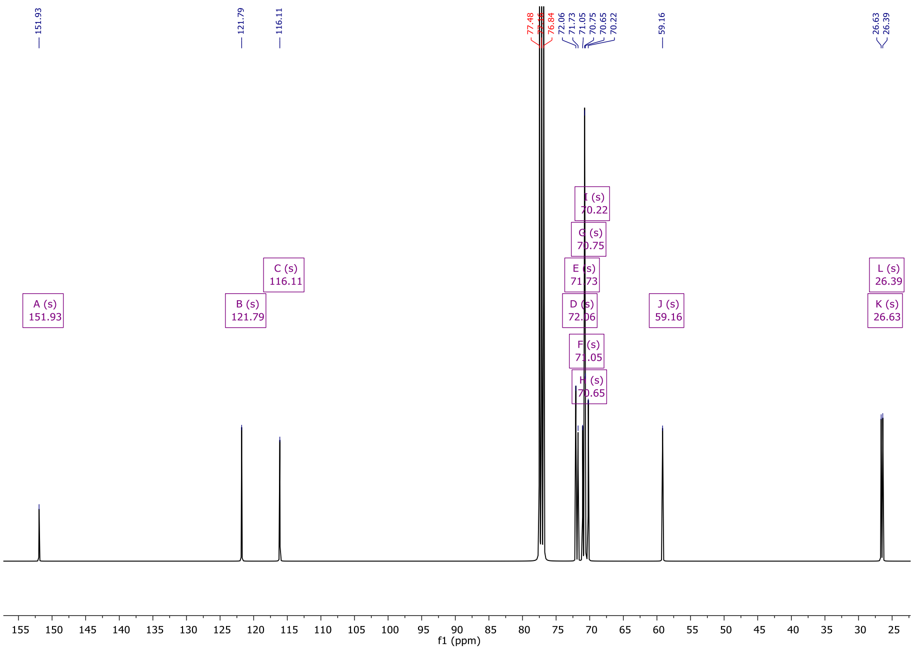

Figure S42. ^{13}C NMR in $CDCl_3$.

10.11. Synthesis of 15,15'-((5,5'-dibromo-[2,2'-bithiophene]-3,3'-diyl)bis(oxy))bis(2,5,8,11-tetraoxapentadecane)

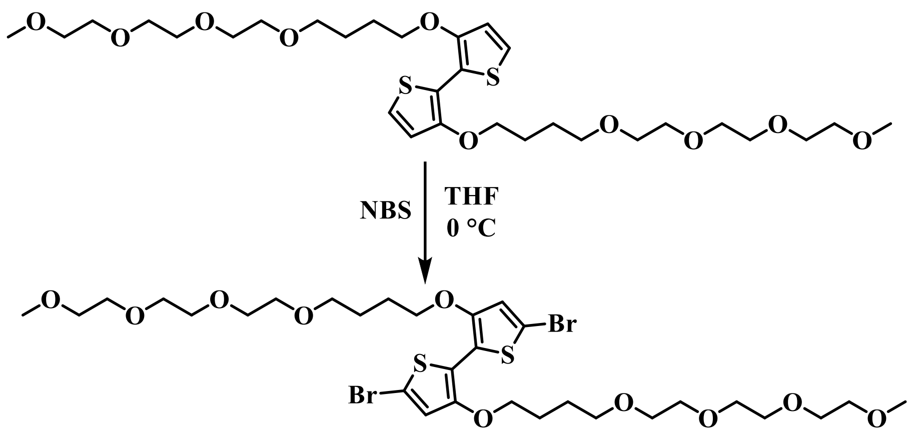

3,3'-bis((2,5,8,11-tetraoxapentadecan-15-yl)oxy)-2,2'-bithiophene (2 g, 3.15 mmol, 1.0 eq.) was dissolved in 50 mL anhydrous THF and cooled to 0 °C, under a Nitrogen atmosphere. The reaction was covered and kept in the dark, N-bromosuccinimide (1.15 g, 6.46 mmol, 2.05 eq.) was added in four equal portions, leaving 4 minutes between each addition. After stirring for 30 minutes the reaction was poured into water, washed with saturated sodium bicarbonate solution and quenched with sodium metabisulphite. The aqueous phase was extracted into DCM three times, the organic layers were combined and dried over Na_2SO_4, solvent removed under reduced pressure. The crude product was purified by column chromatography, on silica gel, using ethyl acetate as the eluent system to afford a yellow oil, which solidified at low temperatures (2.4 g, 96%).

^{1}H NMR (400 MHz, Chloroform-*d*) δ 6.81 (s, 2H), 4.07 (t, *J* = 6.4 Hz, 4H), 3.72 – 3.56 (m, 20H), 3.59 – 3.50 (m, 8H), 3.37 (s, 6H), 1.96 – 1.84 (m, 4H), 1.79 (dqd, *J* = 12.1, 6.0, 2.4 Hz, 4H).

^{13}C NMR (101 MHz, Chloroform-*d*) δ 239.55, 223.91, 176.83, 171.13, 150.23, 119.03, 115.02, 109.85, 72.06, 71.95, 70.77, 70.64, 70.53, 70.11, 60.39, 59.03, 29.58, 26.41, 26.10, 26.05, -15.85.

Mass (MALDI-ToF): 792.7 $[M+H]^+$ (calc. 792.1 $C_{30}H_{48}Br_2O_{10}S_2$).

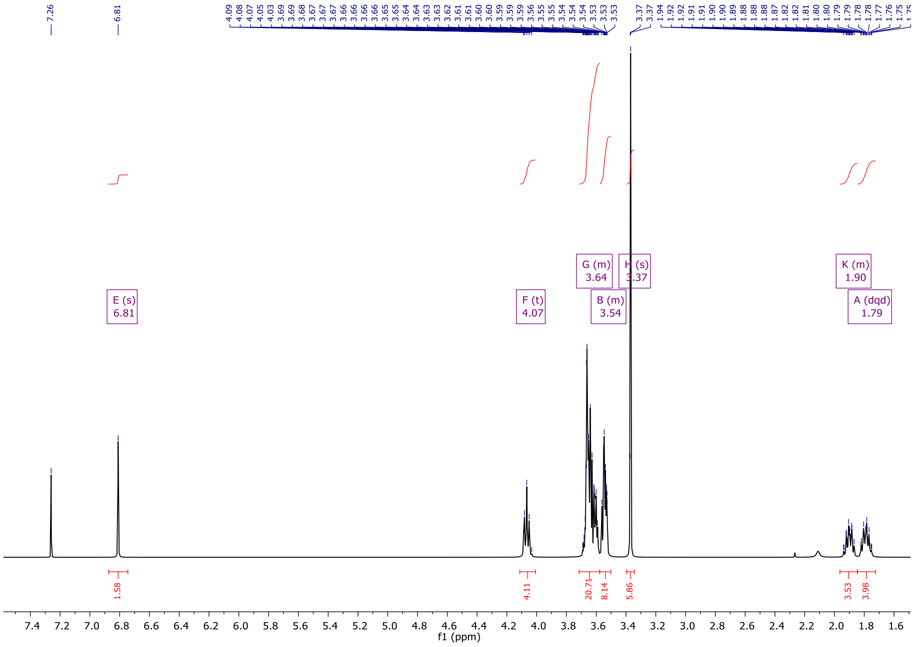

Figure S43. ^{1}H NMR in $CDCl_3$.

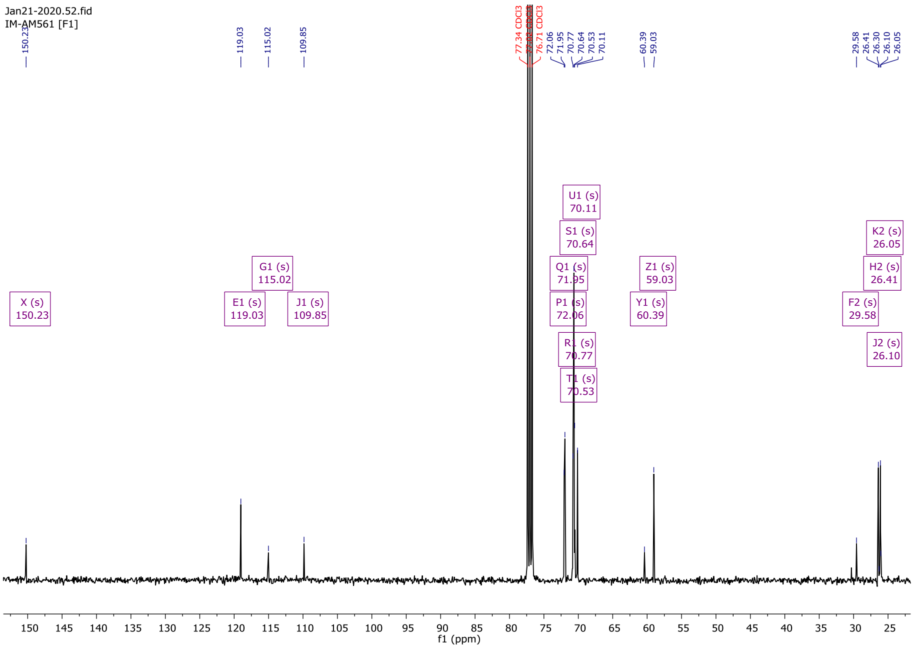

Figure S44. ^{13}C NMR in $CDCl_3$.

10.12. Synthesis of Polymer p(C4g3T2-TT)

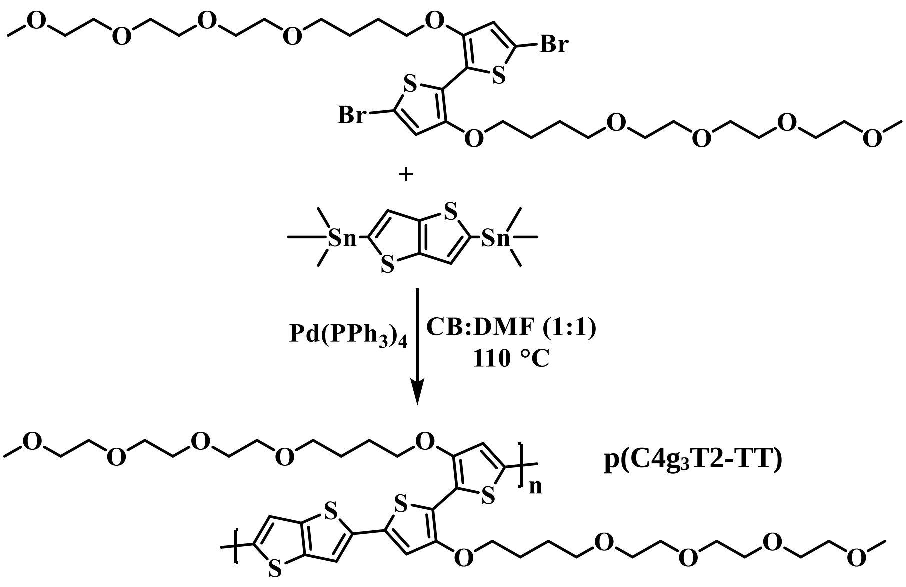

An oven dried 5 mL microwave vial was charged with 15,15'-((5,5'-dibromo-[2,2'-bithiophene]-3,3'-diyl)bis(oxy))bis(2,5,8,11-tetraoxapentadecane) (200 mg, 0.25 mmol, 1.0 eq.), 2,5-bis(trimethylstannyl)thieno[3,2-*b*]thiophene (117.6 mg, 0.25 mmol, 1.0 eq.) and $Pd(PPh_3)_4$ (5.80 mg, 0.005 mmol, 0.02 eq.). Cap was sealed and the vial was purged with Nitrogen for 5 minutes prior to the addition of anhydrous DMF (1.70 mL) and anhydrous chlorobenzene (1.70 mL). The reaction was stirred overnight at 110 °C then cooled to room temperature, upon which the solution formed a purple gel. Crude polymer was precipitated into 100 mL methanol and subsequently filtered into a thimble. Purification was conducted via Soxhlet extraction, washing with hexane, methanol, acetone, ethyl acetate and chloroform (in that order). The chloroform fraction was collected, solvent removed under reduced pressure and re-precipitated into 100 mL methanol, filtered to yield a blue metallic film (126 mg, 63%). GPC (DMF, 40 °C): M_n 130.5 kDa, M_w 268.6 kDa, $Đ$ 2.1.

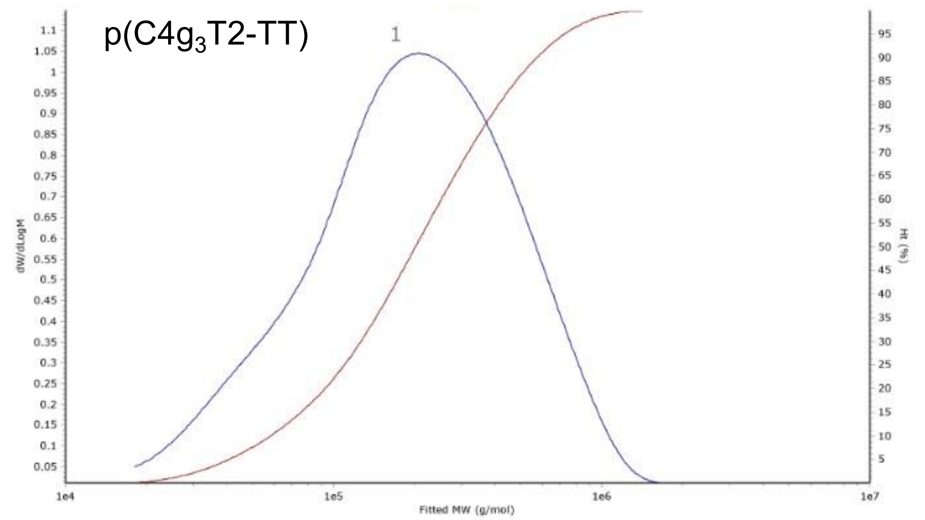

Figure S45. GPC spectra. M_n, M_w, and *Đ* (Mw/M_n) were determined by GPC using low-D (<1.10) polystyrene standards and DMF as the eluent at 40 °C.

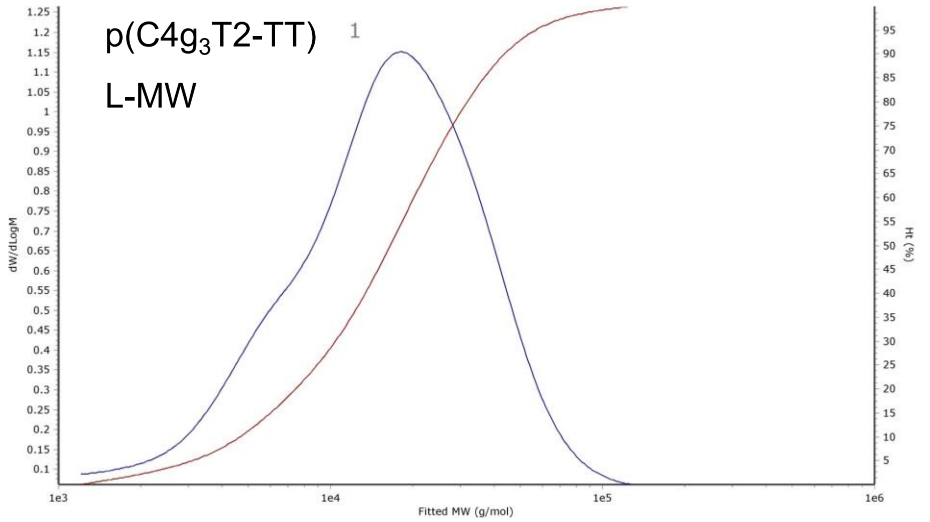

Figure S46. GPC spectra. M_n, M_w, and *Đ* (M_w/M_n) were determined by GPC using low-D (<1.10) polystyrene standards and DMF as the eluent at 40 °C.

10.13. Synthesis of Polymer p(C4g$_3$T2-T)

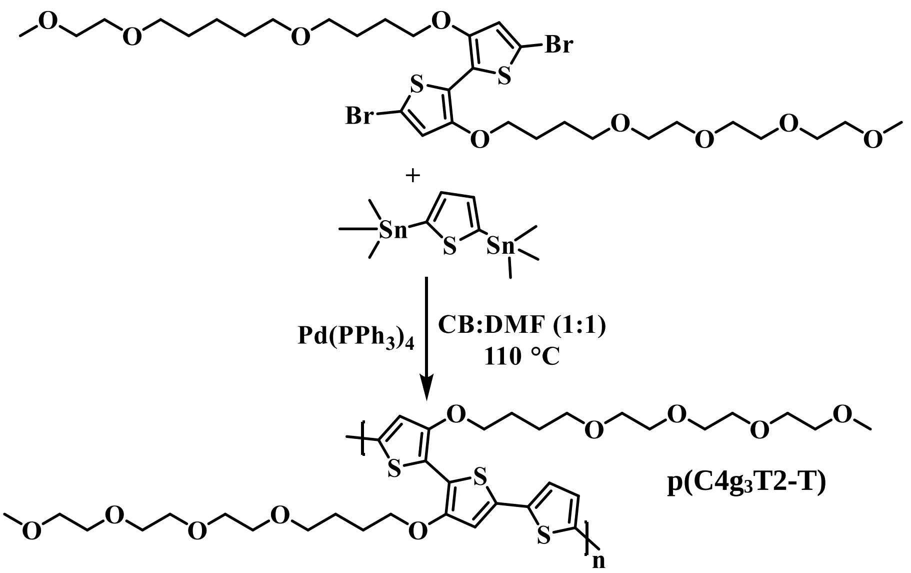

An oven dried 5 mL microwave vial was charged with 15,15'-((5,5'-dibromo-[2,2'-bithiophene]-3,3'-diyl)bis(oxy))bis(2,5,8,11-tetraoxapentadecane) (200 mg, 0.25 mmol, 1.0 eq.), 2,5-bis(trimethylstannyl)thiophene (103.4 mg, 0.25 mmol, 1.0 eq.) and $Pd(PPh_3)_4$ (5.80 mg, 0.005 mmol, 0.02 eq.). Cap was sealed and the vial was purged with Nitrogen for 5 minutes prior to the addition of anhydrous DMF (1.70 mL) and anhydrous chlorobenzene (1.70 mL). The reaction was stirred overnight at 110 °C then cooled to room temperature, upon which the solution formed a purple gel. Crude polymer was precipitated into 100 mL methanol and subsequently filtered into a thimble. Purification was conducted via Soxhlet extraction, washing with hexane, methanol, acetone, ethyl acetate and chloroform (in that order). The chloroform fraction was collected, solvent removed under reduced pressure and re-precipitated into 100 mL methanol, filtered to yield a blue metallic film (101 mg, 51%). GPC (DMF, 40 °C): M_n 19.9 kDa, M_w 43.7 kDa, $Đ$ 2.2.

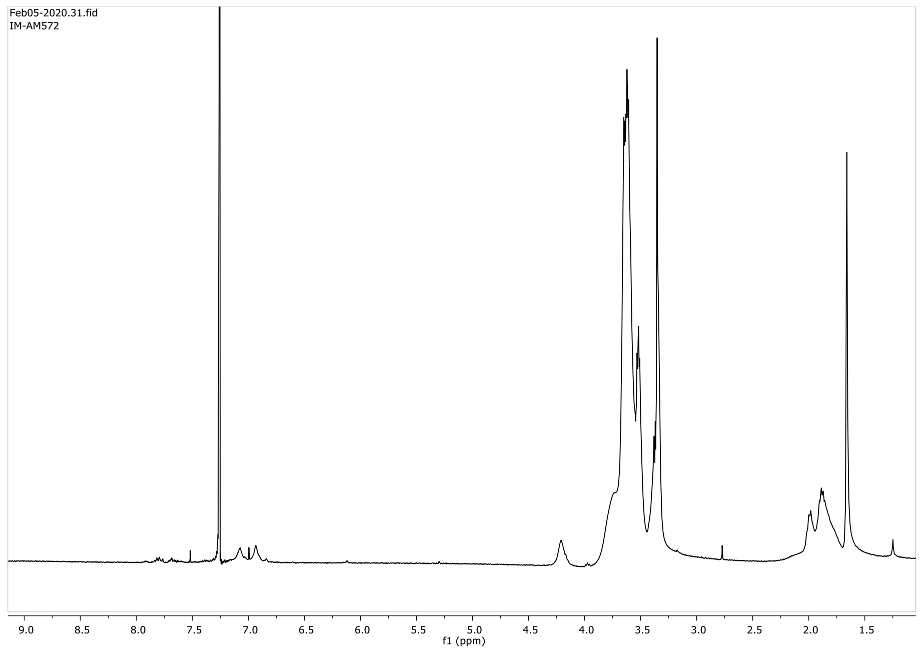

Figure S47. ^{1}H NMR in $CDCl_3$.

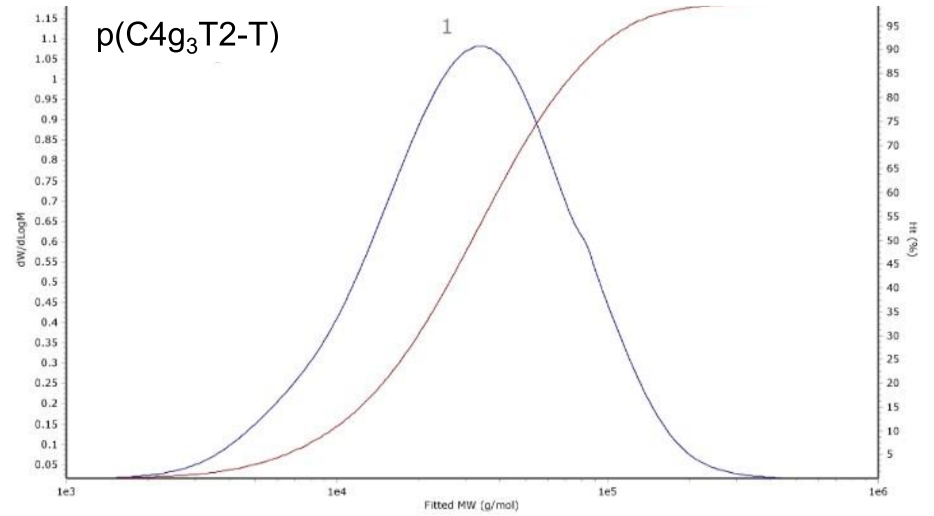

Figure S48. GPC spectra. M_n, M_w, and *Đ* (M_w/M_n) were determined by GPC using low-D (<1.10) polystyrene standards and DMF as the eluent at 40 °C.

10.14. Synthesis of 6-(thiophen-3-yloxy)hexan-1-ol

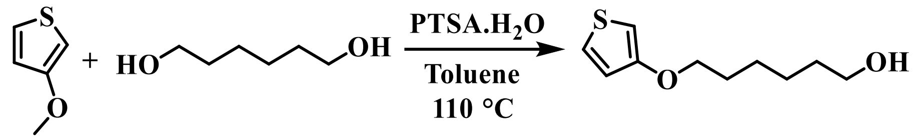

1,6-Hexanediol (33.6 g, 284.3 mmol, 1.5 eq.) was dissolved in 200 mL of toluene, *p*-toluenesulfonic acid monohydrate (4.15g, 18.9 mmol, 0.1 eq.) was added in a single portion. The mixture was heated to reflux (120 °C) prior to the addition of 3-methoxythiophene (25 g, 189.6 mmol, 1.0 eq.) and left to stir for 90 minutes. Upon cooling to room temperature, the reaction was poured into water and the aqueous layer was extracted three times with DCM. The organic layers were combined and dried over $MgSO_4$; solvent was removed under reduced pressure. The crude material was purified by column chromatography, on silica gel, using DCM as the eluent system. Product fractions were combined to afford the title compound (19.3 g, 44%).

^{1}H NMR (400 MHz, Chloroform-*d*) δ 7.20 – 7.13 (m, 1H), 6.75 (dd, *J* = 5.2, 1.4 Hz, 1H), 6.22 (dd, *J* = 3.2, 1.5 Hz, 1H), 3.94 (t, *J* = 6.5 Hz, 2H), 3.70 – 3.60 (m, 2H), 1.78 (p, *J* = 6.7 Hz, 2H), 1.66 – 1.54 (m, 2H), 1.57 – 1.36 (m, 4H).

^{13}C NMR (101 MHz, Chloroform-*d*) δ 158.07, 124.67, 119.61, 97.15, 70.20, 62.96, 32.77, 29.34, 26.00, 25.64.

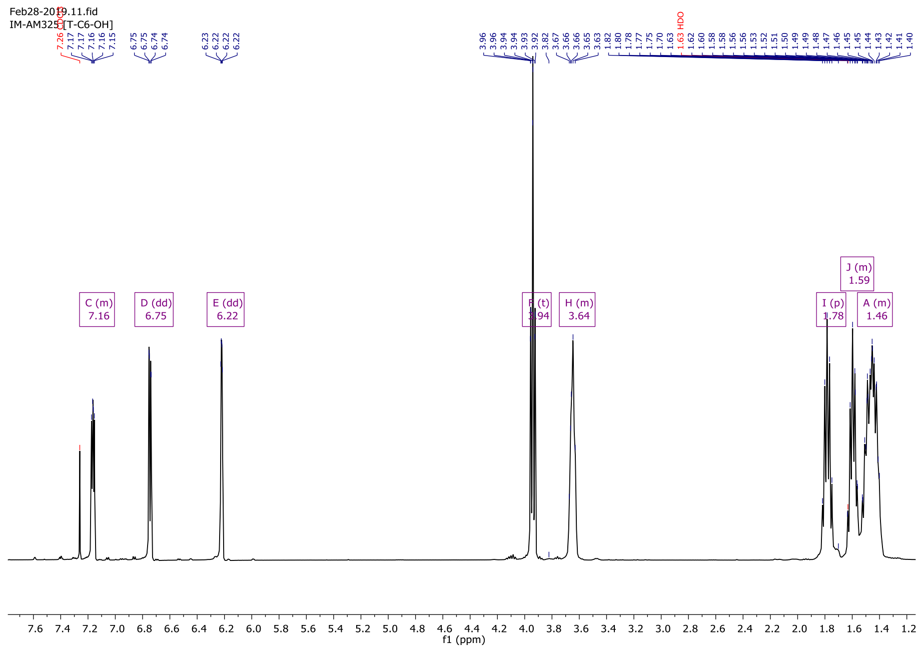

Figure S49. ^{1}H NMR in $CDCl_3$.

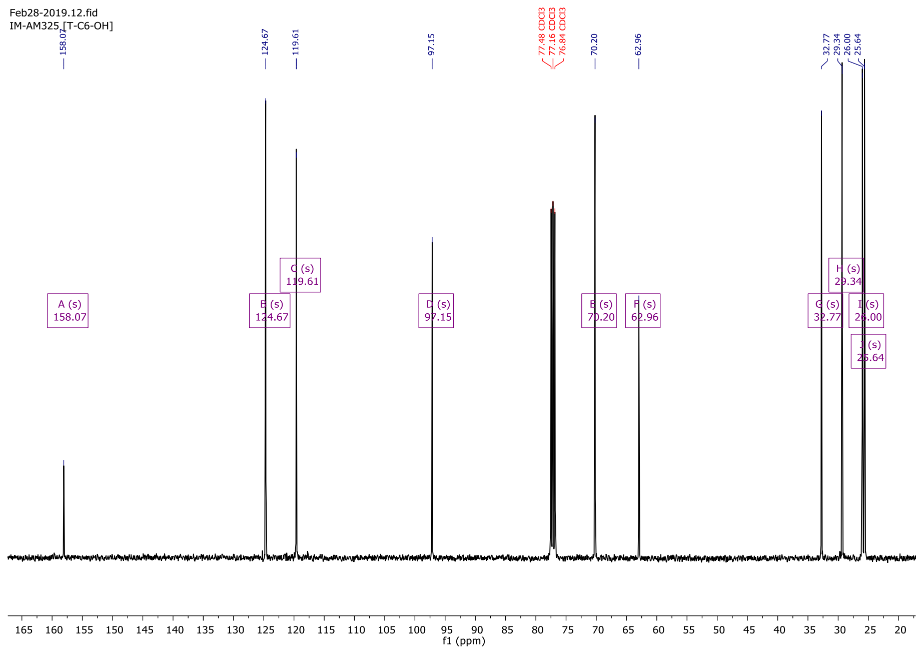

Figure S50. ^{13}C NMR in $CDCl_3$.

10.15. Synthesis of 6-(thiophen-3-yloxy)hexyl 4-methylbenzenesulfonate

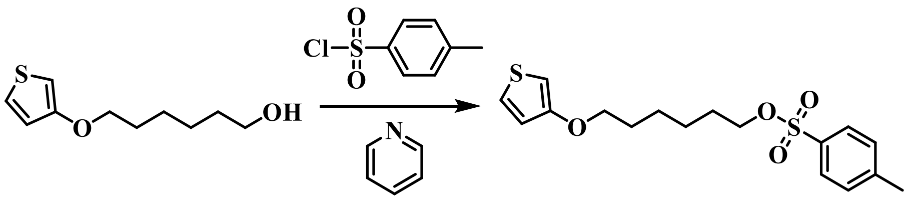

6-(thiophen-3-yloxy)hexan-1-ol (19.26 g, 96.2 mmol, 1.0 eq.) was dissolved in 30 mL pyridine and cooled to 0 °C. 4-Toluenesulfonyl chloride (22.0 g, 115.4 mmol, 1.2 eq.) was added, in a single portion, the reaction was stirred vigorously for 20 minutes, during which the solution turned an intense yellow colour. The reaction was then stored in the refrigerator overnight, afterwards 30 mL 2 M HCl was added and the solution stirred rapidly for 30 minutes. The mixture was poured into water, the aqueous phase was extracted with DCM three times, the organic layers were combined and dried over Na_2SO_4 prior to removing the solvent under reduced pressure. The product was dried under vacuum and used in the next step without further purification. (34 g, 99%).

10.16. Synthesis of 17-(thiophen-3-yloxy)-2,5,8,11-tetraoxaheptadecane

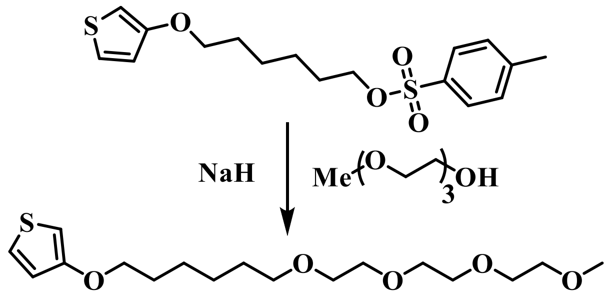

An oven dried 2-neck 250 mL RBF was charged with sodium hydride (60% in mineral oil) (11.51 g, 287.7 mmol, 3.0 eq.), dissolved in anhydrous THF (50 mL) cooled to 0 °C, under a Nitrogen atmosphere. Triethylene glycol monomethyl ether (46.1 mL, 287.7 mmol, 3.0 eq.) was added dropwise and the mixture was stirred for 30 minutes until effervescence ceased. 6-(thiophen-3-yloxy)hexyl 4-methylbenzenesulfonate (34 g, 95.9 mmol, 1.0 eq.) dissolved in 50 mL THF was added dropwise, the reaction was stirred overnight. The mixture was then poured into water, the aqueous layer was extracted three times with DCM, the organic layers were combined and dried over $MgSO_4$. The crude residue was purified via column chromatography, on silica gel, using hexane:ethyl acetate (1:1) (v/v) (Rf = 0.2) as the eluent system, product fractions were consolidated to afford a yellow oil (18.45 g, 55%).

^{1}H NMR (400 MHz, Chloroform-*d*) δ 7.15 (dd, *J* = 5.2, 3.1 Hz, 1H), 6.73 (dd, *J* = 5.2, 1.5 Hz, 1H), 6.21 (dd, *J* = 3.1, 1.5 Hz, 1H), 3.92 (t, *J* = 6.5 Hz, 2H), 3.71 – 3.48 (m, 12H), 3.45 (t, *J* = 6.7 Hz, 2H), 3.37 (s, 3H), 1.76 (dq, *J* = 8.1, 6.5 Hz, 2H), 1.66 – 1.54 (m, 2H), 1.52 – 1.32 (m, 4H).

^{13}C NMR (101 MHz, Chloroform-*d*) δ 157.77, 124.30, 119.29, 96.75, 71.73, 71.10, 70.41, 70.38, 70.31, 69.88, 58.82, 29.35, 29.00, 25.70.

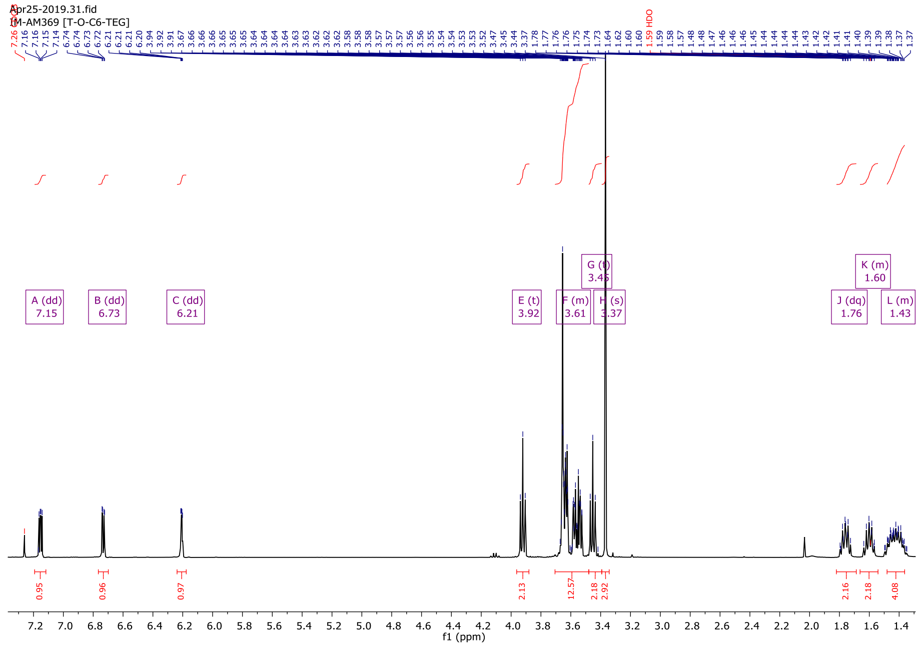

Figure S51. ^{1}H NMR in $CDCl_3$.

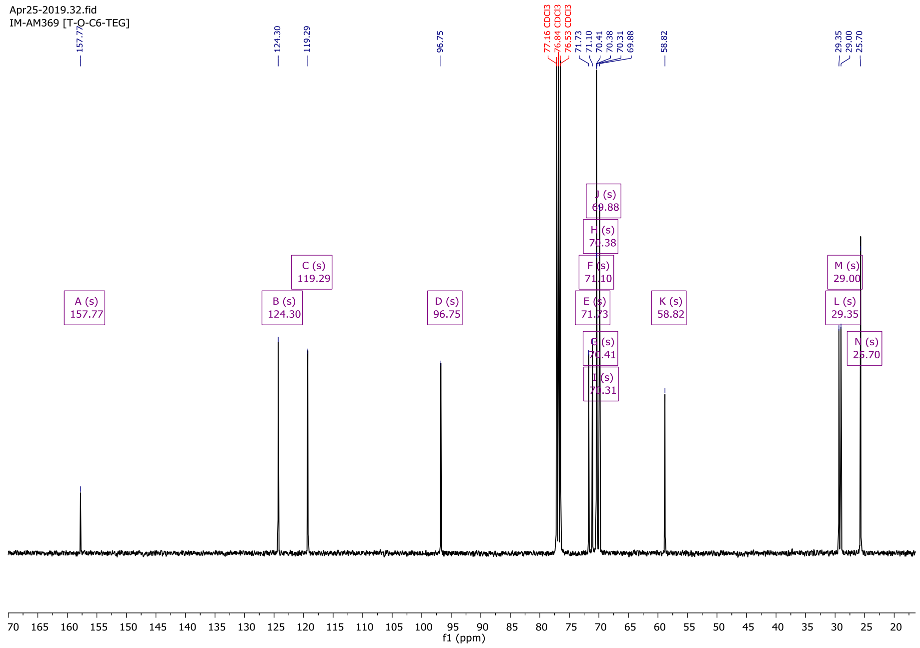

Figure S52. ^{13}C NMR in $CDCl_3$.

10.17. Synthesis of 17-((2-bromothiophen-3-yl)oxy)-2,5,8,11-tetraoxaheptadecane

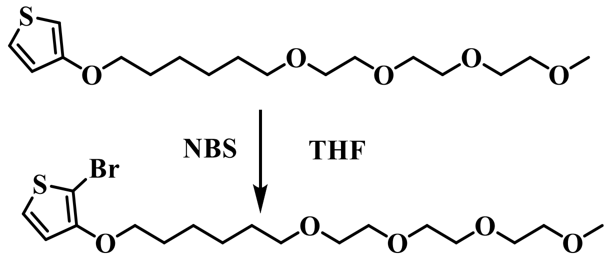

17-(thiophen-3-yloxy)-2,5,8,11-tetraoxaheptadecane (3.0 g, 8.66 mmol, 1.0 eq.) was added to an oven dried 250 mL 2-neck RBF, dissolved in 15 mL dry THF. Cooled to 0 °C, in the dark, prior to the portion wise addition of N-bromosuccinimide (4 × 394 mg, 8.83 mmol, 1.02 eq.) leaving approximately 4 minutes between each addition. After stirring for 60 minutes the reaction was poured into water, washed with saturated sodium bicarbonate solution and quenched with sodium metabisulphite. The aqueous phase was extracted three times with DCM, the organic layers were combined, dried over Na_2SO_4 and solvent removed under reduced pressure. The crude was purified via column chromatography, on silica gel, using hexane:ethyl acetate (1:1) (v/v) as the eluent system. Product fractions were consolidated to yield an orange oil (3.37 g, 91%).

^{1}H NMR (400 MHz, Chloroform-*d*) δ 7.18 (d, *J* = 6.0 Hz, 1H), 7.18 – 7.12 (m, 1H), 6.72 (d, *J* = 6.0 Hz, 1H), 4.02 (t, *J* = 6.5 Hz, 2H), 3.71 – 3.50 (m, 12H), 3.45 (t, *J* = 6.7 Hz, 2H), 3.37 (s, 3H), 1.82 – 1.69 (m, 2H), 1.66 – 1.54 (m, 2H), 1.53 – 1.33 (m, 4H).

^{13}C NMR (101 MHz, Chloroform-*d*) δ 154.61, 124.28, 117.62, 91.73, 72.22, 72.06, 71.43, 70.74, 70.71, 70.64, 70.20, 59.16, 29.66, 29.54, 25.95, 25.81.

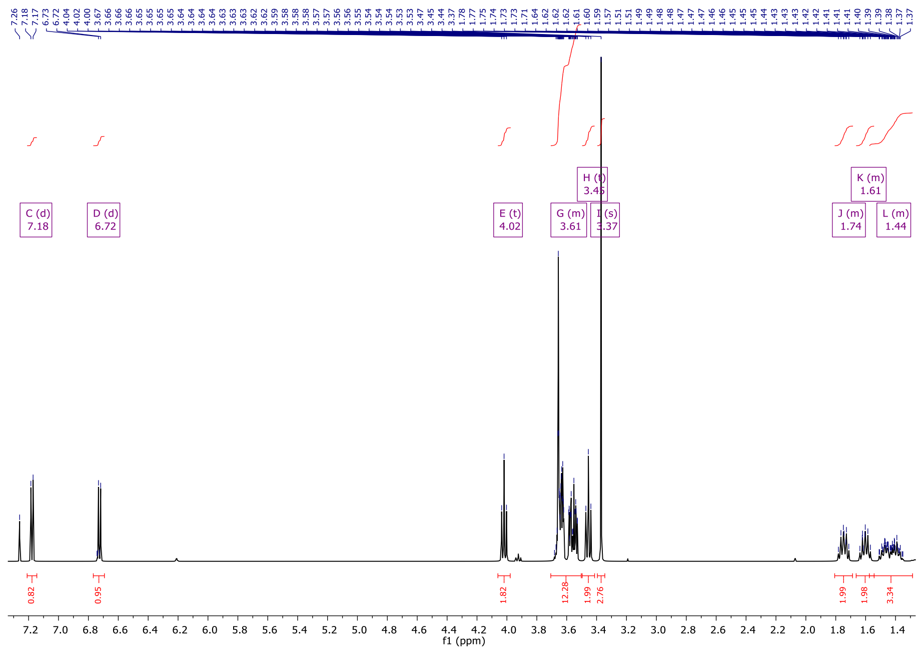

Figure S53. ^{1}H NMR in $CDCl_3$.

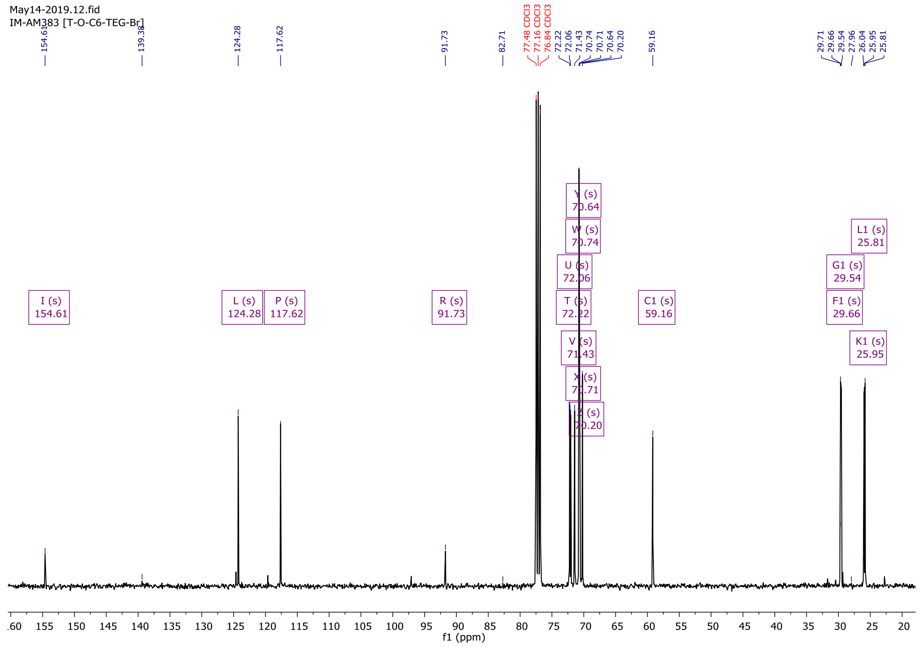

Figure S54. ^{13}C NMR in $CDCl_3$.

10.18. Synthesis of 3,3'-bis((2,5,8,11-tetraoxaheptadecan-17-yl)oxy)-2,2'-bithiophene

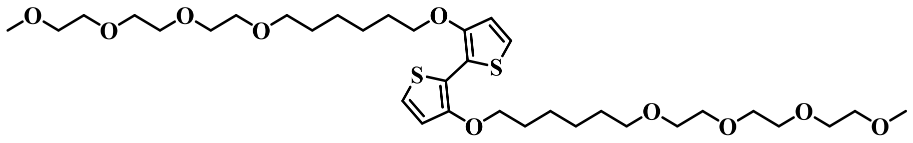

17-((2-bromothiophen-3-yl)oxy)-2,5,8,11-tetraoxaheptadecane (2.2 g, 5.17 mmol, 1.0 eq.) was added to an oven dried 2-neck 100 mL RBF and dissolved in a 1:1 mixture of anhydrous chlorobenzene (12 mL) and dry DMF (12 mL), under a Nitrogen atmosphere. Bis(tributyltin) (1.30 mL, 1.29 mmol, 0.5 eq.) was added dropwise, followed by the addition of $Pd(PPh_3)_4$ (299 mg, 0.129 mmol, 0.05 eq.). The reaction was stirred for 48 hours at 100 °C, upon cooling to room temperature the mixture was passed through a short silica plug, eluting with ethyl acetate. The solvent was removed under reduced pressure and the crude was purified by column chromatography, on silica gel, using ethyl acetate as the eluent system. Product fractions were consolidated to yield a yellow oil which solidified at reduced temperature (820 mg, 46%).

^{1}H NMR (400 MHz, Chloroform-*d*) δ 7.06 (d, *J* = 5.5 Hz, 2H), 6.82 (d, *J* = 5.7 Hz, 2H), 4.08 (t, *J* = 6.4 Hz, 4H), 3.69 – 3.57 (m, 16H), 3.61 – 3.51 (m, 8H), 3.46 (t, *J* = 6.6 Hz, 4H), 3.37 (s, 6H), 1.90 – 1.78 (m, 4H), 1.66 – 1.48 (m, 8H), 1.48 – 1.36 (m, 4H).

^{13}C NMR (101 MHz, Chloroform-*d*) δ 162.66, 151.96, 121.75, 116.09, 72.04, 71.91, 71.48, 70.63, 70.20, 59.16, 36.65, 31.58, 29.75, 29.67, 26.05, 25.96.

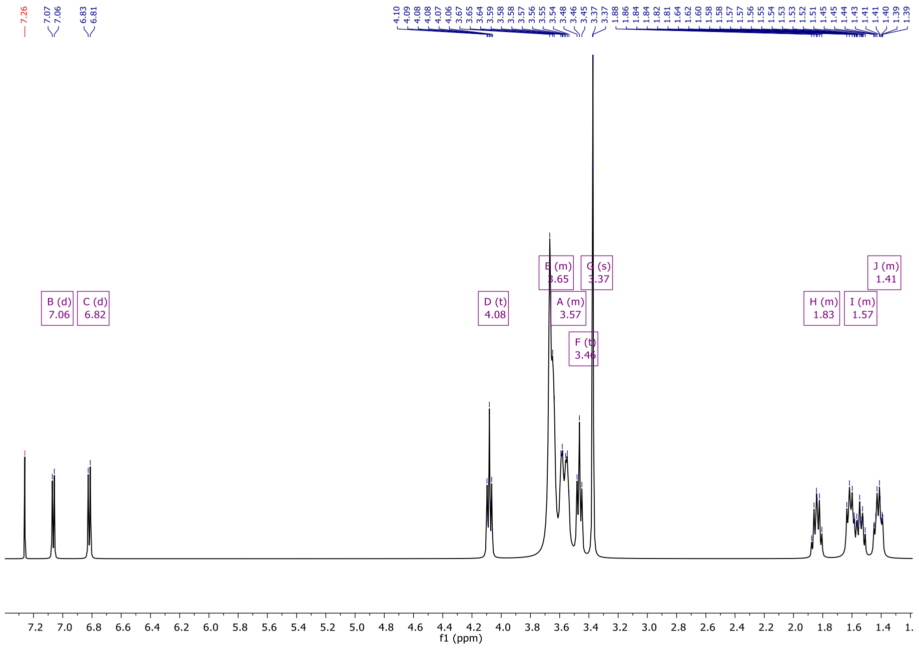

Figure S55. ^{1}H NMR in $CDCl_3$.

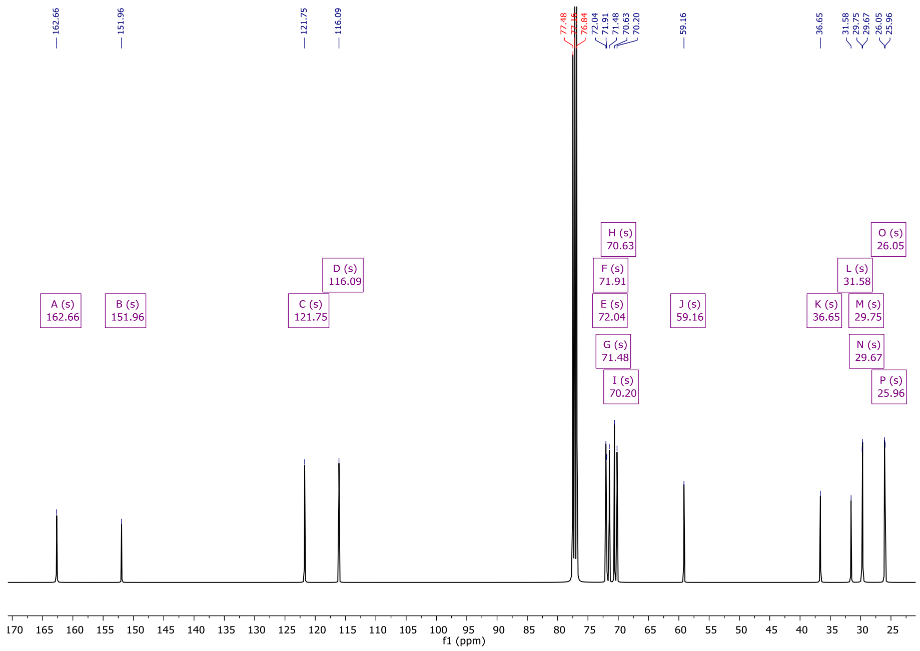

Figure S56. ^{13}C NMR in $CDCl_3$.

10.19. Synthesis of 17,17'-((5,5'-dibromo-[2,2'-bithiophene]-3,3'-diyl)bis(oxy))bis(2,5,8,11-tetraoxaheptadecane)

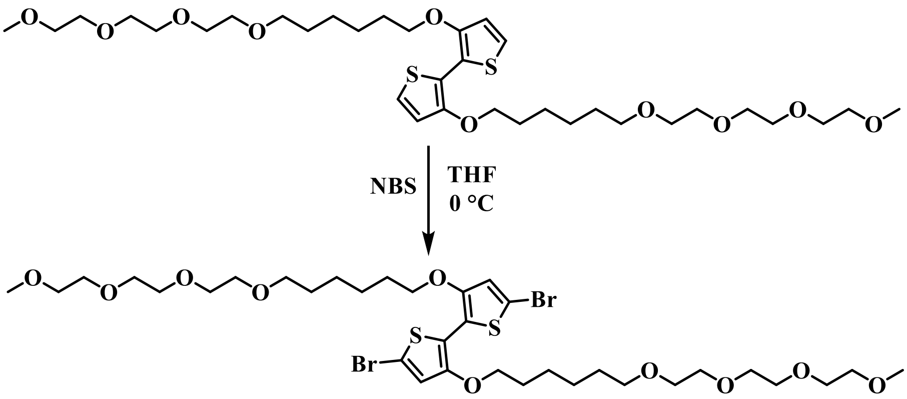

3,3'-bis((2,5,8,11-tetraoxaheptadecan-17-yl)oxy)-2,2'-bithiophene (600 mg, 0.87 mmol, 1.0 eq.) was dissolved in 40 mL anhydrous THF, cooled to 0 °C under a Nitrogen atmosphere. The reaction was covered and kept in the dark, N-bromosuccinimide (316.8 mg, 1.78 mmol, 2.05 eq.) was subsequently added in four equal portions, leaving 4 minutes between each addition. After stirring for 30 minutes the reaction was poured into water, washed with saturated sodium bicarbonate solution and quenched with sodium metabisulphite. The aqueous layer was extracted three times with DCM, the organic phases were consolidated and dried over Na_2SO_4. The crude material was purified by column chromatography, on silica gel, using ethyl acetate as the eluent to afford a yellow oil, which solidified at low temperatures (700 mg, 95%).

^{1}H NMR (400 MHz, Chloroform-*d*) δ 6.80 (s, 2H), 4.08 – 3.99 (m, 4H), 3.70 – 3.43 (m, 24H), 3.47 (t, *J* = 6.7 Hz, 4H), 3.37 (s, 6H), 1.87 – 1.78 (m, 4H), 1.67 – 1.57 (m, 4H), 1.56 – 1.37 (m, 8H).

^{13}C NMR (101 MHz, Chloroform-*d*) δ 149.38, 132.26, 128.76, 119.03, 72.23, 71.95, 71.31, 70.64, 70.60, 70.53, 70.12, 59.05, 29.58, 29.54, 29.47, 25.80.

Mass (MALDI-ToF): 848.9 $[M+H]^+$ (calc. 848.1 $C_{34}H_{56}Br_2O_{10}S_2$).

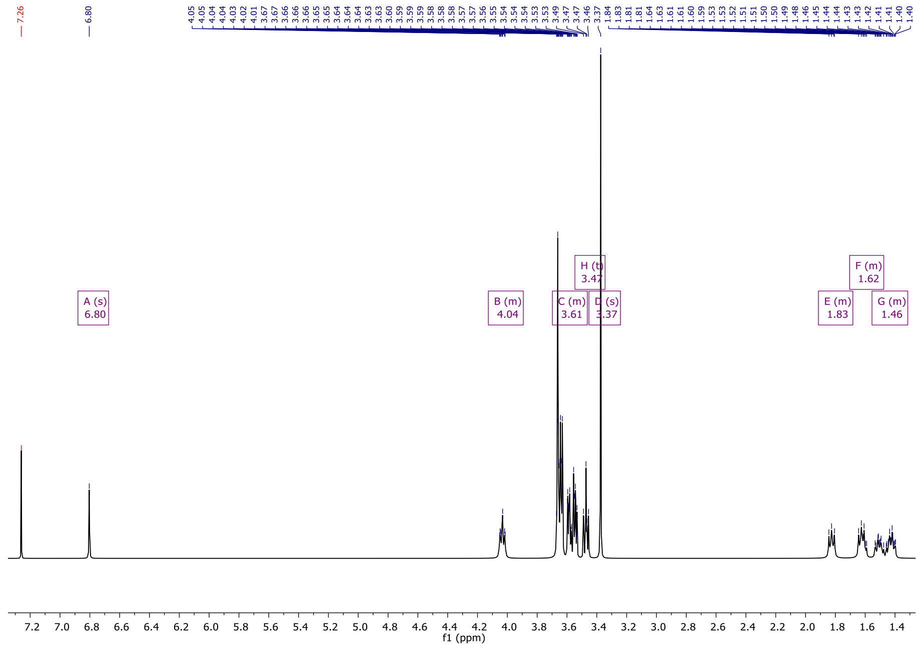

Figure S57. ^{1}H NMR in $CDCl_3$.

10.20. Synthesis of Polymer p(C6g$_3$T2-TT)

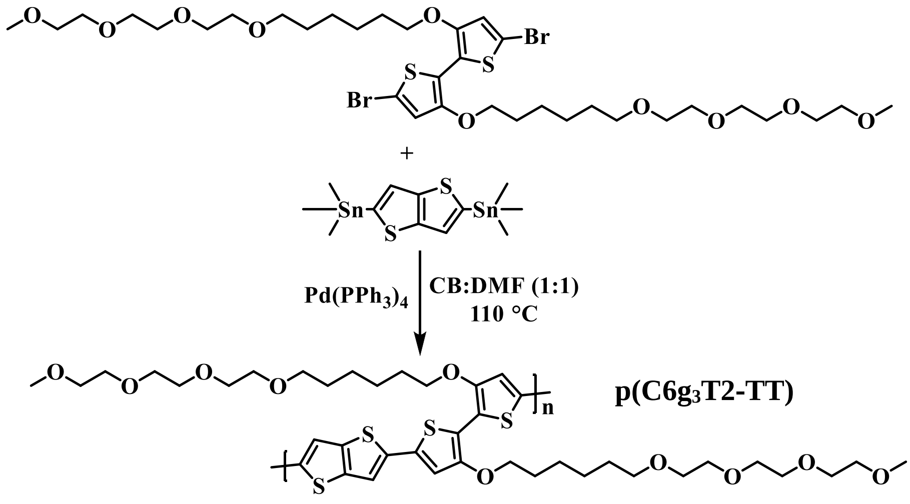

An oven dried 5 mL microwave vial was charged with 17,17'-((5,5'-dibromo-[2,2'-bithiophene]-3,3'-diyl)bis(oxy))bis(2,5,8,11-tetraoxaheptadecane) (200 mg, 0.24 mmol, 1.0 eq.), 2,5-bis(trimethylstannyl)thieno[3,2-*b*]thiophene (109.8 mg, 0.24 mmol, 1.0 eq.) and $Pd(PPh_3)_4$ (5.44 mg, 0.005 mmol, 0.02 eq.). Cap was sealed and the vial was purged with Nitrogen for 5 minutes prior to the addition of anhydrous DMF (1.57 mL) and anhydrous chlorobenzene (1.57 mL). The reaction was stirred overnight at 110 °C then cooled to room temperature, upon which the solution formed a purple gel. Crude polymer was precipitated into 100 mL methanol and subsequently filtered into a thimble. Purification was conducted via Soxhlet extraction, washing with hexane, methanol, acetone, ethyl acetate and chloroform (in that order). The chloroform fraction was collected, solvent removed under reduced pressure and re-precipitated into 100 mL methanol, filtered to yield a blue metallic film (163 mg, 81%). GPC (DMF, 40 °C): M_n 114.2 kDa, M_w 236.7 kDa, *Đ* 2.1.

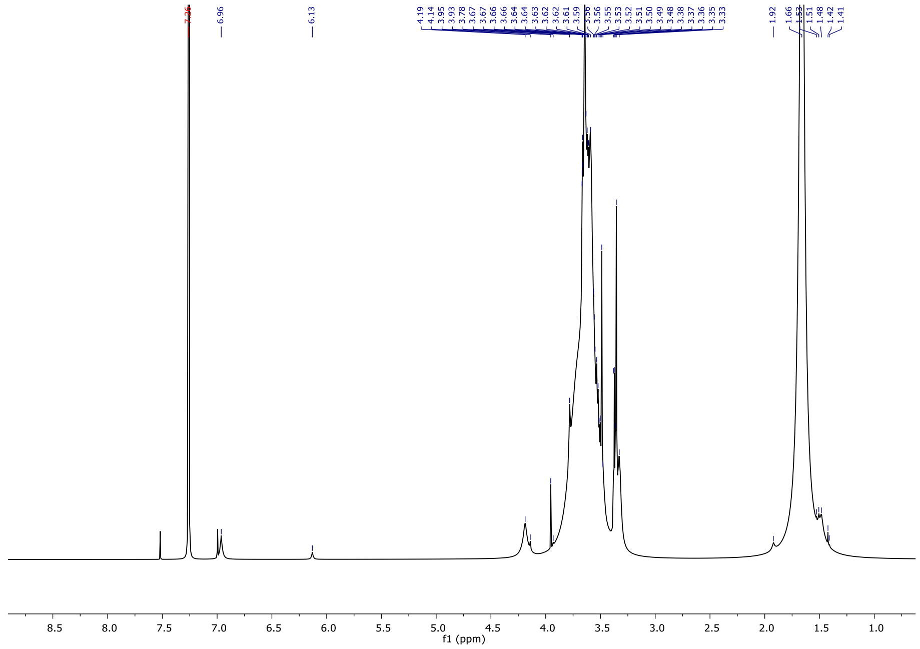

Figure S58. ^1H NMR in $CDCl_3$.

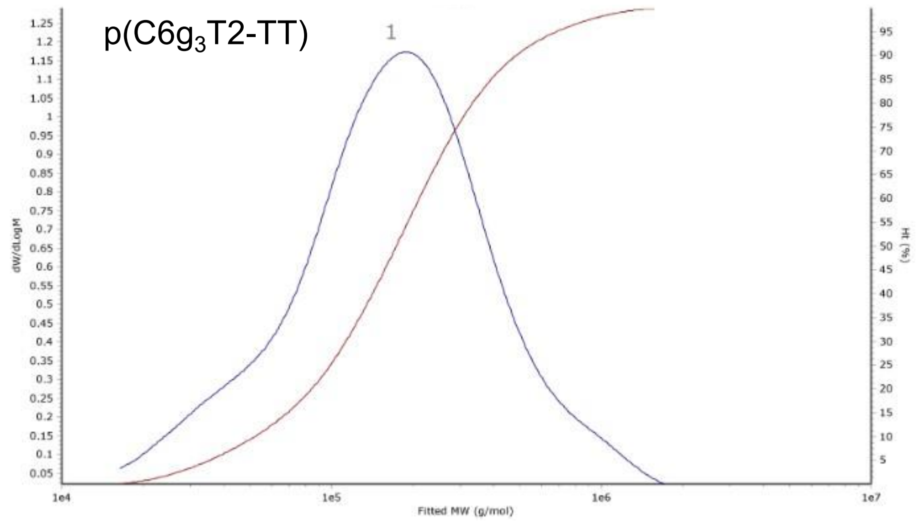

Figure S59. GPC spectra. M_n, M_w, and $Đ$ (M_w/M_n) were determined by GPC using low-D (<1.10) polystyrene standards and DMF as the eluent at 40 °C.

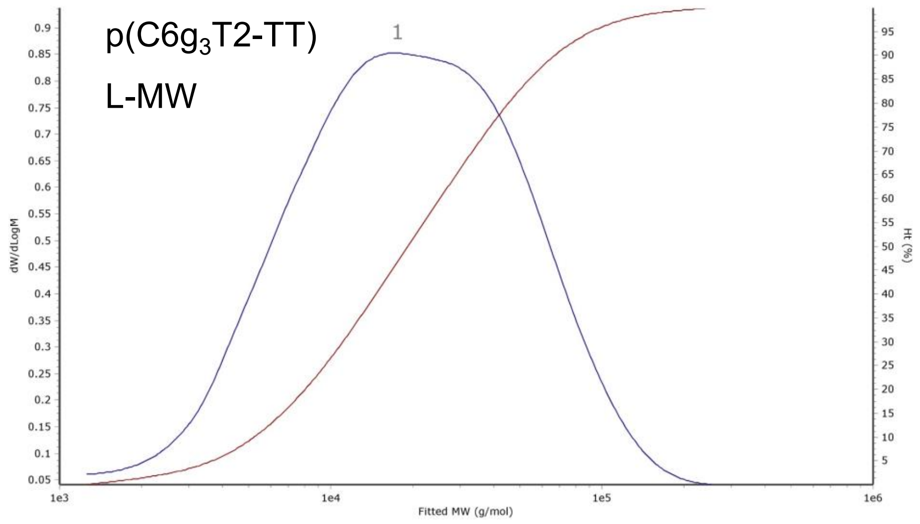

Figure S60. GPC spectra. M_n, M_w, and $Đ$ (M_w/M_n) were determined by GPC using low-D (<1.10) polystyrene standards and DMF as the eluent at 40 °C.

10.21. Synthesis of Polymer p(C6g$_3$T2-T)

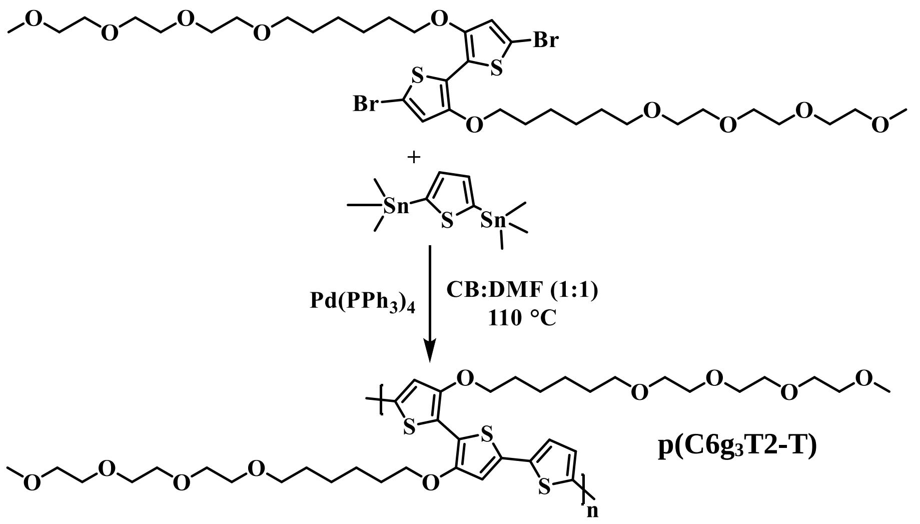

p(C6g$_3$T2-T)

An oven dried 5 mL microwave vial was charged with 17,17'-((5,5'-dibromo-[2,2'-bithiophene]-3,3'-diyl)bis(oxy))bis(2,5,8,11-tetraoxaheptadecane) (200 mg, 0.24 mmol, 1.0 eq.), 2,5-bis(trimethylstannyl)thiophene (96.6 mg, 0.24 mmol, 1.0 eq.) and Pd(PPh$_3$)$_4$ (5.44 mg, 0.005 mmol, 0.02 eq.). Cap was sealed and the vial was purged with Nitrogen for 5 minutes prior to the addition of anhydrous DMF (1.57 mL) and anhydrous chlorobenzene (1.57 mL). The reaction was stirred overnight at 110 °C then cooled to room temperature, upon which the solution formed a purple gel. Crude polymer was precipitated into 100 mL methanol and subsequently filtered into a thimble. Purification was conducted via Soxhlet extraction, washing with hexane, methanol, acetone, ethyl acetate and chloroform (in that order). The chloroform fraction was collected, solvent removed under reduced pressure and re-precipitated into 100 mL methanol, filtered to yield a blue metallic film (127 mg, 66%). GPC (DMF, 40 °C): M_n 28.0 kDa, M_w 59.2 kDa, *Đ* 2.1.

^{1}H NMR (400 MHz, Chloroform-*d*) δ 7.08 (s, 2H), 6.93 (s, 2H), 4.18 (s, 2H), 3.82 – 3.44 (m, 36H), 3.36 (s, 6H), 1.93 (t, *J* = 7.4 Hz, 4H), 1.76 – 1.37 (m, 12H).

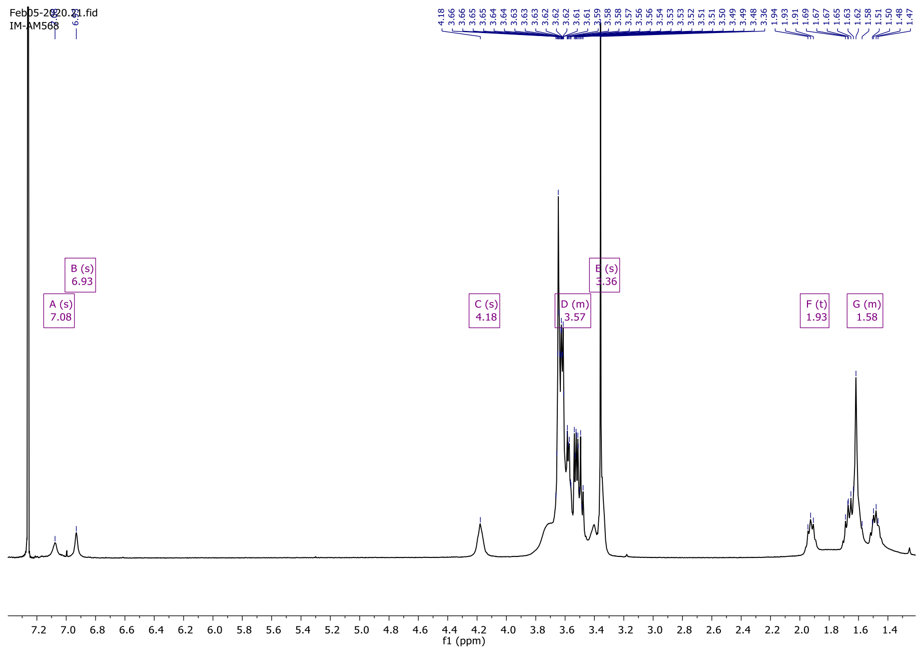

Figure S61. ^{1}H NMR in $CDCl_3$.

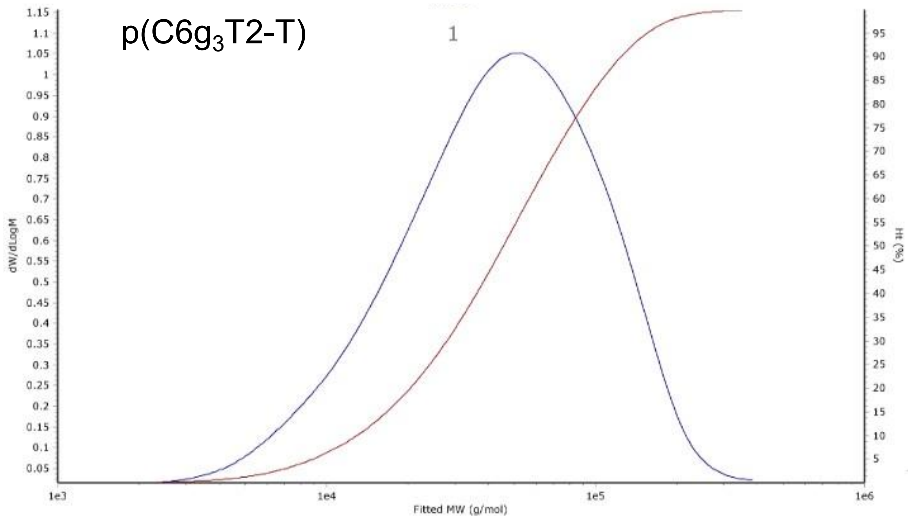

Figure S62. GPC spectra. M_n, M_w, and $Đ$ (M_w/M_n) were determined by GPC using low-D (<1.10) polystyrene standards and DMF as the eluent at 40 °C.

10.22. Synthesis of 8-(thiophen-3-yloxy)octan-1-ol

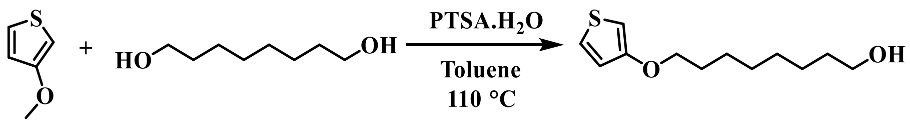

1,8-Octanediol (28.47 g, 194.7 mmol, 1.3 eq.) was dissolved in 200 mL toluene (required heating to reflux), followed by the addition of *p*-toluenesulfonic acid monohydrate (2.85 g, 15.0 mmol, 0.1 eq.). 3-Methoxythiophene (17.1 g, 149.8 mmol, 1.0 eq.) was added and the reaction was stirred at reflux (110 °C) for 90 minutes. Once cooled to room temperature the reaction was poured into water, the aqueous layer was extracted three times with DCM. The organic phases were combined, dried over Na_2SO_4 and solvent removed under reduced pressure. The crude alcohol was purified via column chromatography, on silica gel, using DCM as the eluent (Rf = 0.2). Product fractions were consolidated to afford a white fluffy solid (14 g, 41%).

^{1}H NMR (400 MHz, Chloroform-*d*) δ 7.16 (dd, *J* = 5.2, 3.1 Hz, 1H), 6.75 (dd, *J* = 5.2, 1.6 Hz, 1H), 6.22 (dd, *J* = 3.1, 1.5 Hz, 1H), 3.93 (t, *J* = 6.5 Hz, 2H), 3.64 (t, *J* = 6.6 Hz, 2H), 1.76 (dq, *J* = 8.2, 6.6 Hz, 2H), 1.51 – 1.41 (m, 4H), 1.44 – 1.30 (m, 6H).

^{13}C NMR (101 MHz, Chloroform-*d*) δ 158.16, 124.66, 119.66, 97.12, 70.35, 63.16, 32.89, 29.46, 29.38, 26.12, 25.81.

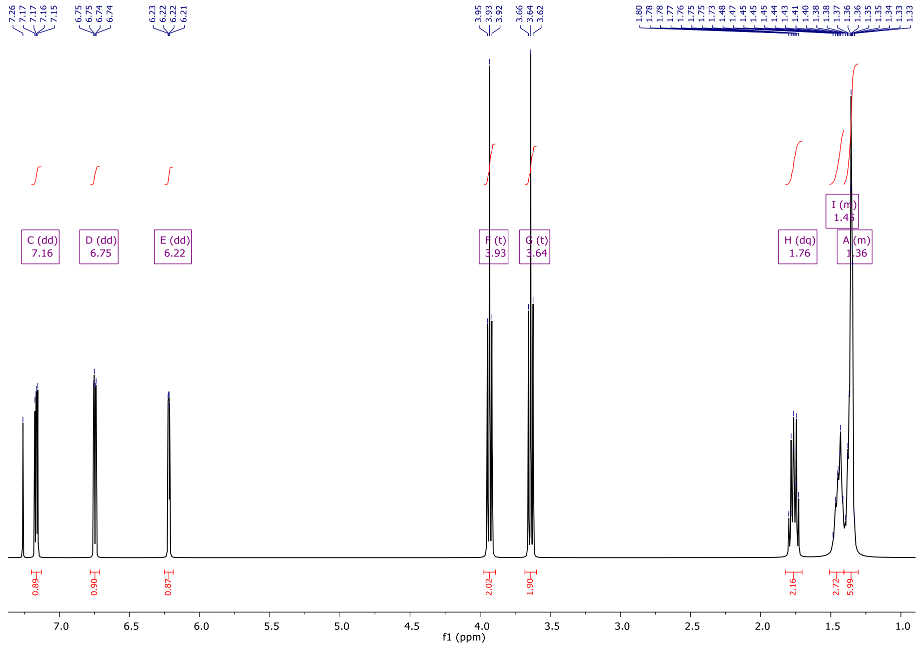

Figure S63. ^{1}H NMR in $CDCl_3$.

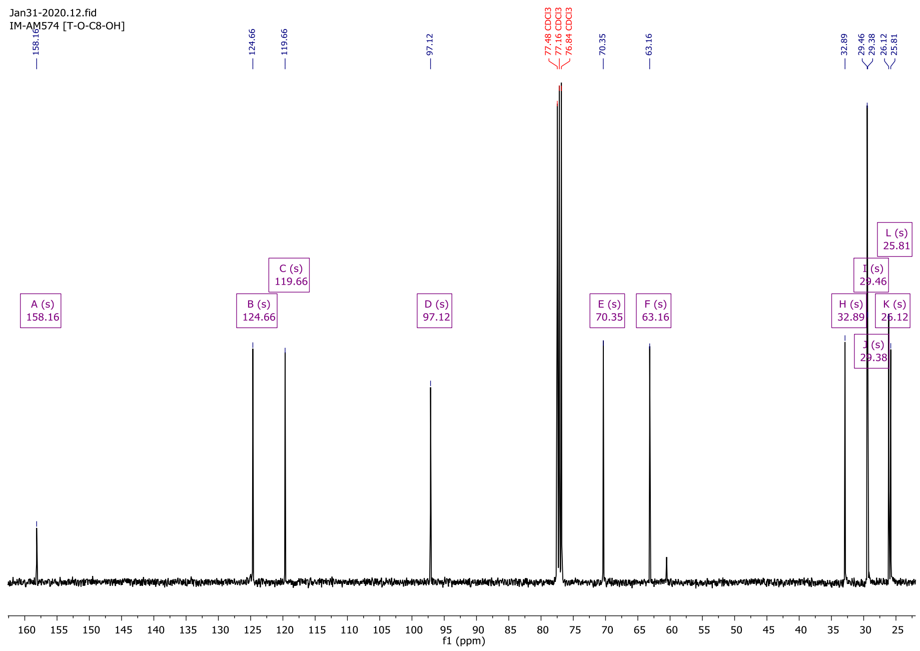

Figure S64. ^{13}C NMR in $CDCl_3$.

10.23. Synthesis of 8-(thiophen-3-yloxy)octyl 4-methylbenzenesulfonate

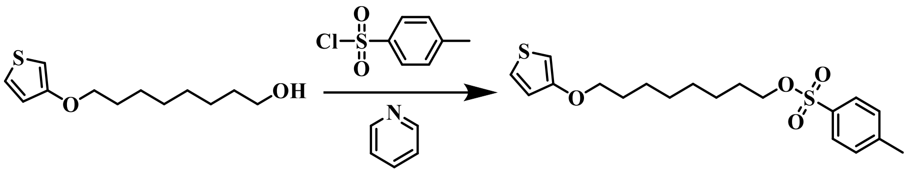

8-(thiophen-3-yloxy)octan-1-ol (14.0 g, 61.3 mmol, 1.0 eq.) was dissolved in 30 mL of pyridine, cooled to 0 °C. 4-Toluenesulfonyl chloride (14.0 g, 73.6 mmol, 1.2 eq.) was added as a single portion and the reaction was stirred vigorously for 20 minutes before being left in the refrigerator overnight. Afterwards, 2 M HCl (30 mL) was added, the reaction was stirred for 30 minutes before being poured into water, the aqueous phase was extracted three times with DCM. The organic layers were combined, dried over Na_2SO_4 and solvent removed under reduced pressure. The isolated product was used in the next step without further purification (19.5 g, 83%).

^{1}H NMR (400 MHz, Chloroform-*d*) δ 7.83 – 7.73 (m, 2H), 7.38 – 7.30 (m, 2H), 7.16 (dd, *J* = 5.3, 3.1 Hz, 1H), 6.78 – 6.69 (m, 1H), 6.25 – 6.18 (m, 1H), 4.01 (t, *J* = 6.5 Hz, 2H), 3.91 (t, *J* = 6.6 Hz, 2H), 2.44 (s, 3H), 1.50 – 1.21 (m, 12H).

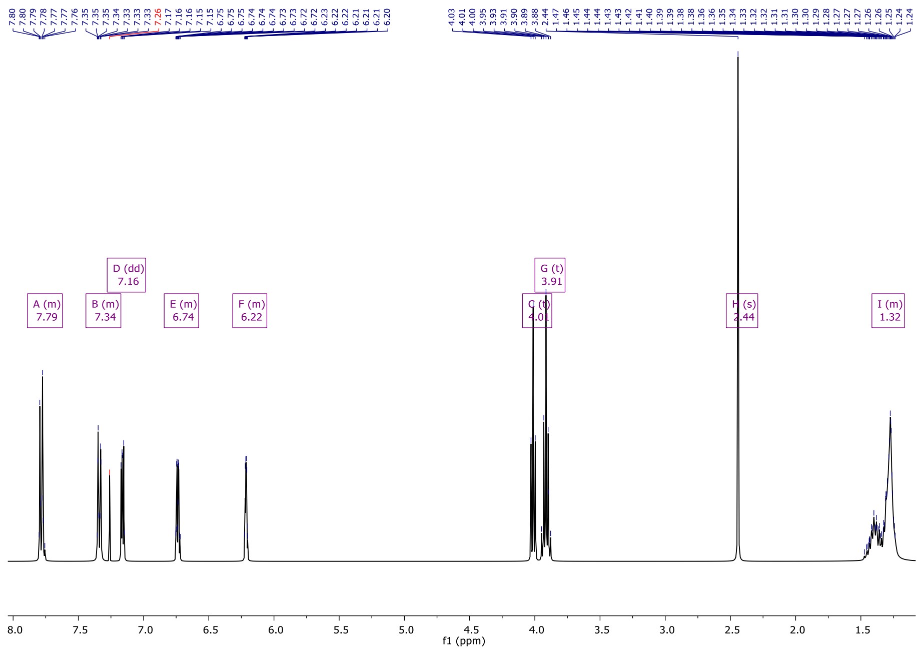

Figure S65. ^1H NMR in $CDCl_3$.

10.24. Synthesis of 19-(thiophen-3-yloxy)-2,5,8,11-tetraoxanonadecane

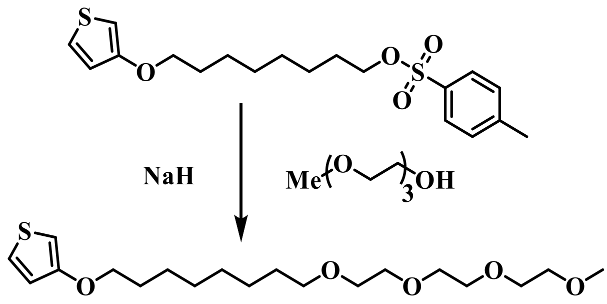

A 2-neck oven dried 250 mL RBF was charged with sodium hydride (60% in mineral oil) (5.93 g, 149 mmol, 3.0 eq.) and dissolved in anhydrous THF (60 mL) under a Nitrogen atmosphere. The suspension was cooled to 0 °C and triethylene glycol monomethyl ether (23.7 mL, 149 mmol, 3.0 eq.) was added slowly dropwise. The reaction was stirred for 30 minutes, until effervescence ceased, then 8-(thiophen-3-yloxy)octyl 4-methylbenzenesulfonate (19 g, 49.7 mmol, 1.0 eq.) was added. The reaction was stirred overnight before pouring into water, the aqueous phase was extracted three times with DCM. The organic layers were combined, dried over Na_2SO_4 and solvent removed under reduced pressure. The crude product was purified by column chromatography, on silica gel, using hexane:ethyl acetate (1:1) (v/v) as the eluent system to afford a yellow oil (11.5 g, 62%).

^1H NMR (400 MHz, Chloroform-*d*) δ 7.16 (dd, *J* = 5.2, 3.1 Hz, 1H), 6.74 (dd, *J* = 5.2, 1.6 Hz, 1H), 6.22 (dd, *J* = 3.1, 1.5 Hz, 1H), 3.92 (t, *J* = 6.5 Hz, 2H), 3.70 – 3.51 (m, 14H), 3.44 (t, *J* = 6.8 Hz, 2H), 3.38 (s, 3H), 1.75 (dq, *J* = 8.4, 6.6 Hz, 2H), 1.44 (q, *J* = 6.9 Hz, 2H), 1.33 (dq, *J* = 4.4, 2.7, 2.2 Hz, 8H).

^{13}C NMR (101 MHz, Chloroform-*d*) δ 158.16, 124.63, 119.66, 97.09, 72.09, 71.61, 70.77, 70.74, 70.67, 70.36, 70.20, 59.17, 29.75, 29.52, 29.45, 29.38, 26.17, 26.13.

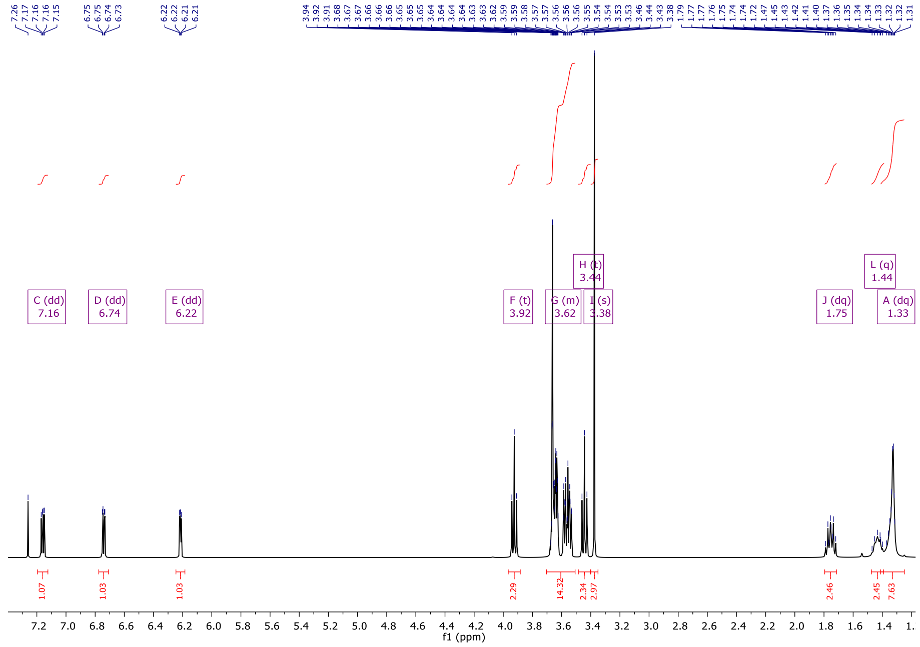

Figure S66. ^1H NMR in $CDCl_3$.

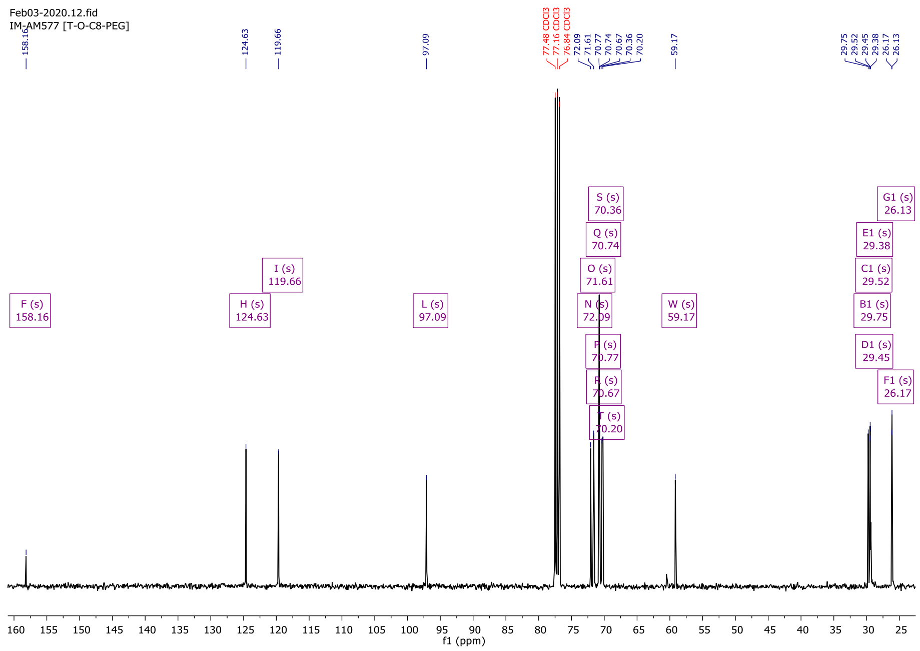

Figure S67. ^{13}C NMR in $CDCl_3$.

10.25. Synthesis of 19-((2-bromothiophen-3-yl)oxy)-2,5,8,11-tetraoxanonadecane

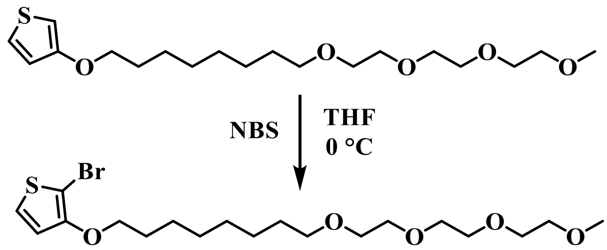

19-(thiophen-3-yloxy)-2,5,8,11-tetraoxanonadecane (4.5 g, 12.0 mmol, 1.0 eq.) was added to an oven-dried 2-neck 100 mL RBF, under a Nitrogen atmosphere. Dry THF (20 mL) was added, vessel cooled to 0 °C in the dark. N-bromosuccinimide (4 × 545 mg, 12.3 mmol, 1.02 eq.) was added portion wise, leaving approximately 4 minutes between each addition. The reaction was stirred for 30 minutes before being poured into water, washed with saturated sodium bicarbonate solution and quenched with sodium metabisulphite. The aqueous phase was extracted three times with DCM, the organic phases were combined and dried over Na_2SO_4. The crude product was purified via column chromatography, on silica gel, using 1:1 hexane:ethyl acetate (v/v) as the eluent system to afford a yellow oil (5.23 g, 96%).

^1H NMR (400 MHz, Chloroform-*d*) δ 7.18 (dd, *J* = 6.0, 1.1 Hz, 1H), 6.73 (dd, *J* = 6.0, 1.1 Hz, 1H), 4.02 (td, *J* = 6.6, 1.2 Hz, 2H), 3.69 – 3.51 (m, 12H), 3.44 (td, *J* = 6.8, 1.0 Hz, 2H), 3.37 (s, 3H), 1.80 – 1.68 (m, 2H), 1.45 (dd, *J* = 10.4, 4.1 Hz, 2H), 1.45 – 1.30 (m, 8H).

^{13}C NMR (101 MHz, Chloroform-*d*) δ 154.67, 124.27, 117.67, 91.75, 72.35, 72.09, 71.62, 70.77, 70.73, 70.67, 70.20, 59.18, 29.75, 29.71, 29.59, 29.50, 29.39, 26.15, 25.90.

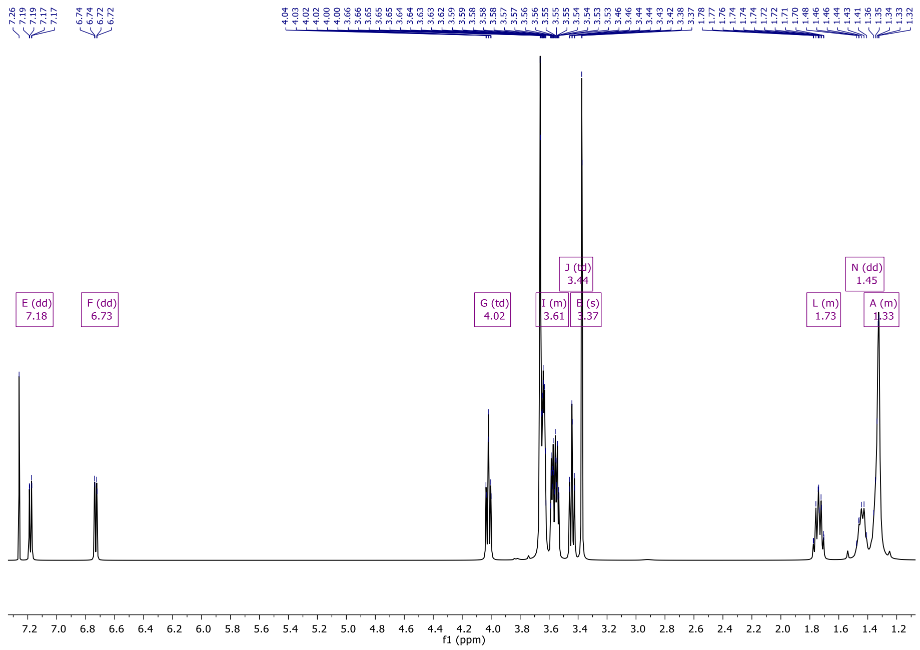

Figure S68. ^{1}H NMR in $CDCl_3$.

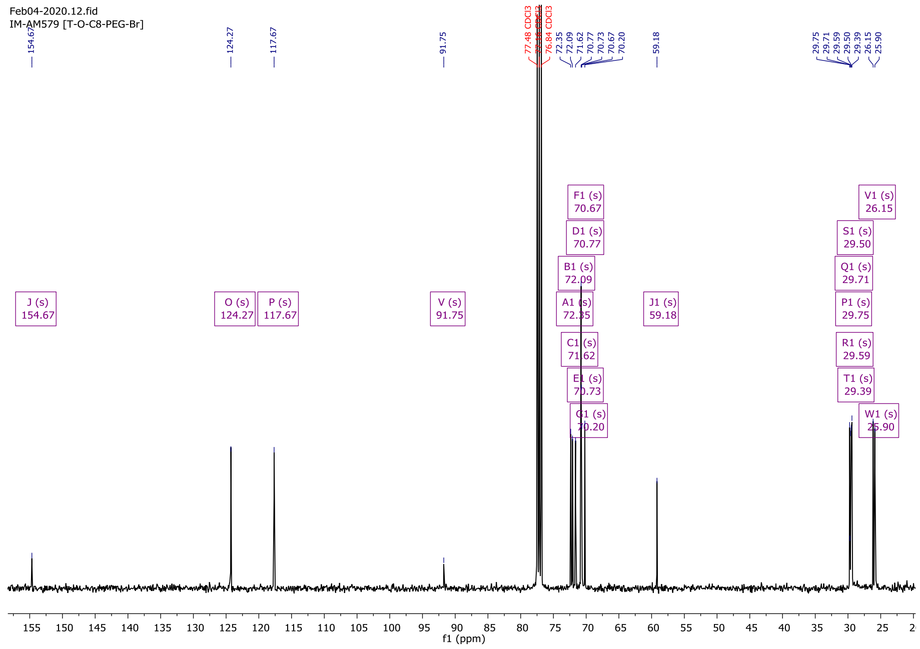

Figure S69. ^{13}C NMR in $CDCl_3$.

10.26. Synthesis of 3,3'-bis((2,5,8,11-tetraoxanonadecan-19-yl)oxy)-2,2'-bithiophene

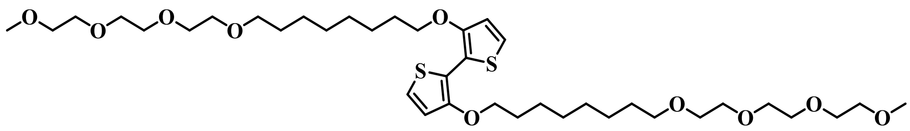

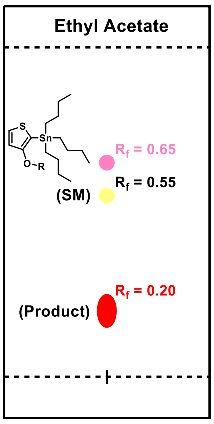

19-((2-bromothiophen-3-yl)oxy)-2,5,8,11-tetraoxanonadecane (4 g, 8.82 mmol, 1.0 eq.) was added to an oven dried 2-neck 250 mL RBF, under a Nitrogen atmosphere. Anhydrous chlorobenzene (22 mL) was added, followed by anhydrous DMF (22 mL). Bis(tributyltin) (2.45 mL, 4.85 mmol, 0.55 eq.) was slowly added dropwise, the reaction was heated to 100 °C and $Pd(PPh_3)_4$ (713 mg, 0.62 mmol, 0.07 eq.) was added in a single portion. The mixture was stirred overnight and subsequently cooled to room temperature then passed through a short silica plug, eluting with ethyl acetate. Most tin by-products remained on the baseline and were carefully disposed of as solid tin waste. Solvent was removed under reduced pressure and thin-layer chromatography, using ethyl acetate as the eluent, showed three distinct spots. Co-spotting with (2.25) shows the 2nd spot (Rf = 0.55) correlates to the starting material, the top spot (Rf = 0.65) corresponds to unreacted tin intermediate and the desired product spot resided at Rf = 0.20. The crude residue was hence purified by column chromatography, on silica gel, using ethyl acetate as the eluent. Product fractions were combined to yield a yellow oil, which solidified under reduced temperatures (1.05 g, 32%).

^{1}H NMR (400 MHz, Chloroform-*d*) δ 7.07 (d, *J* = 5.6 Hz, 2H), 6.83 (d, *J* = 5.5 Hz, 2H), 4.08 (t, *J* = 6.5 Hz, 4H), 3.69 – 3.60 (m, 18H), 3.61 – 3.51 (m, 10H), 3.44 (t, *J* = 6.8 Hz, 4H), 3.37 (s, 6H), 1.88 – 1.79 (m, 4H), 1.52 (td, *J* = 7.7, 2.6 Hz, 4H), 1.34 (qd, *J* = 6.3, 5.6, 3.1 Hz, 12H).

^{13}C NMR (101 MHz, Chloroform-*d*) δ 151.75, 132.04, 131.94, 128.45, 128.33, 121.45, 115.87, 71.78, 71.33, 70.47, 70.43, 70.36, 69.89, 58.87, 29.52, 29.46, 29.21, 29.13, 25.87, 25.84.

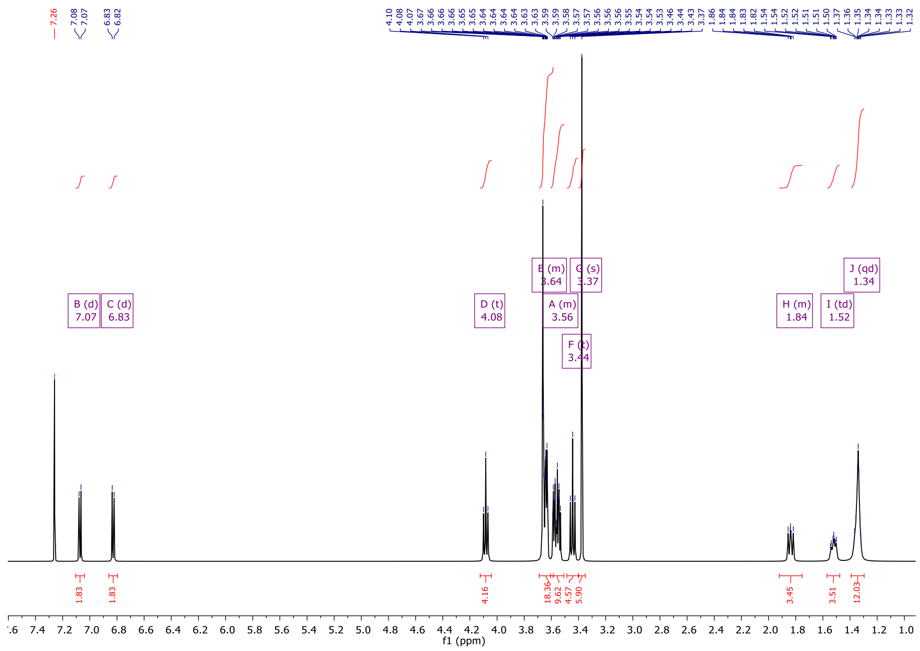

Figure S70. ^{1}H NMR in $CDCl_3$.

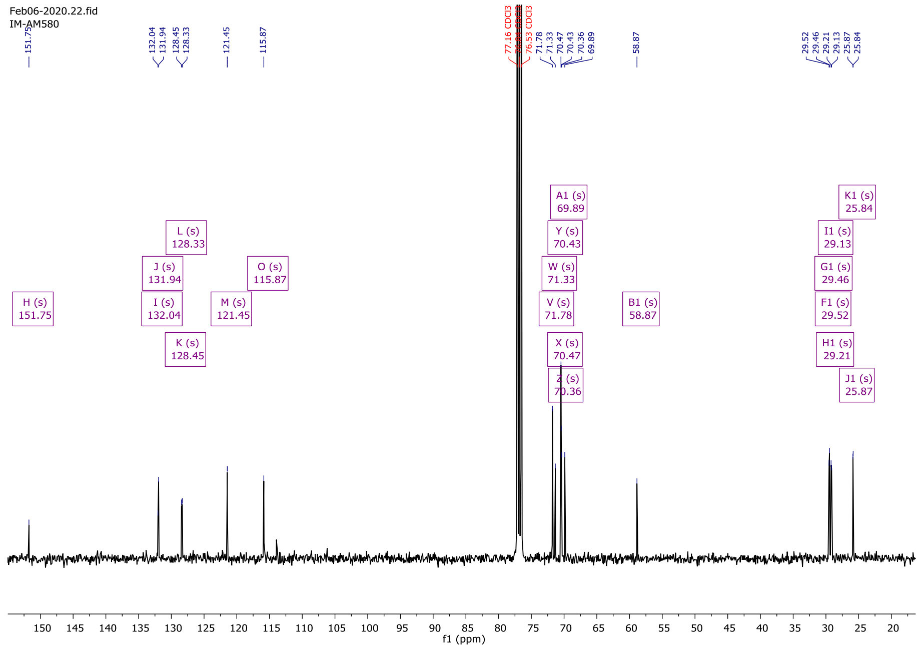

Figure S71. ^{13}C NMR in $CDCl_3$.

10.27. Synthesis of 19,19'-((5,5'-dibromo-[2,2'-bithiophene]-3,3'-diyl)bis(oxy))bis(2,5,8,11-tetraoxanonadecane)

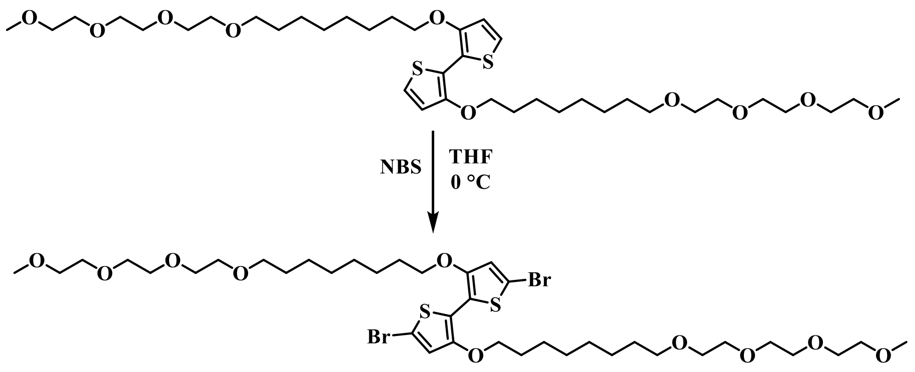

3,3'-bis((2,5,8,11-tetraoxanonadecan-19-yl)oxy)-2,2'-bithiophene (800 mg, 1.07 mmol, 1.0 eq.) was dissolved in 40 mL of anhydrous THF and cooled to 0 °C. The solution was covered and kept in the dark, N-bromosuccinimide (400.2 mg, 2.25 mmol, 2.1 eq.) was subsequently added in 4 even portions, with approximately 4 minutes between each addition. The reaction was stirred for 30 minutes before being poured into water, washed with saturated sodium bicarbonate solution and quenched with sodium metabisulphite. The aqueous phase was extracted three times with DCM, the organic phases were combined and dried over Na_2SO_4 before the solvent was removed under reduced pressure. The crude oil was purified via column chromatography, on silica gel, using 1:1 hexane:ethyl acetate (v/v) as the eluent system. Product fractions were combined, and solvent removed to afford a yellow oil, which solidified at reduced temperature (948 mg, 98%).

^{1}H NMR (400 MHz, Chloroform-*d*) δ 6.80 (s, 2H), 4.03 (t, *J* = 6.5 Hz, 4H), 3.69 – 3.51 (m, 24H), 3.45 (t, *J* = 6.8 Hz, 4H), 3.37 (s, 6H), 1.89 – 1.75 (m, 6H), 1.65 – 1.54 (m, 4H), 1.42 – 1.30 (m, 14H).

^{13}C NMR (101 MHz, Chloroform-*d*) δ 227.02, 199.51, 150.45, 132.40, 132.31, 128.80, 128.68, 119.18, 115.24, 109.98, 72.47, 72.10, 71.63, 70.78, 70.74, 70.67, 70.21, 68.12, 59.18, 29.77, 29.63, 29.50, 29.35, 26.16, 26.02, 25.76.

Mass (MALDI-ToF): 904.9 $[M+H]^{+}$ (calc. 904.2 $C_{38}H_{64}Br_2O_{10}S_2$).

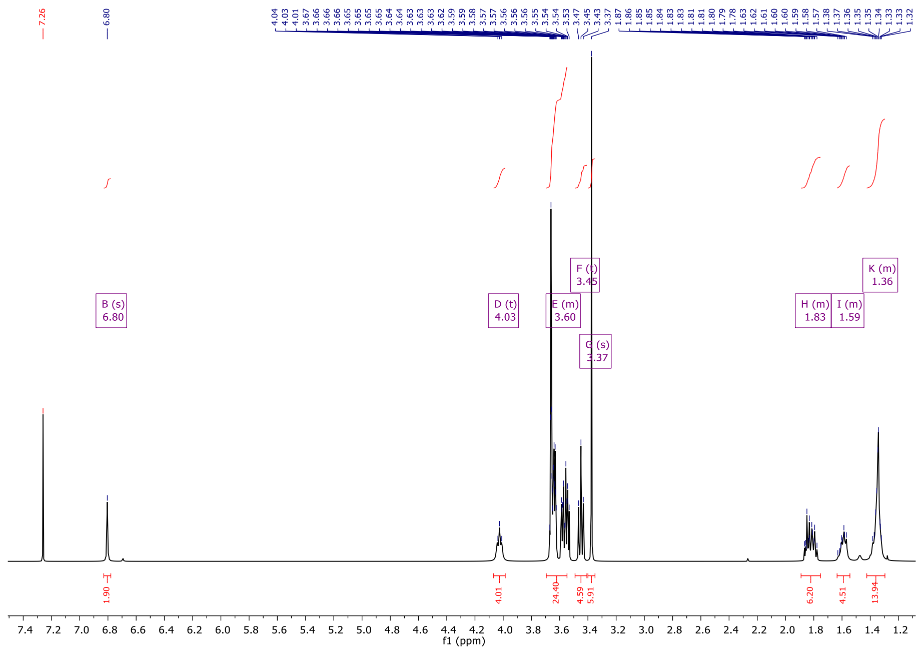

Figure S72. ^{1}H NMR in $CDCl_3$.

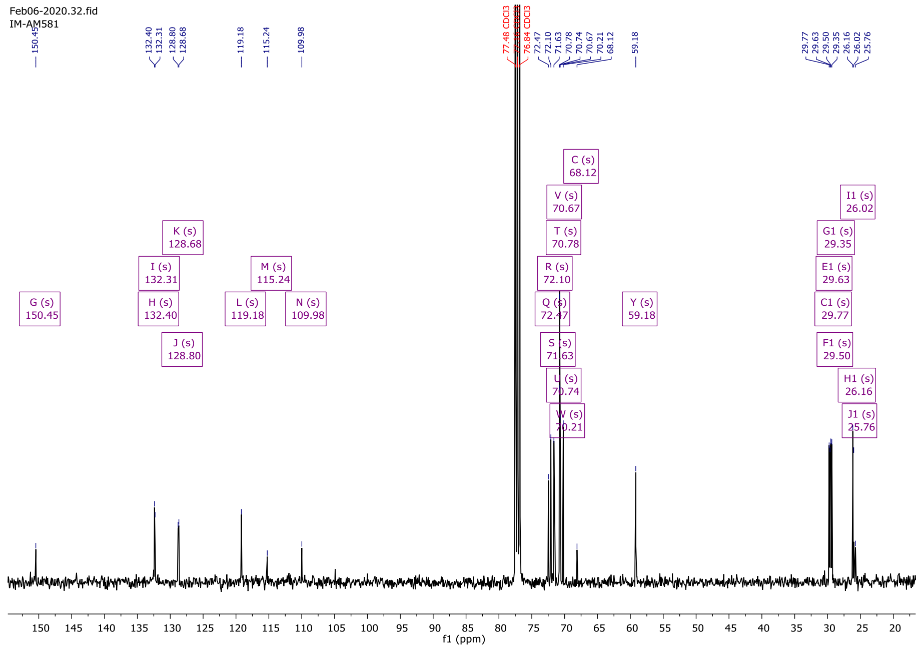

Figure S73. ^{13}C NMR in $CDCl_3$.

10.28. Synthesis of Polymer p(C8g$_3$T2-TT)

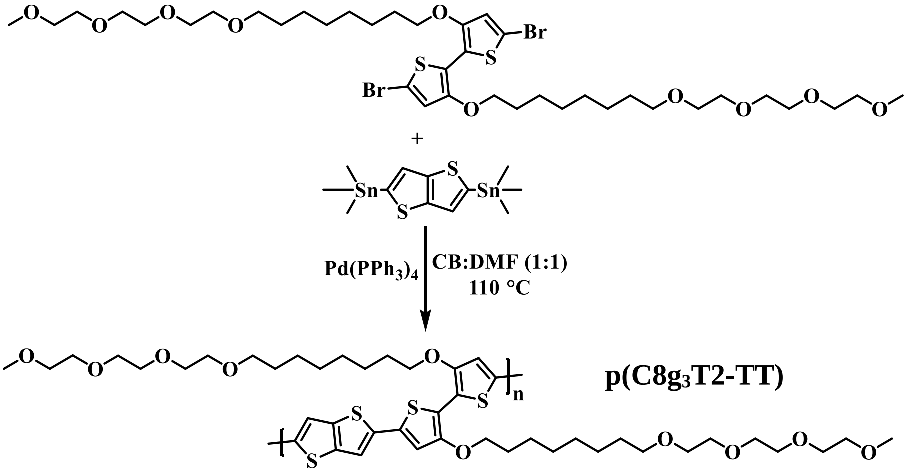

An oven dried 5 mL microwave vial was charged with 19,19'-((5,5'-dibromo-[2,2'-bithiophene]-3,3'-diyl)bis(oxy))bis(2,5,8,11-tetraoxanonadecane) (200 mg, 0.22 mmol, 1.0 eq.), 2,5-bis(trimethylstannyl)thieno[3,2-*b*]thiophene (103 mg, 0.22 mmol, 1.0 eq.) and $Pd(PPh_3)_4$ (5.11 mg, 0.004 mmol, 0.02 eq.). Cap was sealed and the vial was purged with Nitrogen for 5 minutes prior to the addition of anhydrous DMF (1.47 mL) and anhydrous chlorobenzene (1.47 mL). The reaction was stirred overnight at 110 °C then cooled to room temperature, upon which the solution formed a purple gel. Crude polymer was precipitated into 100 mL methanol and subsequently filtered into a thimble. Purification was conducted via Soxhlet extraction, washing with hexane, methanol, acetone, ethyl acetate and chloroform (in that order). The chloroform fraction was collected, solvent removed under reduced pressure and re-precipitated into 100 mL methanol, filtered to yield a blue metallic film (148 mg, 73%). GPC (DMF, 40 °C): M_n 110.6 kDa, M_w 282.1 kDa, $Đ$ 2.6.

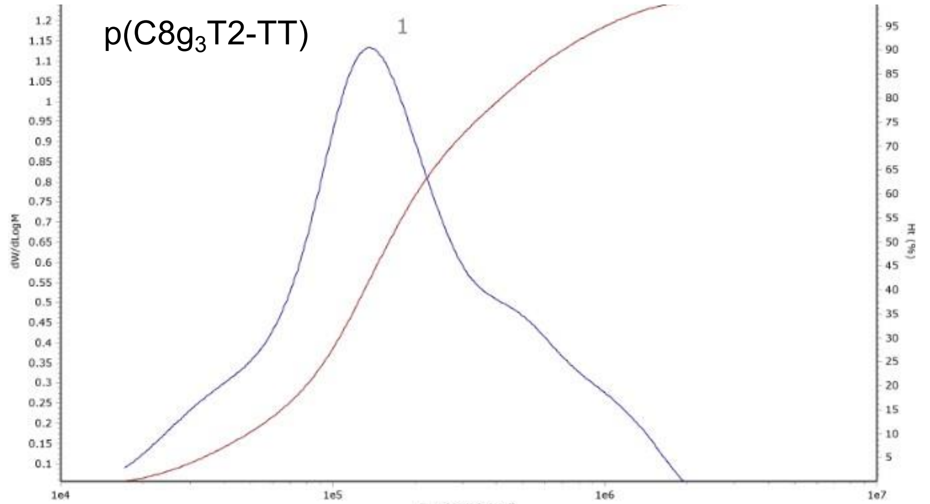

Figure S74. GPC spectra. M_n, M_w, and $Đ$ (M_w/M_n) were determined by GPC using low-Đ (<1.10) polystyrene standards and DMF as the eluent at 40 °C.

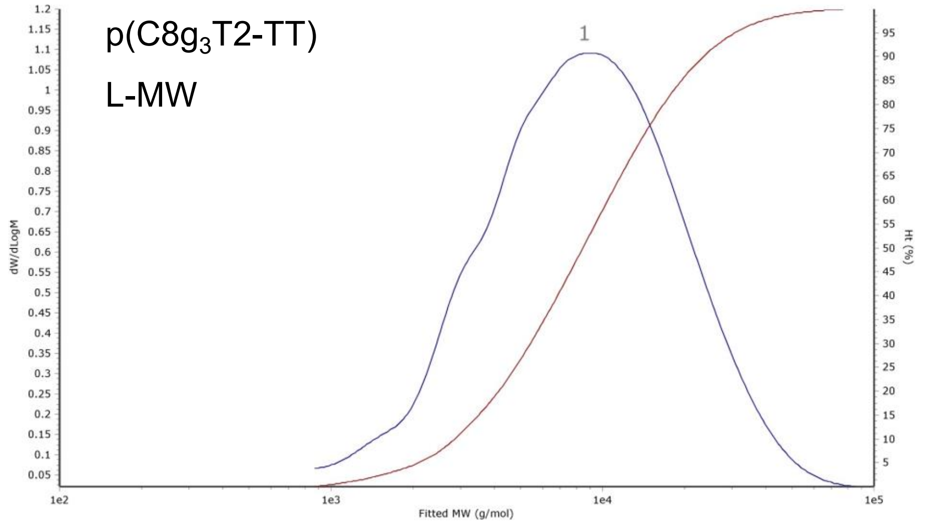

Figure S75. GPC spectra. M_n, M_w, and $Đ$ (M_w/M_n) were determined by GPC using low-Đ (<1.10) polystyrene standards and DMF as the eluent at 40 °C.

10.29. Synthesis of Polymer p(C8g$_3$T2-T)

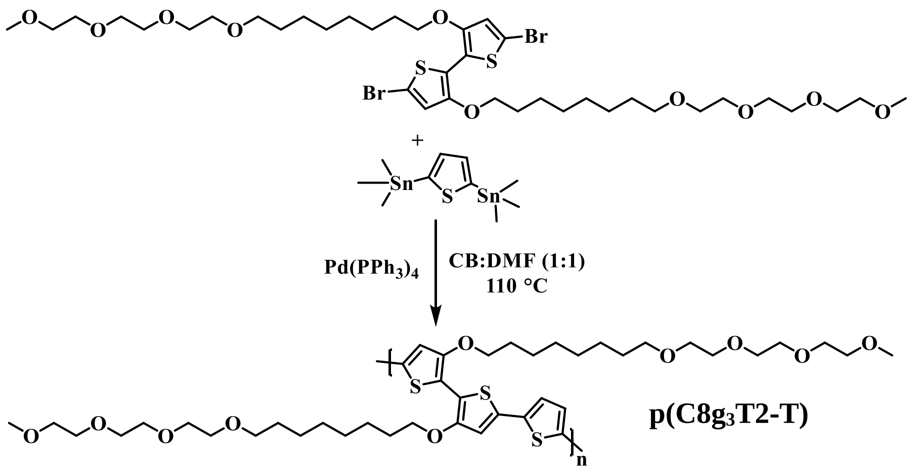

An oven dried 5 mL microwave vial was charged with 19,19'-((5,5'-dibromo-[2,2'-bithiophene]-3,3'-diyl)bis(oxy))bis(2,5,8,11-tetraoxanonadecane) (200 mg, 0.22 mmol, 1.0 eq.), 2,5-bis(trimethylstannyl)thiophene (90.57 mg, 0.22 mmol, 1.0 eq.) and $Pd(PPh_3)_4$ (5.11 mg, 0.004 mmol, 0.02 eq.). Cap was sealed and the vial was purged with Nitrogen for 5 minutes prior to the addition of anhydrous DMF (1.47 mL) and anhydrous chlorobenzene (1.47 mL). The reaction was stirred overnight at 110 °C then cooled to room temperature, upon which the solution formed a purple gel. Crude polymer was precipitated into 100 mL methanol and subsequently filtered into a thimble. Purification was conducted via Soxhlet extraction, washing with hexane, methanol, acetone, ethyl acetate and chloroform (in that order). The chloroform fraction was collected, solvent removed under reduced

pressure and re-precipitated into 100 mL methanol, filtered to yield a blue metallic film (74 mg, 39%). GPC (DMF, 40 °C): M_n 13.3 kDa, M_w 30.0 kDa, *Đ* 2.3.

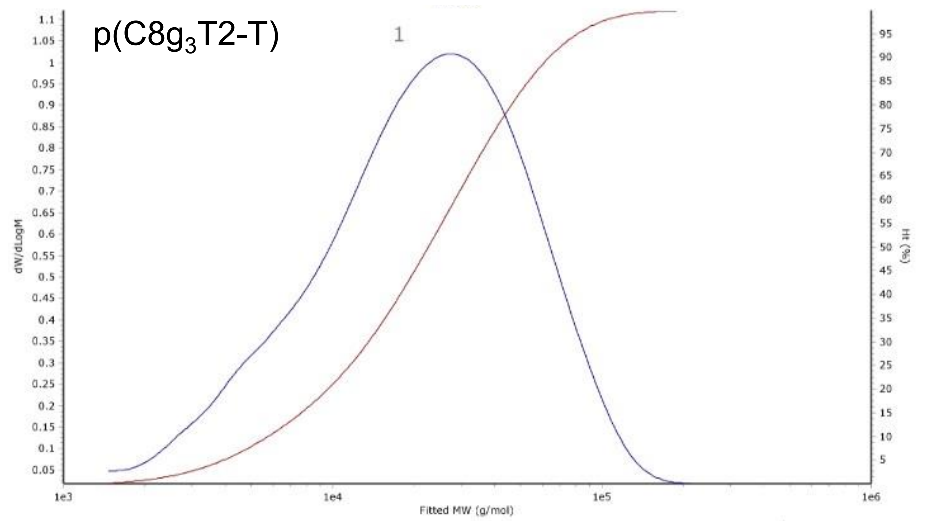

Figure S76. GPC spectra. M_n, M_w, and *Đ* (M_w/M_n) were determined by GPC using low-D (<1.10) polystyrene standards and DMF as the eluent at 40 °C.

References

(1) Ilavsky, J. Nika : Software for Two-Dimensional Data Reduction. *J. Appl. Crystallogr.* **2012**, *45* (2), 324–328.

(2) Oosterhout, S. D.; Savikhin, V.; Zhang, J.; Zhang, Y.; Burgers, M. A.; Marder, S. R.; Bazan, G. C.; Toney, M. F. Mixing Behavior in Small Molecule:Fullerene Organic Photovoltaics. *Chem. Mater.* **2017**, *29* (7), 3062–3069.

(3) Jiang, Z. GIXSGUI : A MATLAB Toolbox for Grazing-Incidence X-Ray Scattering Data Visualization and Reduction, and Indexing of Buried Three-Dimensional Periodic Nanostructured Films. *J. Appl. Crystallogr.* **2015**, *48* (3), 917–926.

(4) Klauk, H. Organic Thin-Film Transistors. *Chem. Soc. Rev.* **2010**, *39* (7), 2643.

(5) Bidinger, S. L.; Han, S.; Malliaras, G. G.; Hasan, T. Highly Stable PEDOT:PSS Electrochemical Transistors. *Appl. Phys. Lett.* **2022**, *120* (7), 073302.

(6) Rivnay, J.; Ramuz, M.; Leleux, P.; Hama, A.; Huerta, M.; Owens, R. M. Organic Electrochemical Transistors for Cell-Based Impedance Sensing. *Appl. Phys. Lett.* **2015**, *106* (4), 043301.

(7) Boyd, S.; Ganeshan, K.; Tsai, W.-Y.; Wu, T.; Saeed, S.; Jiang, D.; Balke, N.; van Duin, A. C.

T.; Augustyn, V. Effects of Interlayer Confinement and Hydration on Capacitive Charge Storage in Birnessite. *Nat. Mater.* **2021**, *20* (12), 1689–1694.

(8) Moia, D.; Giovannitti, A.; Szumska, A. A.; Maria, I. P.; Rezasoltani, E.; Sachs, M.; Schnurr, M.; Barnes, P. R. F.; McCulloch, I.; Nelson, J. Design and Evaluation of Conjugated Polymers with Polar Side Chains as Electrode Materials for Electrochemical Energy Storage in Aqueous Electrolytes. *Energy Environ. Sci.* **2019**, *12* (4), 1349–1357.

(9) Szumska, A. A.; Maria, I. P.; Flagg, L. Q.; Savva, A.; Surgailis, J.; Paulsen, B. D.; Moia, D.; Chen, X.; Griggs, S.; Mefford, J. T.; Rashid, R. B.; Marks, A.; Inal, S.; Ginger, D. S.; Giovannitti, A.; Nelson, J. Reversible Electrochemical Charging of N-Type Conjugated Polymer Electrodes in Aqueous Electrolytes. *J. Am. Chem. Soc.* **2021**, *143* (36), 14795–14805.

(10) Abraham, M. J.; Murtola, T.; Schulz, R.; Páll, S.; Smith, J. C.; Hess, B.; Lindahl, E. GROMACS: High Performance Molecular Simulations through Multi-Level Parallelism from Laptops to Supercomputers. *SoftwareX* **2015**, *1–2*, 19–25.

(11) Moro, S.; Siemons, N.; Drury, O.; Warr, D. A.; Moriarty, T. A.; Perdigão, L. M. A.; Pearce, D.; Moser, M.; Hallani, R. K.; Parker, J.; McCulloch, I.; Frost, J. M.; Nelson, J.; Costantini, G. The Effect of Glycol Side Chains on the Assembly and Microstructure of Conjugated Polymers. *ACS Nano* **2022**, *16* (12), 21303–21314.

(12) Siemons, N.; Pearce, D.; Cendra, C.; Yu, H.; Tuladhar, S. M.; Hallani, R. K.; Sheelamanthula, R.; LeCroy, G. S.; Siemons, L.; White, A. J. P.; McCulloch, I.; Salleo, A.; Frost, J. M.; Giovannitti, A.; Nelson, J. Impact of Side-Chain Hydrophilicity on Packing, Swelling, and Ion Interactions in Oxy-Bithiophene Semiconductors. *Adv. Mater.* **2022**, *34* (39), 2204258.

(13) Bussi, G.; Donadio, D.; Parrinello, M. Canonical Sampling through Velocity Rescaling. *J. Chem. Phys.* **2007**, *126* (1), 14101.

(14) York, D. M.; Darden, T. A.; Pedersen, L. G. The Effect of Long-Range Electrostatic Interactions in Simulations of Macromolecular Crystals: A Comparison of the Ewald and Truncated List Methods. *J. Chem. Phys.* **1993**, *99* (10), 8345–8348.

(15) Essmann, U.; Perera, L.; Berkowitz, M. L.; Darden, T.; Lee, H.; Pedersen, L. G. A Smooth Particle Mesh Ewald Method. *J. Chem. Phys.* **1995**, *103* (19), 8577–8593.
